# Discovery of Novel Ferrites for Thermochemical H_2_ Production Cycle via High‐Throughput Thermodynamic Screening

**DOI:** 10.1002/advs.202501846

**Published:** 2025-06-25

**Authors:** Dongkyu Lee, Joonhyun Nam, Byeong‐Gyu Park, Hyemin Kim, In‐Ho Jung, Hyungyu Jin

**Affiliations:** ^1^ Department of Mechanical Engineering Pohang University of Science and Technology (POSTECH) Pohang 37673 Republic of Korea; ^2^ Department of Materials Science and Engineering Seoul National University Seoul 08826 Republic of Korea; ^3^ Pohang Accelerator Laboratory (PAL) Pohang University of Science and Technology (POSTECH) Pohang 37673 Republic of Korea; ^4^ Research Institute of Advanced Materials Seoul National University Seoul 08826 Republic of Korea

**Keywords:** CALPHAD, high‐throughput screening, multi‐cation oxides, thermochemistry, water splitting

## Abstract

This study highlights the discovery of the (MgMnCo)_1‐x_Fe_x_O_y_ system, identified through a high‐throughput thermodynamic screening utilizing the CALPHAD methodology, as a highly efficient material system for two‐step thermochemical water splitting under mitigated redox reaction conditions. The screening focuses on a wide range of ferrite systems (M_1‐x_Fe_x_O_y_, M═Mn, Al, Mg, Co, Ni, 0.05< x< 0.95) and particularly underscores the (MgMnCo)_1‐x_Fe_x_O_y_ system for its high oxygen capacity during redox cycles. Remarkably, (MgMnCo)_0.65_Fe_0.35_O_y_ achieves state‐of‐the‐art turnover number and a higher theoretical solar‐to‐fuel efficiency of 43.6% compared to benchmark ceria at 17.4%. CALPHAD‐based thermodynamic analysis and spectroscopic electronic structure analysis reveal that the excellent redox performance of this material is attributed to the simultaneous contributions of Fe and Mn cations. This dual active feature is discovered for the first time in any ferrite cycles to the best of available knowledge, offering significant opportunities to explore new multi‐cation ferrite systems in the related research field. In addition, the screening methodology employed here can potentially be extended to other thermochemical processes.

## Graphical abstract

A high‐throughput CALPHAD‐based approach is employed to discover multicomponent ferrites with enhanced H_2_ yields for thermochemical water splitting. The most promising composition exhibits exceptional redox performance and favorable thermodynamics, with dual redox activity of Fe and Mn. These findings offer broad applicability to next‐generation redox systems and other thermochemical reactions.

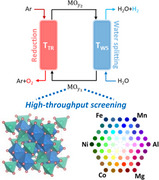

## 1 Introduction

As global warming and climate change intensify, the imperative for decarbonizing industries worldwide becomes increasingly pronounced. Industrial sectors such as the steel industry, ammonia synthesis, and international bunkers (including maritime and aviation fuels) are under strong pressure to reduce their carbon footprints, contributing ≈8%, 1%, and 3.1%, respectively, to global carbon emissions.^[^ ^1^, ^2^, ^3^ ^]^ In the quest for decarbonization within these sectors, hydrogen is emerging as a pivotal clean energy carrier and an essential precursor material, putting significant momentum into developing CO_2_‐free hydrogen production technologies. Among the various technologies being developed, including photochemical,^[^ ^4^, ^5^ ^]^ electrochemical,^[^ ^6^, ^7^, ^8^, ^9^ ^]^ and thermochemical pathways,^[^ ^10^, ^11^, ^12^, ^13^ ^]^ the thermochemical pathway, in particular, is garnering considerable attention for its economic viability and scalability. One notable application of thermochemical technology is at the pilot scale, where it has shown promise in producing H_2_ and CO from H_2_O and CO_2_, demonstrating the potential for sustainable energy solutions.^[^ ^11^, ^12^ ^]^

The thermochemical technology can be conceptualized as a chemical heat engine cycle mediated by metal oxides (MO_x_). Analogous to a traditional heat engine that operates between two thermal reservoirs to convert heat into work, the two‐step thermochemical cycle is envisioned as its chemical counterpart, where thermal energy is directly transformed into chemical energy, specifically into hydrogen. As described in Equations (1) and (2), the chemical heat engine with a two‐step thermochemical water splitting cycle is characterized by the thermal reduction reaction that releases oxygen from metal oxide at the temperature *T*_TR_, and the water splitting reaction to produce hydrogen and incorporate oxygen into metal oxide at the temperature *T*_WS_.

(1) $$\begin{array}{ccc} \mathrm{Thermal}\;\mathrm{reduction}:\;{\mathrm{MO}}_{y1}\to {\mathrm{MO}}_{y2} & + & \left(\left(y_{1}-y_{2}\right)/2\right)O_{2}\;\\ & & \left(T_{\mathrm{TR}\;}\geq 1100^{\circ }C\right) \end{array}$$

(2) $$\begin{array}{ccc} \mathrm{Water}\;\mathrm{splitting}:\;{\mathrm{MO}}_{y2}+\left(y_{1}-y_{2}\right)H_{2}O & \to & {\mathrm{MO}}_{y1}+\left(y_{1}-y_{2}\right)H_{2}\\ & & \left(T_{\mathrm{WS}}\geq 600^{\circ }C\right) \end{array}$$

**Figure 1:**
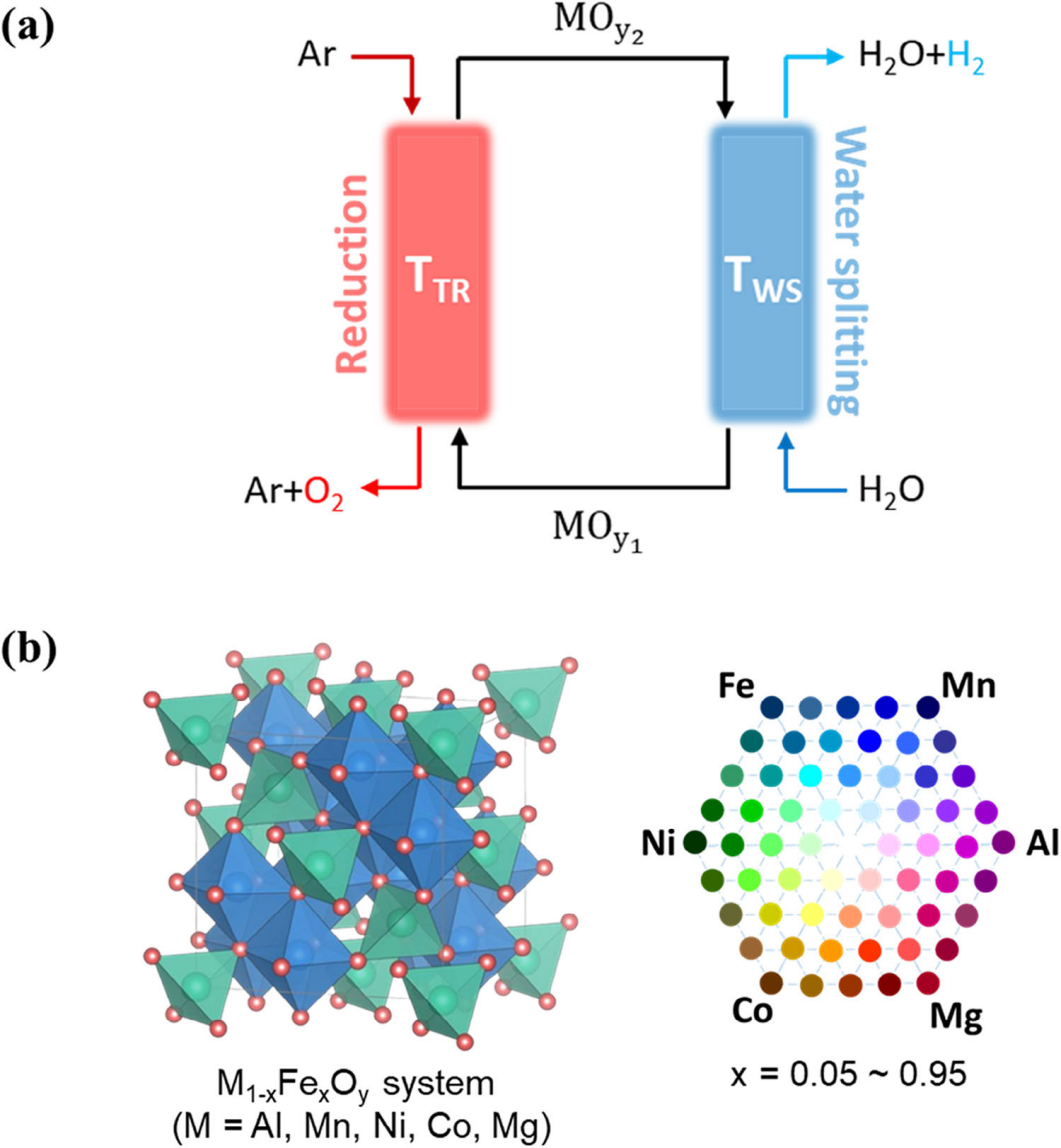
a) Schematic representation of the two‐step thermochemical H_2_ production cycle, and b) constituent cations and compositions of the multi‐cation ferrite system M_1‐x_Fe_x_O_y_ (M═Mn, Al, Mg, Co, Ni) considered for high‐throughput thermodynamic screening.

Research on the cycle has not only focused on the system modeling,^[^ ^14^, ^15^, ^16^ ^]^ novel reactor design,^[^ ^17^, ^18^, ^19^, ^20^, ^21^ ^]^ and efficient heat exchangers^[^ ^22^, ^23^, ^24^ ^]^ but also on searching novel oxides to enhance the cycle efficiency,^[^ ^25^, ^26^, ^27^, ^28^, ^29^, ^30^, ^31^, ^32^, ^33^, ^34^ ^]^ since the oxide is the essential component of the redox reactions which are fundamental to the cycle's operation (**Figure** **1**a). To ensure the spontaneity of each reaction, these oxides should satisfy the negative Gibbs free energy changes (Δ*G*_rxn_ < 0) under the given cycle conditions defined by specific temperature and gas partial pressure. The thermal reduction typically occurs at relatively higher temperature (*T*_TR_ ≥ 1100 °C) and low partial pressure of oxygen (*P*(O_2_) < 10^−3^ atm), whereas water splitting is favored at lower temperature (*T*_WS_ ≥ 600 °C).^[^ ^35^, ^36^ ^]^ Within the spontaneous process range, the degree of oxygen loss and gain by metal oxide, termed as the oxygen capacity (ΔO ≡ y_1_ − y_2_), is inherently dependent on the thermodynamic properties of the oxide material (that is, enthalpy and entropy).^[^ ^25^, ^37^, ^38^, ^39^, ^40^ ^]^

To achieve a high oxygen capacity, a variety of oxides that can be broadly categorized into ceria (CeO_2_), perovskite oxides, and ferrites have been developed. Among these, CeO_2_ has been extensively researched as a benchmark oxide in cycles involving high reduction temperature (*T*_TR_ ≥ 1500 °C) and significant temperature swings between the redox reactions (Δ*T*_redox_ ≡ *T*_TR_ − *T*_WS_ ≥ 500 °C).^[^ ^12^ ^]^ However, the high *T*_TR_ and large Δ*T*_redox_ result in low system durability and high sensible heat losses. State‐of‐the‐art perovskite oxides, such as the CaMnO_3_, BaCeO_3_, and LaSrMnO_3_ family, have addressed these challenges by reducing the reduction enthalpy (Δ*H*_O_) while obtaining an appropriate entropy (Δ*S*_O_) range, exhibiting higher ΔO or H_2_ yields compared to the CeO_2_ at *T*_TR_ = 1350 °C and Δ*T*_redox_ = 200–300 °C.^[^ ^27^, ^29^, ^30^, ^31^, ^41^, ^42^ ^]^ Despite their success under the alleviated reaction conditions, perovskite oxides exhibit not only poor H_2_O‐to‐H_2_ conversion efficiencies, resulting in the use of excess steam input, but also higher heat capacities (*C*_p_) than CeO_2_, necessitating larger energy for heating the materials. The *C*_p_ are ≈80 J (mol‐oxide∙K)^−1^ for CeO_2_,^[^ ^43^ ^]^ 110 J (mol‐oxide∙K)^−1^ for CaMnO_3_,^[^ ^44^ ^]^ 125 J (mol‐oxide∙K)^−1^ for BaCeO_3_,^[^ ^45^ ^]^ and 150 J (mol‐oxide∙K)^−1^ for LaSrMnO_3_ (LSM) family.^[^ ^31^, ^46^ ^]^ These factors could potentially reduce the overall system efficiency, even compared to undoped CeO_2_.^[^ ^46^ ^]^

While ferrite materials have traditionally been studied with Fe‐rich compositions,^[^ ^47^, ^48^, ^49^ ^]^ recently, materials with Fe‐poor compositions (M_1‐x_Fe_x_O_y_, M═Ni, Co, Mg, x< 0.5) have gained attention for their superior thermodynamic properties.^[^ ^25^, ^28^, ^39^ ^]^ Previous research have reported optimized compositions for Ni─Fe─O, Co─Fe─O, and Mg─Fe─O systems, demonstrating higher H_2_ yields and reasonable H_2_O‐to‐H_2_ conversion compared to the CeO_2_ and LaSrMnO_3_ family under the redox conditions of *T*_TR_ = 1100–1500 °C and *T*_WS_ = 800 °C.^[^ ^25^, ^39^ ^]^ Notably, among the Fe‐poor ferrites, those incorporating multiple cations, such as (MgNiCoFe)O_y_ (Poly‐Cation Oxide, PCO), stand out for their high H_2_ yields and the H_2_O‐to‐H_2_ conversion, which are primarily attributed to Fe being the main active cation with a significantly large redox contribution.^[^ ^28^ ^]^ Alongside the redox performance, the *C*_p_ of the ferrite system is less than 80 J (mol‐oxide·K)^−1^, similar to or slightly lower than that of CeO_2_, potentially offering high cycle efficiency. Building on these advantages, investigations into substituting other cations than Mg, Co, and Ni or exploring other active cations besides Fe could provide significant opportunities to advance the development of redox materials and to enhance the efficiency of the thermochemical cycles.

In this study, we report the discovery of the novel multi‐cation oxides system (MgMnCo)_1‐x_Fe_x_O_y_ (MMCFs, x < 0.5) with superior thermochemical water splitting performance under mitigated redox conditions; *T*_TR_ = 1300 °C and *T*_WS_ = 1100 °C. We employed high‐throughput thermodynamic screening to identify the novel ferrite system using CALPHAD simulations, and explicitly calculated the cycle efficiency of the promising material system. As shown in Figure 1b, we conducted a total of 1550 cycle screenings for the Fe‐based oxides system (M_1‐x_Fe_x_O_y_, M═Mn, Al, Mg, Co, Ni, 0.05< x< 0.95), and discovered the most promising system MMCFs among the candidates. The redox performance of MMCFs was experimentally validated, confirming its outstanding H_2_ yields and H_2_O‐to‐H_2_ conversion. A comprehensive analysis of fundamental thermodynamic parameters showed distinctively favorable Δ*H*_O_, Δ*S*_O_, and *C*_p_ of MMCFs compared to other previous materials. The electronic structure analysis using X‐ray absorption spectroscopy revealed that such favorable thermodynamic characteristics of MMCFs are attributed to the dual activation of Fe and Mn cations during redox cycles–a feature discovered for the first time in any ferrite cycles. This finding is further supported by the thermodynamic calculations of cation distribution, which reveal that Fe and Mn, in particular, undergo redistribution accompanied by changes in oxidation states.

## 2 Results and Discussion

### 2.1 Thermodynamic Simulation for the Oxygen Capacity Using CALPHAD

**Figure 2:**
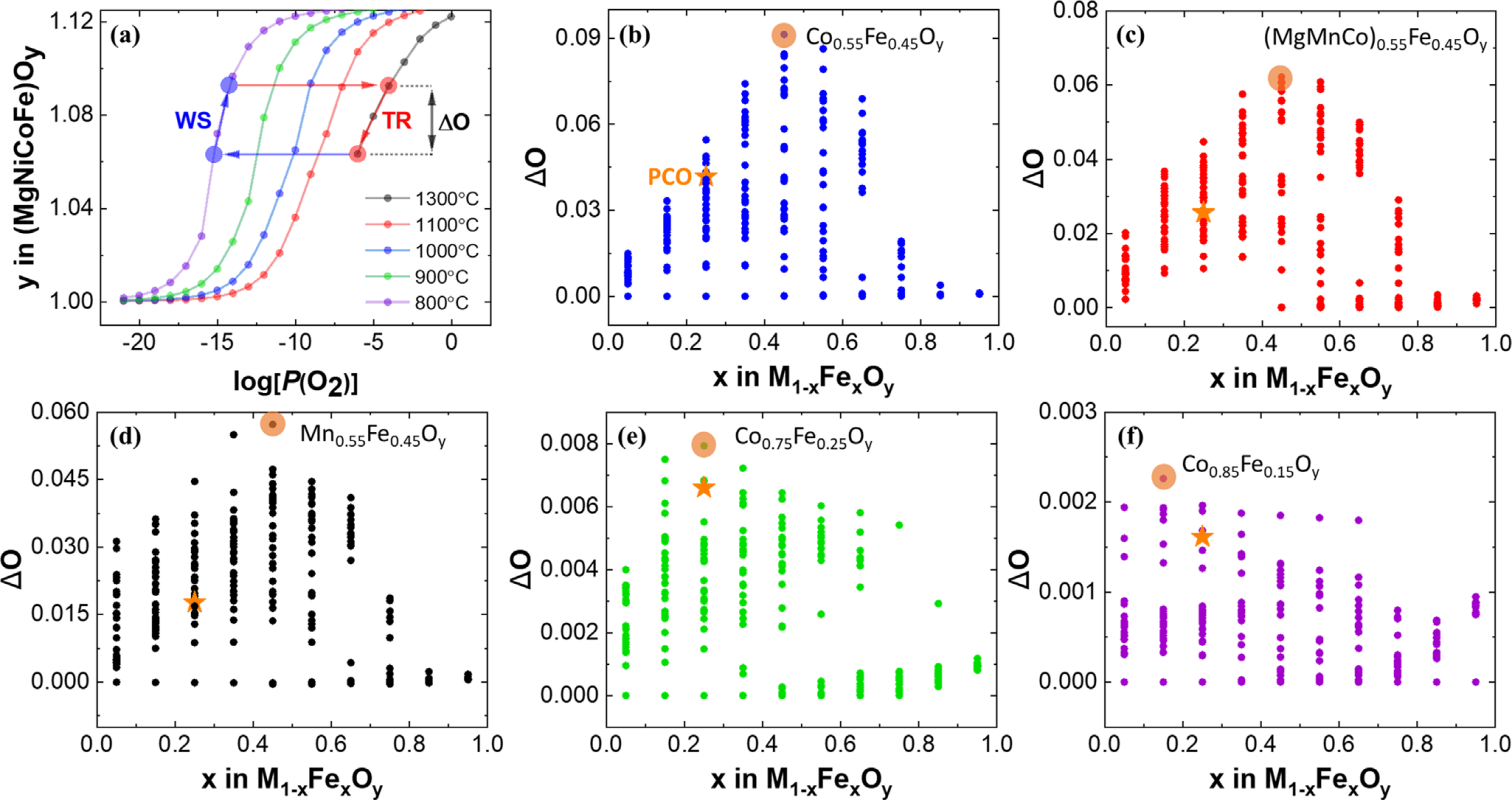
Thermodynamic predictions of oxygen capacity for multi‐cation ferrites under various temperature conditions. a) Schematic illustration showing how the theoretical oxygen capacity is calculated using a benchmark PCO composition. b–f) Oxygen capacity at specific temperature conditions with identical *P*(O_2_) = 3∙10^−6^ atm at the thermal reduction step: (b) *T*_TR_ = 1300 °C and *T*_WS_ = 800 °C, (c) *T*_TR_ = 1300 °C and *T*_WS_ = 1100 °C, (d) *T*_TR_ = 1300 °C and *T*_WS_ = 1300 °C, (e) *T*_TR_ = 1100 °C and *T*_WS_ = 900 °C, and (f) *T*_TR_ = 1000 °C and *T*_WS_ = 800 °C. The orange star represents the theoretical oxygen capacity of the poly‐cation oxide (PCO). The complete data set is summarized in Tables S4 and S5 (Supporting Information).

**Figure** **2**a presents a schematic illustration of how the theoretical oxygen capacity (ΔO) is calculated for each cycle condition, using a benchmark PCO composition as an example. Each data point represents the oxygen content of the PCO under a specific (*T*, *P*(O_2_)) condition calculated by CALPHAD (See details in Supporting Information). During the TR process, the oxygen content decreases as oxygen gas evolves at high temperature and reaches thermodynamic equilibrium. In contrast, during the WS process, the oxygen content increases as oxygen from H_2_O is incorporated at a lower temperature, again approaching equilibrium with the corresponding *P*(O_2_). Using this methodology, ΔO can be systematically calculated for numerous combinations and compositions of multi‐cation ferrites.^[^ ^50^, ^51^, ^52^ ^]^

The ΔO of the M_1‐x_Fe_x_O_y_ system (M═Mn, Al, Mg, Co, Ni) is shown in Figure 2b–f. The temperature conditions (*T*_TR_, *T*_WS_) are represented by distinct colors in five combinations: blue dot for (1300, 1300 °C), red dot for (1300, 1100 °C), black dot for (1300, 800 °C), green dot for (1100, 900 °C), and purple dot for (1000, 800 °C) with an identical *P*(O_2_) = 3∙10^−6^ atm at the thermal reduction step. The complete data set is summarized in Tables S4 and S5 (Supporting Information). The ΔO for a benchmark PCO at each temperature condition is differentiated by an orange star. At the conditions of *T*_TR_ = 1300 °C and *T*_WS_ = 800 °C, Co_0.55_Fe_0.45_O_y_ was calculated to be most promising with an approximate ΔO of 0.09, consistent with the previous study that reported an optimized composition of the ternary ferrite system by CALPHAD simulation (Figure 2b).^[^ ^25^, ^53^ ^]^ As Δ*T*_redox_ decreases to 200 °C and 0 °C at *T*_TR_ = 1300 °C, (MgMnCo)_0.55_Fe_0.45_O_y_ (MMCF45) and Mn_0.55_Fe_0.45_O_y_ were predicted as promising materials with ΔO values of 0.062 and 0.057, respectively, which are competitive values considering the theoretical ΔO of CeO_2_ is ≈0.015 with *T*_TR_ = 1300 °C, *T*_WS_ = 1100 °C, and *P*(O_2_) = 10^−6^ atm (Figure 2c,d). When Δ*T*_redox_ remains at 200 °C, but *T*_TR_ decreases to 1100 °C and 1000 °C, respectively, ΔO decreases by an order of magnitude, with the predicted materials Co_0.75_Fe_0.25_O_y_ and Co_0.85_Fe_0.15_O_y_ showing the values of 0.008 and 0.002 (Figure 2e,f). Interestingly, while the PCO ranks mid‐range in ΔO at high reduction temperature (*T*_TR_ = 1300 °C), it ranks higher at lower reduction temperature (*T*_TR_ ≤ 1100 °C). This, along with the fact that promising materials are distinguished by different temperature sets, suggests that the relative thermodynamic merit of the same material can vary depending on the temperature conditions.

### 2.2 Experimental Validation for the Promising H_2_ Production Cycle

Among the predicted promising cycles, experimental validation was conducted for the MMCFs ((MgMnCo)_1‐x_Fe_x_O_y_, 0.25≤ x≤ 0.45) system under the conditions of *T*_TR_ = 1300 °C and *T*_WS_ = 1100 °C. Particular emphasis was placed on this relatively mild Δ*T*_redox_, as larger Δ*T*_redox_ have historically resulted in significant sensible heat losses. Accordingly, the MMCFs system was selected as a more practical operating material, offering a balance between redox activity and system‐level compatibility. We also acknowledge that if future reactor designs effectively mitigate heat losses even under large Δ*T*_redox_, material systems such as Co_0.55_Fe_0.45_O_y_ could emerge as promising candidates according to our thermodynamic predictions. Meanwhile, the present study focuses on the performance and characteristics of the MMCFs system.

**Figure 3:**
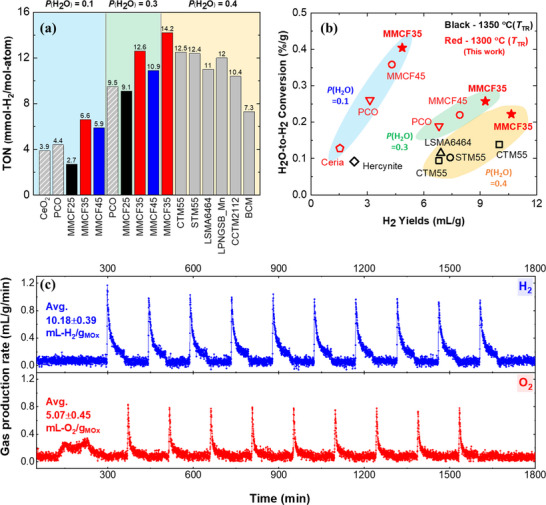
Two‐step thermochemical H_2_ production cycles for the predicted (MgMnCo)_1‐x_Fe_x_O_y_ material system. a) Turnover number (TON) at *T*_TR_ = 1300 °C for 1 h and *T*_WS_ = 1100 °C for 1 h, under varying steam partial pressure conditions, *P*(H_2_O) = 0.1 to 0.4 atm. b) H_2_ yields and average H_2_O‐to‐H_2_ conversion per cycle, normalized to the material quantity. Red points represent data experimentally in our reactor setup, and the black points represent literature values for other state‐of‐the‐art materials. The data in (a) and (b) for CTM55 (□),^[^ ^27^ ^]^ STM55 (○),^[^ ^41^ ^]^ LSMA6464 (△),^[^ ^42^ ^]^ Hercynite (◇),^[^ ^54^ ^]^ LPNGSB_Mn,^[^ ^30^ ^]^ CCTM2112,^[^ ^55^ ^]^ and BCM^[^ ^29^ ^]^ were sourced from the literature, while the other materials were evaluated in our setup. c) Performance stability of (MgMnCo)_0.65_Fe_0.35_O_y_‐ZrO_2_ composite over 10 cycles, with a composition ratio of (MgMnCo)_0.65_Fe_0.35_O_y_:ZrO_2_ = 30 wt.%:70 wt.%.

The reduction reaction was carried out at approximately *P*(O_2_) = 3∙10^−6^ atm for 1 hr, and the water splitting reaction was conducted at *P*(H_2_O) = 0.1, 0.3, and 0.4 atm for 1 hr. Three cycles were performed for each condition, and the average H_2_ yields from the second and third cycles were compared with those of CeO_2_ and PCO tested under the identical experimental conditions in our experimental setup, as well as with the experimental values for current state‐of‐the‐art materials reported in the literature (**Figure** **3**a).^[^ ^27^, ^29^, ^30^, ^41^, ^42^, ^54^ ^]^ To enable a fair comparison with different material systems without bias favoring lighter compositions or larger ion numbers in formula units, the H_2_ production per atom in the formula unit, represented by the turnover number (TON), was compared. The TON was calculated following the conventional method in the literature: TON ≡ (mmol‐H_2_/mol‐oxide) × (mol‐oxide/mol‐atom).^[^ ^55^ ^]^ For instance, CeO_2_ and Ca_2/3_Ce_1/3_Ti_1/3_Mn_2/3_O_3_ have a total of 3 and 5 mol of atoms per mol of formula unit, respectively.

At *P*(H_2_O) = 0.1 atm, MMCF35 and MMCF45 showed significant TON of 6.6 and 5.9 mmol‐H_2_/mol‐atom, respectively, compared to 3.9 mmol‐H_2_/mol‐atom for CeO_2_ and 4.4 mmol‐H_2_/mol‐atom for PCO. The TON observed for CeO_2_ corresponds to ≈83% of the theoretical capacity estimated from ΔO under the given temperature conditions (Figure S12, Supporting Information) As *P*(H_2_O) increased from 0.1 to 0.3 atm, MMCF35 and MMCF45 exhibited TON of 12.6 and 10.9 mmol‐H_2_/mol‐atom, respectively, followed by MMCF25 and PCO with similar TON of 9.1 and 9.5 mmol‐H_2_/mol‐atom, respectively. At *P*(H_2_O) = 0.4 atm, a relatively high steam supply regime where various perovskite oxides have been evaluated in literature, MMCF35 showed a TON of 14.2 mmol‐H_2_/mol‐atom. This demonstrated state‐of‐the‐art performance, showing exceptional H_2_ production per atom in the formula unit (Figure 3a).

Meanwhile, the H_2_ production per unit mass of oxides and the H_2_O‐to‐H_2_ conversion remain practical performance metrics in industrial applications, even considering the aforementioned bias. Figure 3b shows these mass‐based performance metrics. The H_2_ yields were normalized by the total amount of H_2_O supplied during the water‐splitting reaction to compare the H_2_O‐to‐H_2_ conversion per gram of oxide. The total amount of H_2_O input was calculated from the experimental conditions in each literature as follows: total H_2_O input = gas flow rate (mL min^−1^) × steam concentration (vol %) × water splitting reaction time (min). The MMCF35 excels in both H_2_ yields and H_2_O‐to‐H_2_ conversion across all conditions from *P*(H_2_O) = 0.1 to 0.4 atm compared to current state‐of‐the‐art perovskite oxides such as CTM55 (Figure 3b). It is noted that while CTM55 exhibited a similar TON to STM55 in Figure 3a, its use of a lighter element, Ca, at the A‐site of perovskite oxides instead of Sr makes it potentially attractive for mass‐based industrial applications. MMCF35 exhibited state‐of‐the‐art performance regardless of the constituent bias in Figure 3a,b. It is also noted that while the H_2_ yields of MMCF35 increase from 4.85 to 10.65 mL g^−1^ with increasing *P*(H_2_O) from 0.1 to 0.4 atm, the H_2_O‐to‐H_2_ conversion decreases from 0.4% to 0.22% g^−1^, necessitating an optimization process depending on the specific system‐level demand. Although the theoretical yields suggest that MMCF45 has a higher capacity of 20.15 mL g^−1^ compared to 14.85 mL g^−1^ for MMCF35 (Figure S5, Supporting Information), the experimental results showed the opposite trend, which is attributed to slower kinetics as the Fe concentration increased from 0.35 to 0.45. This aspect will be further discussed in the electronic structural analysis later.

The cyclability of MMCF35 was evaluated over 15 cycles at *P*(H_2_O) = 0.3 atm. The H_2_ yields gradually decreased until the fifth cycle due to sintering of ferrites, showing an average of 7.8 ± 0.47 mL g^−1^ excluding the first cycle (Figures S6 and S7, Supporting Information). To prevent the sintering effect, we synthesized an MMCF35: ZrO_2_ = 30 wt.%:70 wt.% composite using ZrO_2_, a commonly utilized stable support material (Figure S8, Supporting Information).^[^ ^25^, ^47^, ^49^ ^]^ The MMCF35/ZrO_2_ composite system effectively prevented sintering effect, demonstrating stable H_2_ production performance over 10 cycles while exhibiting excellent average yields of 10.18 ± 0.39 mL g^−1^‐ferrite (Figure 3c). The applicability of the present screening method was further validated through additional experiments on the Co_0.75_Fe_0.25_O_y_ predicted as promising for the milder temperature conditions of *T*_TR_ = 1100 °C and *T*_WS_ = 900 °C (Figure S9, Supporting Information). This material also achieved an H_2_ yield of 1.2 mL g^−1^, approximately four times higher than 0.32 mL g^−1^ of the benchmark PCO.

### 2.3 Crystal and Electronic Structural Analysis of the MMCFs System

**Figure 4:**
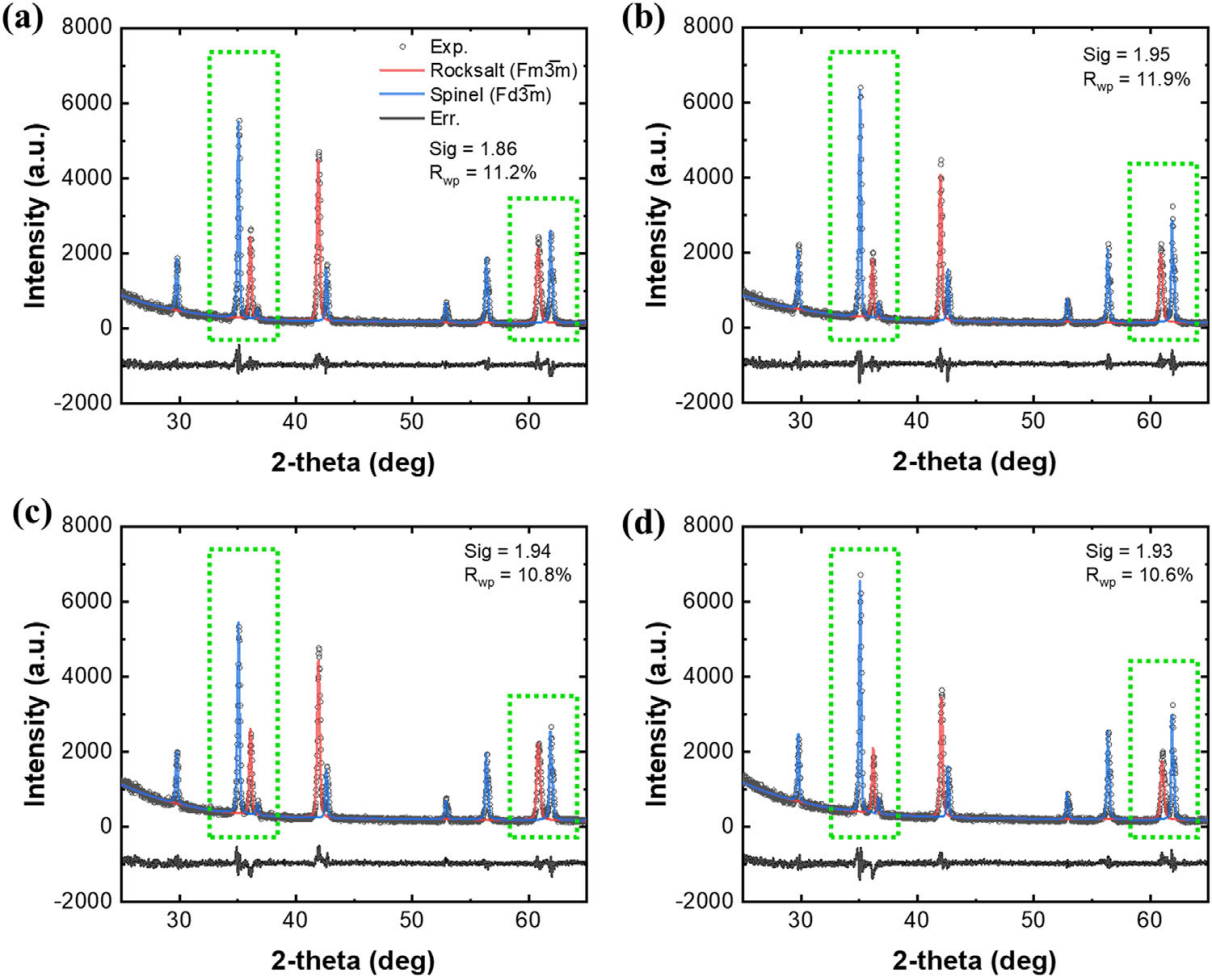
XRD patterns of MMCF35. a,b) XRD patterns and corresponding Rietveld refined patterns quenched after thermal reduction at *T*_TR_ = 1300 °C and water splitting at *T*_WS_ = 1100 °C and *P*(H_2_O) = 0.1 atm, respectively. c,d) the XRD patterns at *T*_TR_ = 1300 °C and *T*_WS_ = 1100 °C and *P*(H_2_O) = 0.3 atm, respectively.

**Figure** **4** shows the crystal structural evolution of MMCF35 during redox cycles under varying H_2_O partial pressures, as determined by XRD and Rietveld refinement.^[^ ^56^, ^57^, ^58^ ^]^ Upon cycling between thermal reduction (*T*_TR_ = 1300 °C) and water splitting (*T*_WS_ = 1100 °C), a phase transformation is observed between the rocksalt and spinel structures. At a lower *P*(H_2_O) of 0.1 atm (Figure 4a,b), the spinel phase fraction increases from 52.0 wt.% to 56.0 wt.%, while the rocksalt fraction decreases from 48.0 wt.% to 44.0 wt.%, corresponding to a phase swing of 4.0 wt.%. Under a higher *P*(H₂O) of 0.3 atm (Figure 4c,d), the spinel phase increases more significantly from 52.4 wt.% to 61.7 wt.%, and the rocksalt phase correspondingly decreases from 47.6 wt.% to 38.3 wt.%, yielding a phase swing of 9.3 wt.%. This increased phase transformation at elevated *P*(H_2_O) suggests enhanced redox activity under more oxidative environments within the current cycle duration. This trend in phase transformation is also consistent with the calculated phase diagram (Figure S10, Supporting Information).

**Figure 5:**
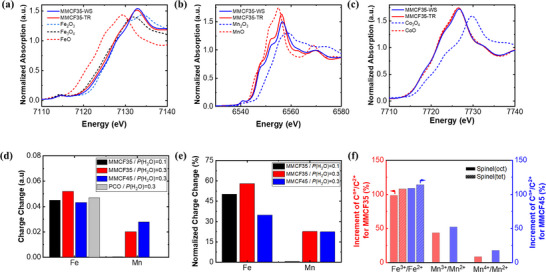
Electronic structural analysis of (MgMnCo)_0.65_Fe_0.35_O_y_ for *T*_TR_ = 1300 °C, and *T*_WS_ = 1100 °C for 1 h and *P*(H_2_O) = 0.3 atm cycle. a–c) Normalized X‐ray absorption spectra at the Fe K‐edge, Mn K‐edge, and Co K‐edge, respectively. d,e) Corresponding charge change and normalized charge change of each cation. See the detailed absorption spectra in Figure S5 (Supporting Information). f) Increment of C^n+^/C^2+^ (where n ≥ 3, C═Fe, Co, Mn, Mg) after water splitting compared to those after thermal reduction for MMCF35 and MMCF45, calculated using the CALPHAD simulation. See the detailed cation distribution in Tables S2 and S3 (Supporting Information).

To reveal the contributing redox‐active cations in the MMCFs system, the electronic structure was analyzed using X‐ray absorption spectroscopy.^[^ ^59^ ^]^ The oxidation state changes of Fe, Mn, and Co cations in MMCF35 were analyzed for *T*_TR_ = 1300 °C, and *T*_WS_ = 1100 °C, and *P*(H_2_O) = 0.3 atm (**Figure** **5**a–c). Fe exhibits an oxidation state of +2.635 at the thermal reduction and +2.784 at the water splitting, resulting in a change of +0.149, while Mn shows a change of +0.094, and Co shows a negligible change. Based on the oxidation state changes, the charge change considering the stoichiometry of each cation is calculated for comparison with other materials and under various gas partial pressures; charge change = oxidation state change × stoichiometry of each cation. PCO shows that only Fe exhibits redox‐active property among several cations, which is consistent with the previous study.^[^ ^28^ ^]^ On the other hand, MMCFs show dual active characteristics of Mn and Fe, contributing to the large H_2_ yields (Figure 5d; Figure S4, Supporting Information). For more detailed analysis, the charge change was normalized by the theoretical maximum charge change dictated by theoretical ΔO. For instance, a ΔO of 0.045 in MMCF35 indicates the maximum charge change of 0.09, as loss or gain of one oxygen atom can alter two electron charges. As shown in Figure 5e, at *P*(H_2_O) = 0.1 atm, only Fe contributes to the redox reaction in MMCF35, accounting for ≈47% of the theoretical capacity. As *P*(H_2_O) increases to 0.3 atm, leading to a more oxidative environment, not only does the contribution of Fe increase to ≈58%, but Mn also starts to contribute, accounting for 22.6% of the capacity, thereby resulting in dual active characteristics.

As discussed in an earlier section, theoretical predictions indicate that MMCF45 should achieve a higher hydrogen yield due to its greater ΔO. However, experimental results reveal the opposite trend, with MMCF35 achieving superior yields. This discrepancy is presumed to stem from kinetic factors in the redox behavior of the materials, rather than thermodynamic potential alone. In specific, while MMCF45 shows a similar normalized charge change from Mn (22.4%) compared to MMCF35, it shows a lower change from Fe (34.8%) (Figure 5e). These findings suggest that increasing the Fe concentration from 0.35 to 0.45 in MMCFs could enhance the oxygen capacity but simultaneously impede the kinetics of the redox reaction, thereby reducing the effectiveness of Fe as a redox‐active cation. This is further clarified in the subsequent analysis of cation redistribution between the phases.

The redox mechanism underlying the MMCFs system was further explored through thermodynamic calculations of cation distribution utilizing the CALPHAD. This system comprises two solid solution phases, rocksalt and spinel. The spinel phase contains octahedral and tetrahedral sites accommodating various oxidation states of Mg, Mn, Co, and Fe, as detailed in Tables S2 and S3 (Supporting Information). Phase transformation from the rocksalt to the spinel phase occurs as thermally reduced MMCFs undergo the water splitting reaction, accompanied by cation redistribution. The distribution ratio of each high valence cation to the low valence counterpart (*C* ^n+^/*C* ^2+^, where *n* ≥ 3, *C*═Fe, Co, Mn) was quantified based on its variation from thermal reduction to water splitting at each site. Figure 5f shows the increment percentage of *C* ^n+^/*C* ^2+^ in the spinel phase after water splitting. Notable increments of the high valence cations are observed, particularly shown as a significant increase in Fe^3+^/Fe^2+^, followed by an increase in Mn^3+^/Mn^2+^ and Mn^4+^/Mn^2+^. High valence states of Co and Mg are found to be either thermodynamically absent or present in negligible amounts. These characteristics of MMCFs system consistently support the results from XANES analysis: dual activation of Fe and Mn, and inert behavior of Mg and Co.

Comparing the increments for MMCF35 and MMCF45, MMCF35 exhibits increases of 98.2% and 107.6% in Fe^3+^/Fe^2+^ at the octahedral and tetrahedral sites, respectively, in the spinel phase, whereas MMCF45 shows relatively larger increments of 108.5% and 113.9%, respectively. Similarly, MMCF45 displays relatively larger increments of 51.8% and 17.4% in Mn^3+^/Mn^2+^ and Mn^4+^/Mn^2+^, respectively, at the octahedral site, compared to 43.3% and 8.2% for MMCF35 (Figure 5f). However, considering the higher Fe and lower Mn content of MMCF45 than MMCF35, the larger theoretical ΔO of MMCF45 presumably originates from the larger theoretical redox swing between Fe^3+^ and Fe^2+^. On the other hand, a phase transformation from the rocksalt phase to the spinel phase occurs during the water splitting reaction, resulting in the formation of an Fe‐rich spinel phase in the MMCF45 (Figure S11, Supporting Information). This suggests that the redox activity of Fe could be strongly associated with the phase transformation process during the redox reactions, which is, in general, kinetically slow. Thus, it is plausible that such slow redox kinetics of Fe explains the experimentally observed lower redox performance of MMCF45 than MMCF35, contrary to the theoretical thermodynamic prediction. Further investigations are imperative to elucidate a more detailed mechanism, integrating both thermodynamic and kinetic perspectives to fully understand the redox behavior and phase dynamics in the MMCFs system.

### 2.4 Thermodynamic Characteristics of MMCF35 System

To derive the theoretical cycle efficiency, the thermodynamic characteristics of MMCF35 were analyzed through CALPHAD simulation. The solar‐to‐fuel efficiency, which quantifies the H_2_ energy obtained relative to the thermal energy absorbed by the material, is defined by Equation (3), where $HHV_{H_{2}}$ and $n_{H_{2}}$ represent the higher heating value of H_2_ (285.8 kJ mol‐H_2_^−1^) and the H_2_ yield, respectively, and, *Q_thermal_* is the thermal energy input.

(3) $$\begin{array}{c} \;\eta_{solar-to-fuel}=\frac{HHV_{H_{2}}\cdot n_{H_{2}}}{Q_{thermal}} \end{array}$$

As shown in Equations (4) and (5), *Q_thermal_* consists of the energy required to heat the oxide and the change in enthalpy from *T_WS_* to *T_TR_*, where η_*abs*_ is the solar absorption efficiency, Δ*H_O_* is the enthalpy of reduction at a given oxygen non‐stoichiometry (δ) of metal oxide, and δ_*TR*_ and δ_*WS*_ represent δ under thermal reduction and water splitting conditions, respectively. It should be noted that Δ*H_O_* can be varied with δ.

(4) $$Q_{thermal}=\frac{1}{\eta_{abs}}\left(\int_{T_{WS}}^{T_{TR}}C_{P}dT+\Delta H_{TR}\cdot \delta n_{ox}\right)$$

(5) $$\begin{array}{c} \Delta \;H_{TR}=\frac{\int_{\delta_{WS}}^{\delta_{TR}}\Delta H_{O}d\delta }{\delta_{TR}-\delta_{WS}} \end{array}$$

The η_*abs*_ for a blackbody receiver was calculated consistently with previous literature using Equation (6),^[^ ^31^ ^]^ where σ represents the Stefan–Boltzmann constant, *I* is the direct solar irradiation is assumed to be 1000 W m^−2^, and *C* is the solar flux concentration ratio is assumed to be 5000. These values are widely adopted in the literature as benchmark conditions for terrestrial solar concentrator systems.^[^ ^31^, ^37^, ^60^ ^]^ Under these conditions, η_*abs*_ is calculated to be ≈0.93 at *T*_TR_ = 1300 °C.

(6) $$\begin{array}{c} \;\eta_{abs}=1-\frac{\sigma T^{4}}{IC} \end{array}$$

**Figure 6:**
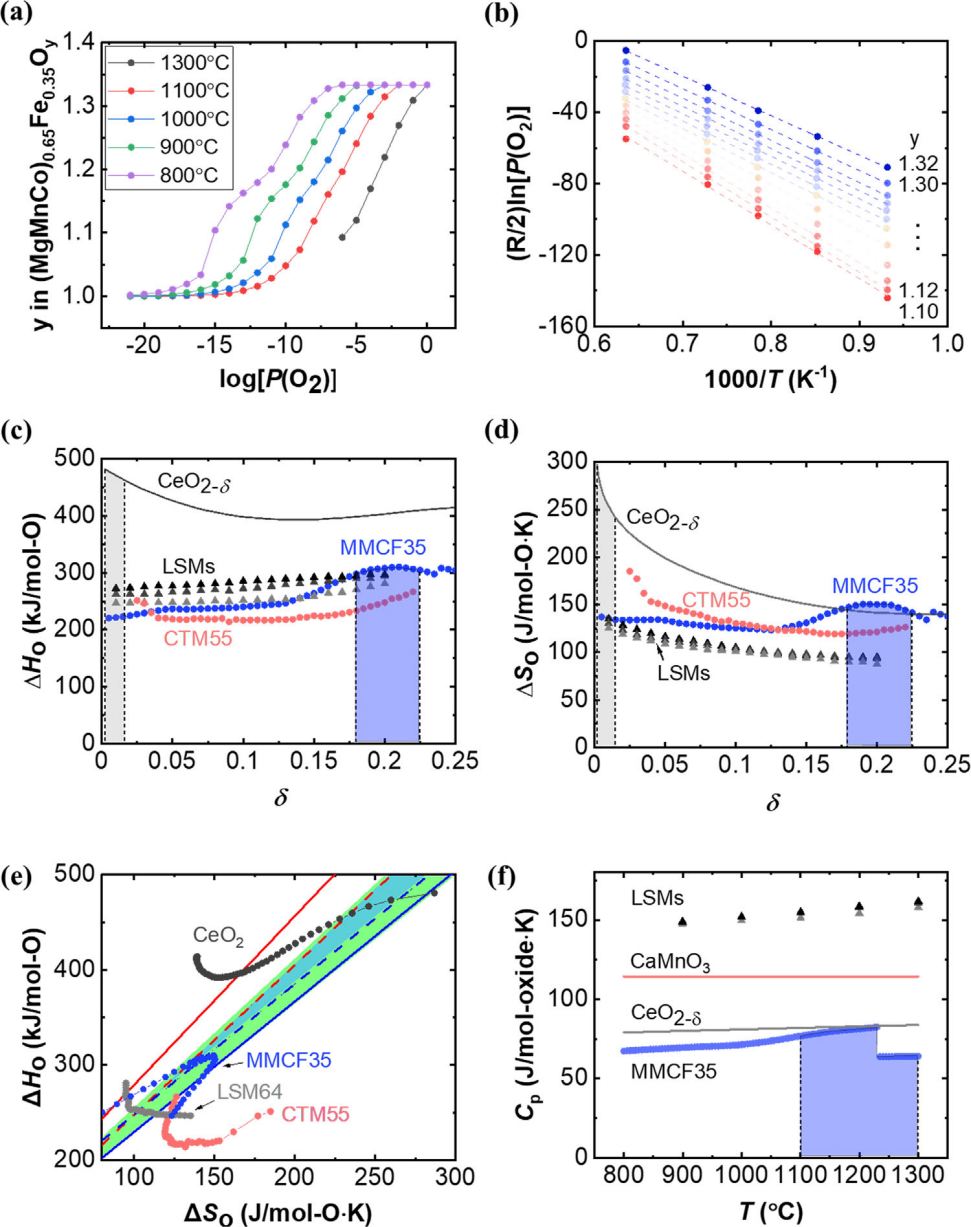
Thermodynamic characteristics of (MgMnCo)_0.65_Fe_0.35_O_y_ compared to other benchmarked materials. a) CALPHAD‐simulated oxygen content (y) of MMCF35 as a function of *P*(O_2_). b) van't Hoff plot derived from the simulated oxygen content of MMCF35 c) Standard enthalpy of reduction of MMCF35 and other benchmark oxides. d) Standard entropy of reduction for the same set of materials. Blue and gray shaded regions indicate *δ* ranges for MMCF35 and CeO_2_, respectively. The thermodynamic data for CeO_2_, La_1‐x_Sr_x_MnO_3_, and CaMnO_3_ were obtained from the literature.^[^ ^27^, ^31^, ^41^, ^44^, ^61^ ^]^ e) Thermodynamically favorable region for redox reactions. The red solid line and dashed line indicate the region where Δ*G* is zero for the thermal reduction at *T*_TR_ = 1500 °C and *T*_TR_ = 1300 °C at *P*(O_2_) = 10^−6^ atm, respectively. Blue solid and dashed lines indicate the region where the Δ*G* is zero for the water splitting at H_2_O:H_2_ = 1000:1 and 200:1 at *T*_WS_ = 1100 °C, respectively. The green highlighted region denotes the thermodynamically favorable area under a moderate H_2_ background (H_2_O:H_2_ = 1000:1) in the *T*_TR_ = 1300 °C and *T*_WS_ = 1100 °C cycle, while the blue region denotes the high H_2_ background area (H_2_O:H_2_ = 200:1). f) Temperature dependence of the heat capacity of the materials.

The fundamental thermodynamic parameters of an oxide material, the enthalpy (Δ*H_O_*) and entropy (Δ*S_O_*) of reduction can be extracted for a given oxygen non‐stoichiometry δ using the van't Hoff method as shown in Equation (7).^[^ ^27^, ^31^ ^]^ To calculate the δ for MMCF35 based on temperature and oxygen partial pressure, the CALPHAD simulation was employed to assess the material's oxygen content across the various (*T*, *P*(O_2_)) conditions (**Figure** **6**a). It was found that the oxygen content converges to 1.33 at all temperatures when log(*P*(O_2_)) = 0, resulting in the chemical formula of the MMCF35 as (MgMnCo)_0.65_Fe_0.35_O_1.33‐_ *_δ_*. Figure 6b shows the extracted equilibrium conditions for constant δ values based on the simulation results in Figure 6a. Linear regression of these points allows the calculation of Δ*H_O_* and Δ*S_O_* from the slope and y‐intercept, respectively, in accordance with Equation (7).

(7) $$\begin{array}{c} \frac{1}{2}\;{\left.\ln P\left(O_{2}\right)\right|}_{\delta =const}=\frac{\Delta H_{O}}{RT}\;-\frac{\Delta S_{O}}{R} \end{array}$$

To compare the extracted Δ*H_O_* and Δ*S_O_* of MMCF35 with those of other state‐of‐the‐art materials previously reported, the δ values for MMCF35 were calculated under the given thermal reduction and water splitting conditions as δ_*TR*_ = 0.224 and δ_*WS*_ = 0.1792. This specific range was highlighted in blue in Figure 6c,d. MMCF35 features a moderate Δ*H_O_* range from 295.6 to 310.2 kJ mol─O^−1^ and a large Δ*S_O_* range from 141.5 to 150.4 J (mol─O∙K)^−1^. Direct comparisons with other state‐of‐the‐art perovskite oxides are challenging due to their undefined δ values under the same conditions (*T*_TR_, *T*_WS_); however, within the cycle range of MMCF35, it presents the most promising Δ*S_O_* between 0.1792< δ< 0.224. The thermodynamic properties of CeO_2_ were compared as a benchmarked material. At *T*_TR_ = 1300 °C, *P*(O_2_) = 10^−6^ atm and *T*_WS_ = 1100 °C, CeO_2_ is characterized by δ_*TR*_ = 0.0145 and δ_*WS*_ = 0.00135, represented in a gray‐colored region (Figure 6c,d; Figure S12, Supporting Information). Within the region, it exhibits significant values of Δ*H_O_* and Δ*S_O_*, ranging from 460 to 480 kJ mol─O^−1^ and 240 to 297 J (mol─O∙K)^−1^, respectively. The notable Δ*S_O_* has been considered as a beneficial thermodynamic feature; however, its considerably high Δ*H_O_* compared to other materials requires a high thermal energy input for reducing CeO_2_, potentially decreasing the cycle efficiency.

To evaluate the thermodynamic robustness of materials, we can define the thermodynamically spontaneous region for both thermal reduction and water splitting reactions by comprehensively considering the Δ*H_O_* and Δ*S_O_* of the materials that satisfy negative Gibbs free energy changes. As shown in Figure 6e, the favorable region defined by the cycle conditions of reduction (*T*_TR_ = 1300 °C, *P*(O_2_) = 10^−6^ atm) and water splitting (*T*_WS_ = 1100 °C, H_2_O:H_2_ = 1000:1) is highlighted in green. This condition is termed as a moderate H_2_O‐to‐H_2_ conversion scenario, where H_2_O:H_2_ denotes the molar ratio of H_2_O to background H_2_. The reactions within this area are spontaneous if the Δ*H_O_* and Δ*S_O_* of the materials fall within this region. In high H_2_O‐to‐H_2_ conversion scenarios, an increase in the background H_2_ ratio can narrow the spontaneous region, as depicted by the blue highlighted area defined by the cycle conditions of reduction (*T*_TR_ = 1300 °C, *P*(O_2_) = 10^−6^ atm) and water splitting (*T*_WS_ = 1100 °C, H_2_O:H_2_ = 200:1).

The thermodynamic characteristics of several materials, including state‐of‐the‐art perovskite oxides and MMCF35, have been analyzed by plotting their Δ*H_O_* and Δ*S_O_* at fine intervals (δ = 0.005). Under the green highlighted area (moderate H_2_O‐to‐H_2_ conversion scenario), MMCF35 and LSM64 show promising characteristics due to their numerous points, while CeO_2_ has fewer points, and CTM55 hardly joins this area. With an increase in background H_2_ (the blue area), the spontaneity of MMCF35 and CeO_2_ partially decreases, whereas LSM64 and CTM55 are completely excluded from this area (Figure S13, Supporting Information). The analysis reveals that the LSM64 and CTM55 families, which demonstrate notable spontaneous properties for the thermal reduction at temperatures lower than those required for CeO_2_, show a pronounced sensitivity to the background H_2_ level. The sensitivity might require a stringent control strategy, such as high partial pressure of input H_2_O or configuring an additional pump‐out system for rapid H_2_ swapping. It is noteworthy that MMCF35 demonstrates robust thermodynamic properties even in the blue area, potentially alleviating operational challenges of the CeO_2_ and perovskite systems, such as the high *T*_TR_ and large Δ*T*_redox_, as well as the sensitivity to background H_2_.

The calculated *C*_p_ of MMCF35 was compared with other benchmark materials (Figure 6f). The *C*_p_ of MMCF35 remains below 80 J (mol‐oxide∙K)^−1^ across the temperature range of 800 to 1300 °C, while LSM perovskite family exhibits a higher *C*_p_, ≈150 J (mol‐oxide∙K)^−1^, and CaMnO_3_ and CeO_2_ are ≈125 and 80 J (mol‐oxide∙K)^−1^, respectively. The abrupt decrease in *C*_p_ is attributed to the phase transformation ≈1230 °C (Figure S10, Supporting Information). The lower *C*_p_ of MMCF35 offers an advantage in reducing the energy required to heat the oxide from *T*_WS_ to *T*_TR_. Incorporating this into Equation (4) allows the *Q_thermal_* to be calculated under the cycle condition of *T*_TR_ = 1300 °C and *T*_WS_ = 1100 °C. Considering the theoretical H_2_ yield relative to the *Q_thermal_*, the theoretical cycle efficiency is calculated using Equation (3). The theoretical H_2_ yield for each material is obtained from its theoretical ΔO under the cycle condition. The calculated theoretical efficiencies are 43.6% for MMCF35 and 17.4% for CeO_2_. Given that the experimental H_2_ yields of MMCF35 are 71.7% of its theoretical capacity (Figure 6a; Figure S5, Supporting Information), the experimental efficiency is estimated to be ≈31.3%. Since the current efficiency estimation only involved the material‐related parameters, future system‐level analyses should consider auxiliary energy terms such as heat recuperation and steam heating.

## 3 Conclusion

In conclusion, we discovered the (MgMnCo)_1‐x_Fe_x_O_y_ system as a novel and highly efficient redox material for thermochemical hydrogen production cycles by employing a high‐throughput screening approach utilizing the CALPHAD methodology. Through the systematic and rigorous screening of 310 ferrite compositions (M_1‐x_Fe_x_O_y_, M═Mn, Al, Mg, Co, Ni, 0.05 ≤ x ≤ 0.95) across five sets of (*T*_TR_, *T*_WS_) conditions (Tables S4 and S5, Supporting Information), MMCFs system found to be most promising at the *T*_TR_ = 1300 °C and *T*_WS_ = 1100 °C cycle condition. Then it was experimentally demonstrated that a specific composition of MMCF35 achieves state‐of‐the‐art performances in both H_2_ yield and H_2_O‐to‐H_2_ conversion, surpassing existing state‐of‐the‐art materials such as CeO_2_ and perovskite oxides. The thermodynamic analysis resulted in a material theoretical efficiency of 43.6% for MMCF35, while CeO_2_ showed a theoretical efficiency of 17.4% under the same conditions. The outstanding redox performance of MMCF35 was attributed to the thermodynamic and electronic characteristics. Thermodynamic analysis showed that MMCF35 exhibited the favorability even in high H_2_O‐to‐H_2_ conversion scenario, while also demonstrating the advantage of lower energy demand due to its relatively low *C*_p_< 80 J (mol‐oxide∙K)^−1^ compared to other materials. Electronic structure analysis revealed that the dual activation mechanism of Fe and Mn contributes to the excellent redox performance, supported by the thermodynamic analysis of cation distribution. Further validation experiment on Co_0.75_Fe_0.25_O_y_, predicted promising for the *T*_TR_ = 1100 °C, and *T*_WS_ = 900 °C condition, demonstrated H_2_ yields approximately fourfold higher than the current state‐of‐the‐art PCO at *T*_TR_ = 1100 °C, confirming the general applicability of our high‐throughput screening approach. It should be noted that, to the best of available knowledge, our study is the first to report a novel multi‐cation ferrite system which contains another redox‐active cation such as Mn alongside Fe. This finding could provide significant opportunities to explore novel redox materials with dual or multi‐cation activation for enhanced performance. Furthermore, the high‐throughput screening approach demonstrated here may find broad utility in diverse thermochemical processes, offering a systematic framework for designing and optimizing redox materials.

## Conflict of Interest

The authors declare no conflict of interest.

## Author Contributions

D.L. and J.N. contributed equally to this work. D.L. and J.N. performed conceptualization, data curation, formal analysis, investigation, methodology, validation, visualization, software, and wrote, reviewed, and edited the final manuscript. B.‐G.P. performed data curation, formal analysis, investigation, wrote, reviewed, and edited the final manuscript. H.K. performed data curation, formal analysis, investigation, software, wrote, reviewed, and edited the final manuscript. I.‐H.J. and H.J. performed conceptualization, funding acquisition, investigation, methodology, project administration, resources, supervision, wrote, reviewed, and edited the final manuscript.

## Supporting information



Supporting Information

## Data Availability

The data that support the findings of this study are available from the corresponding author upon reasonable request.
