# Supplementary material for: Discovery of Novel Ferrites for Thermochemical H2 Production Cycle via High‐Throughput Thermodynamic Screening
Source: Adv Sci (Weinh). 2025 Jun 25;12(35):e01846. doi: 10.1002/advs.202501846 (PMC12463064; doi:10.1002/advs.202501846)
Supplement: Supplementary file 1 — Supporting Information [file ADVS-12-e01846-s001.docx]

**Discovery of Novel Ferrites for Thermochemical H_2_ Production Cycle via High-throughput Thermodynamic Screening**
Dongkyu Lee,‡^1^ Joonhyun Nam,‡^2^ Byeong-Gyu Park,^3^ Hyemin Kim,^1^ In-Ho Jung,^2,4,^* and Hyungyu Jin^1,^*

*^1^ Department of Mechanical Engineering, Pohang University of Science and Technology (POSTECH), Pohang 37673, Republic of Korea*

*^2^ Department of Materials Science and Engineering, Seoul National University, Seoul 08826, Republic of Korea*

*^3^ Pohang Accelerator Laboratory (PAL), Pohang University of Science and Technology, Pohang 37673, Republic of Korea*

*^4^ Research Institute of Advanced Materials, Seoul National University, Seoul 08826, Republic of Korea*

‡: D. Lee and J. Nam contributed equally to this work

* Corresponding authors: hgjin@postech.ac.kr (H. Jin), in-ho.jung@snu.ac.kr (I-H. Jung)

**Experimental Method**

***CALPHAD simulation***

To screen material composition and process condition, CALPHAD simulations were conducted for theoretical thermodynamic analysis of the H_2_ production process. For the CALPHAD simulation, FactSage thermodynamic software was employed. The thermodynamic database for the Fe-Mn-Al-Mg-Co-Ni-O system − containing spinel, rocksalt, corundum and metal solid solution – was systematically constructed in FactSage using experimental phase diagram data and thermodynamic measurements [1–3]. Using FactSage, equilibrium composition, cation distribution and thermodynamic properties such as enthalpy, entropy, heat capacity and Gibbs energy were calculated based on initial materials composition, temperature and gas environment.

Thermal reduction and water splitting cycle simulations were conducted for the various ferrite compositions (M_1-x_Fe_x_O_y_, M = Mn, Al, Mg, Co, Ni) from binary to multi-component systems. Each cation within M was considered in an equimolar ratio, with *x* values from 0.05 to 0.95 in 0.1 increments, totaling 310 compositions (**Table. S4 – S5**). The temperature sets for the thermal reduction and water splitting reaction (*T*_TR_ − *T*_WS_) were chosen as 1300 − 1300, 1300 − 1100, 1300 − 800, 1100 − 900 and 1000 − 800 ℃ (5 temperature sets), yielding a total of 1,550 cycle sets. The higher *T*_TR_ of 1300 ℃ enables comparisons with established state-of-the-art oxides [4–7], while *T*_TR_ of 1100 °C and below explores potential applications at lower temperatures.

The simulation for thermal reduction and water splitting cycle involved a sequence of two equilibrium calculations. In the thermal reduction step (TR), 1 mole of cations with selected composition was equilibrated at *T*_TR_ under *P*(O_2_) = 3∙10^-6^ atm, resulting an oxide with low oxygen content. In the water splitting step (WS), the resulting oxide was reacted with 35.4 moles of H_2_O, reaching equilibrium among oxide with high oxygen content, H_2_ and the remaining H_2_O. The input amount of H_2_O was determined to a specific value where representative ferrites could achieve approximate equilibrium considering criteria of previous modeling (**Figure. S1**) [6,8]. The equilibrium calculations provided theoretical values for oxygen non-stoichiometry, heat capacity, cation distribution for each oxide.

***Sample synthesis***

(MgMnCo)_1-x_Fe_x_O_y_ (x = 0.25, 0.35, 0.45) and (MgNiCoFe)O_y_ were synthesized via the sol-gel method, consistent with protocols detailed in previous studies [4,9]. The starting chemical salts, including Mg(NO_3_)_2_∙6H_2_O (98% − 102%, Sigma-Aldrich), Mn(NO_3_)_2_∙4H_2_O (> 97%, Sigma-Aldrich), Co(NO_3_)_2_∙6H_2_O (> 98%, Sigma-Aldrich), Fe(NO_3_)_3_∙9H_2_O (> 98%, Sigma-Aldrich), and Ni(NO_3_)_2_∙6H_2_O (> 97%, Sigma-Aldrich), were dissolved in deionized water, following the precise stoichiometry required for the intended cation composition. The precursor salts and deionized water were amalgamated at a mass ratio of 1:4. Following this, EDTA (ethylenediaminetetraacetic acid, 99.4 − 100.6%, Sigma-Aldrich) and citric acid (≥ 99.5%, Sigma-Aldrich) were incorporated into the mixture, constituting 60% and 75% of the total molar quantity of the cations, respectively. The mixture was then stirred at 150 rpm, during which NH_4_OH (28 − 30%, Sigma-Aldrich) was gradually introduced until the pH of the solution reached 11, leading to the formation of a dark-colored homogeneous solution.

Gel was obtained from the dark solution by heating and continuous stirring on a hot plate at 200 °C for 5 hr. After removing the magnetic stirring bar, the gel was relocated to a box furnace for an overnight drying process at 300 °C. Subsequently, the dried gel was pulverized and subjected to calcination in a box furnace at 1100 °C for 1 hr (heated up to 800 °C with 10 °C/min and 1100 °C with 5 °C/min), followed by a natural cooling in furnace. The resultant powder of approximately 100 mg was finely ground and placed in a ZrO_2_ crucible. This sample was then re-calcinated at 1100 °C for 1 hr in the box furnace, following the same temperature profile used in the first calcination. The final products, (MgMnCo)_1-x_Fe_x_O_y_ (x = 0.25, 0.35, 0.45) and (MgNiCoFe)O_y_, were designated as MMCF100x and PCO, respectively, where 'x' indicates the Fe content within the 0.25 to 0.45 range. For instance, (MgMnCo)_0.65_Fe_0.35_O_y_ is referred to as MMCF35.

***SEM-EDS***

Surface microstructure and composition of the as-synthesized samples were characterized using field emission scanning electron microscopy (FE-SEM, SU6600, Hitachi) coupled with an energy dispersive X-ray spectroscopy (EDS, Pegasus XM4, EDAX) operating at an accelerated voltage of 10 kV and a beam current of 32 μA. The samples exhibited a homogeneous particles size distribution within 1-2 μm and a uniform elemental distribution (**Figure. S2**). The elemental proportions were in close agreement with the intended compositions (**Table. S1**).

***Redox cycle***

The two-step thermochemical water splitting cycles were conducted using a custom-engineered electrical furnace setup. The setup includes separated two-zone heating units, allowing independent temperature control and rapid temperature ramp through a servo motor-driven zone transition. An R-type thermocouple was positioned directly above the sample to accurately record the *T*_TR_ and *T*_WS_ and to facilitate PID control over the furnace operations. The reactor setup recorded heating and cooling rates over 50 ℃/min (**Figure. S3**).

The reactor setup was integrated into an overall system consisting of gas distribution and analysis units: an electrical switching valve (4 Port Switching Valve, Valco Instruments), a mass flow controller (Digital MFC, MFC Korea), a flow-through humidifier (P-2, Cellkraft AB), an oxygen sensor (ZR5, Zirox), and a gas chromatograph (Agilent 7890B, Agilent) equipped with Hayesep Q and Carboxen1000 columns (**Figure. S3**). In particular, the system delivered ultra-high-purity (UHP) grade argon at a precisely controlled flow rate of 200sccm into two pathways: one for UHP Ar and the other for humidified Ar (*P*(H_2_O) = 0.1 − 0.4 atm) using the humidifier. The switching valve was used to distribute the gases. In the reduction step, UHP Ar was supplied to the reactor maintaining at *P*(O_2_) = 3∙10^-6^−10^-5^ atm while the humidified Ar was vented to the atmosphere. When the water splitting reaction initiated, the switching valve redirected the humidified Ar to the reactor, enabling a step function-like supply of the humidified Ar. All experiments were conducted with a “blank” cycle prior to the reaction without any sample, to eliminate the contributions of any thermolysis and background gases. The blank cycle data were then subtracted from the actual yields (**Figure. S3b**). After the redox cycle was completed, rapid cooling was implemented in an Ar environment to prepare quenched sample for subsequent characterization.

***X-ray diffraction***

The crystal structure of the materials was investigated employing an X-ray diffraction (XRD) system (D/MAX-2500, Rigaku), which is equipped with a copper source. Data acquisition was conducted over a 2θ range from 20˚ to 80˚, employing a step size of 0.02˚, at ambient temperature. The operational parameters for the source were maintained at 40 kV and 200 mA. Phase identification from the XRD patterns utilized the Crystallography Open Database (COD) in conjunction with the JADE software (Materials Data, Inc. (MDI)), leading to the recognition of the rocksalt ($\mathrm{Fm}\bar{3}m$) and spinel ($\mathrm{Fd}\bar{3}m$) phases [10]. Quantitative phase analysis was subsequently conducted through Rietveld refinement, utilizing the Materials Analysis Using Diffraction (MAUD) software [11]. For the refinement results to be considered academically acceptable, both the goodness-of-fit indicator (Sig) and the weighted profile R-factor (R_wp_) must reside within predefined thresholds (Sig < 2 and R_wp_ < 15%). Our fitting results are consistent with these criteria [12].

***X-ray absorption spectroscopy***

Electronic structural analyses were conducted at the 7D and 8C beamlines of the Pohang Light Source (PLS-II, Korea). A silicon (111) double crystal monochromator was employed to monochromatize the incident beam, which was subsequently detuned by 15% to reduce harmonic contamination. Reference materials, including FeO, Fe_2_O_3_, Fe_3_O_4_, CoO, Co_3_O_4_, and NiO powders, were sourced from Sigma-Aldrich. These reference powders, along with the quenched samples, were uniformly distributed and sandwiched between Kapton tapes for the measurements. Beam energy calibration for the sample analyses was facilitated by positioning Fe, Co, Mn, and Ni foils downstream of the samples for Fe K-edge, Co K-edge, Mn K-edge and Ni K-edge measurements, respectively. Data analysis was performed using Athena software, which was employed to normalize the raw data and to determine the edge positions for both the reference materials and the samples using an integration method (**Figure. 5 and Figure. S4**) [13]. All measurements were conducted in transmission mode at room temperature.


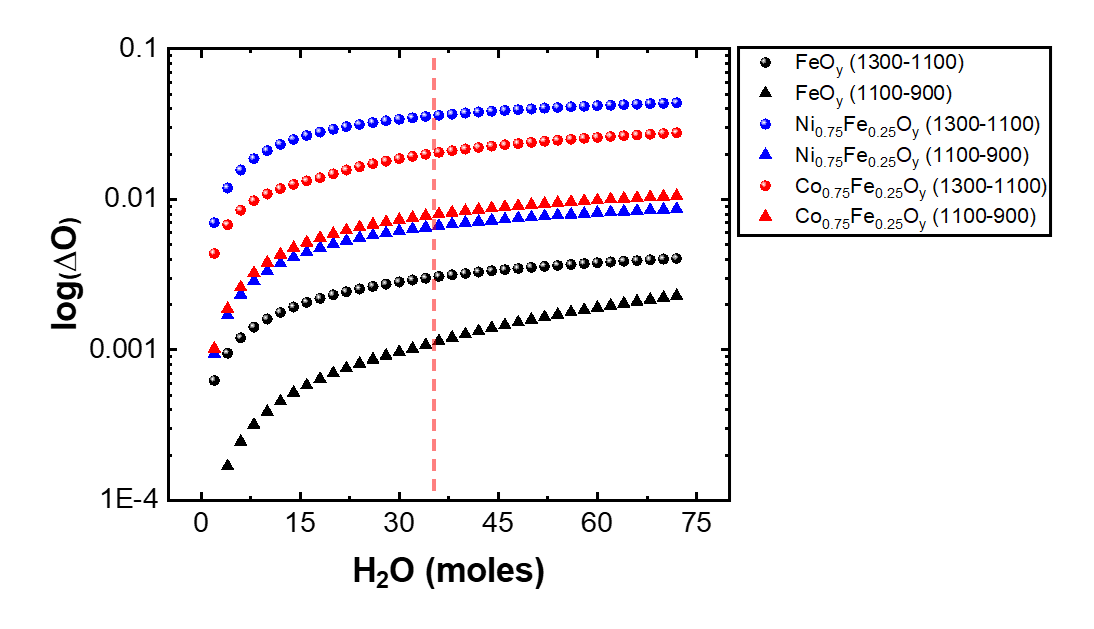


**Figure. S1** Oxygen capacity as a function of moles of H_2_O for representative ferrite systems. The temperature sets in the legend are denoted in the order of *T*_TR_-*T*_WS_ (℃). The red dashed line shows the screening criterion of 35.4 mol-H_2_O.


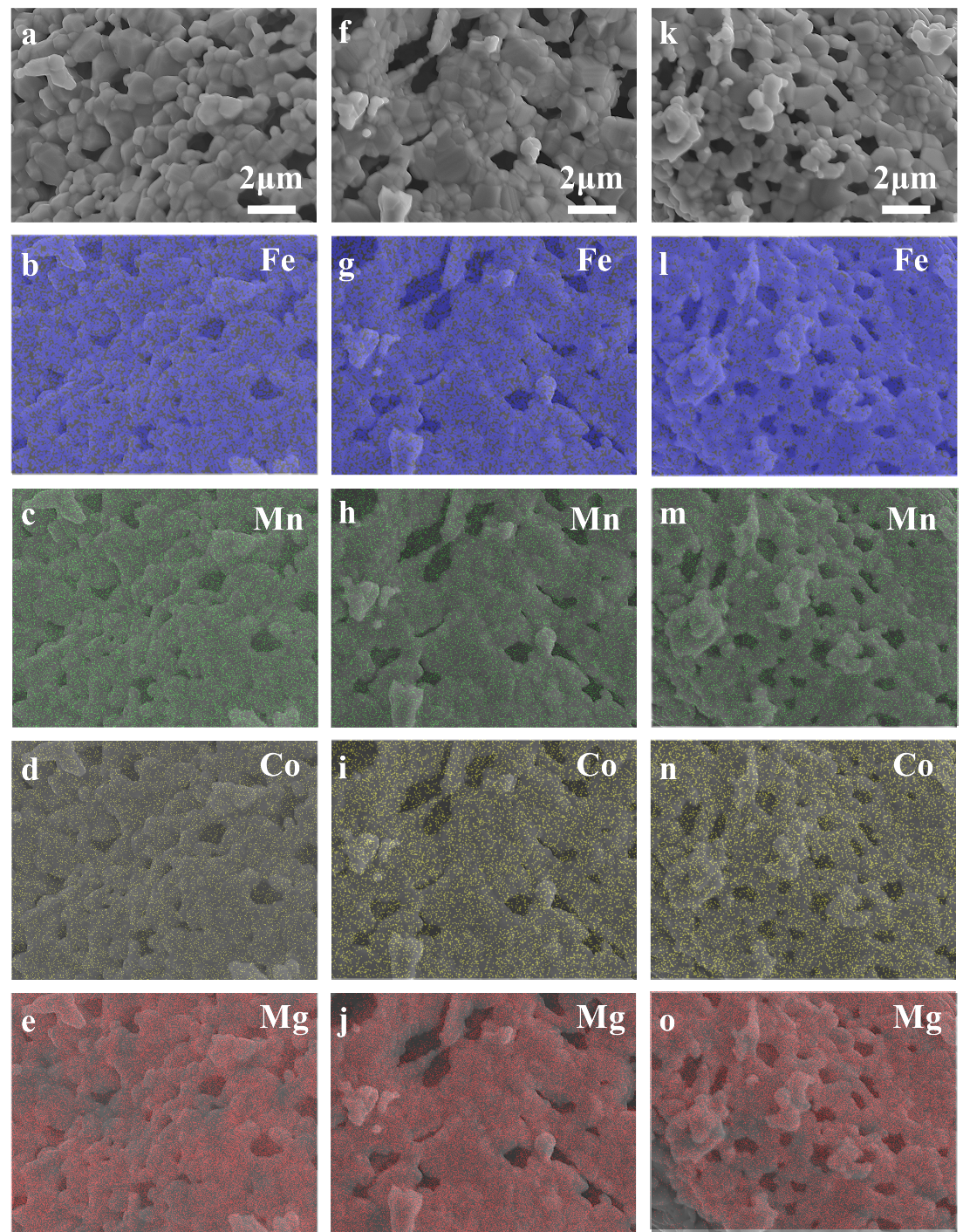


**Figure. S2** SEM images and EDS elemental mapping data of MMCFs. **(a-e)** (MgMnCo)_0.75_Fe_0.25_O_y_ (MMCF25), **(f-j)** (MgMnCo)_0.65_Fe_0.35_O_y_ (MMCF35), **(k-o)** (MgMnCo)_0.55_Fe_0.45_O_y_ (MMCF45)


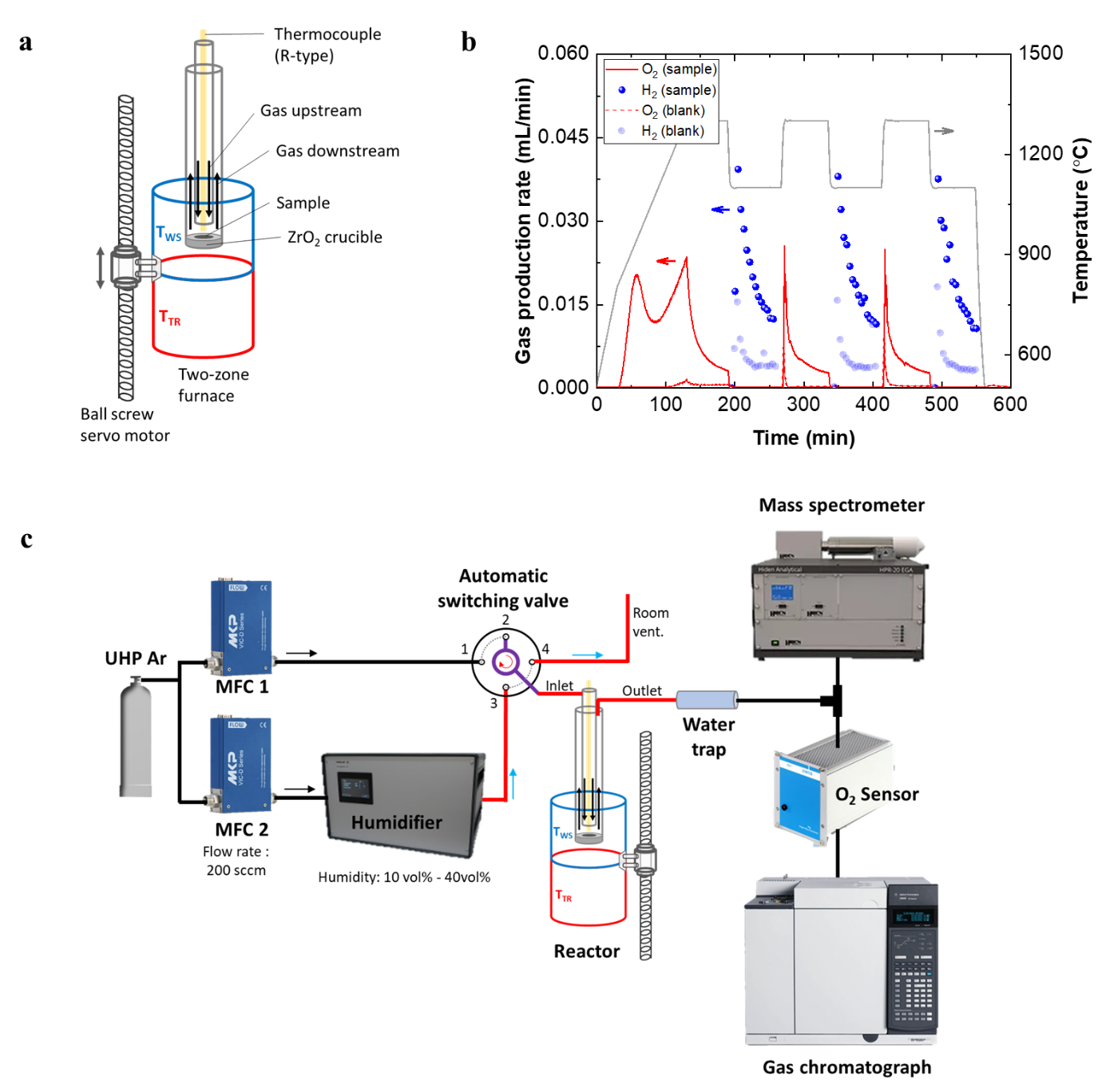


**Figure. S3** **(a)** A custom-engineered reactor setup using two-zone electrical furnace and ball screw-driven servo motor. **(b)** Temperature and gas production profiles; solid lines and data points represent the gas production rate for the sample, while the dashed indicate the blank cycle. **(c)** Schematic of the overall thermochemical reaction system.


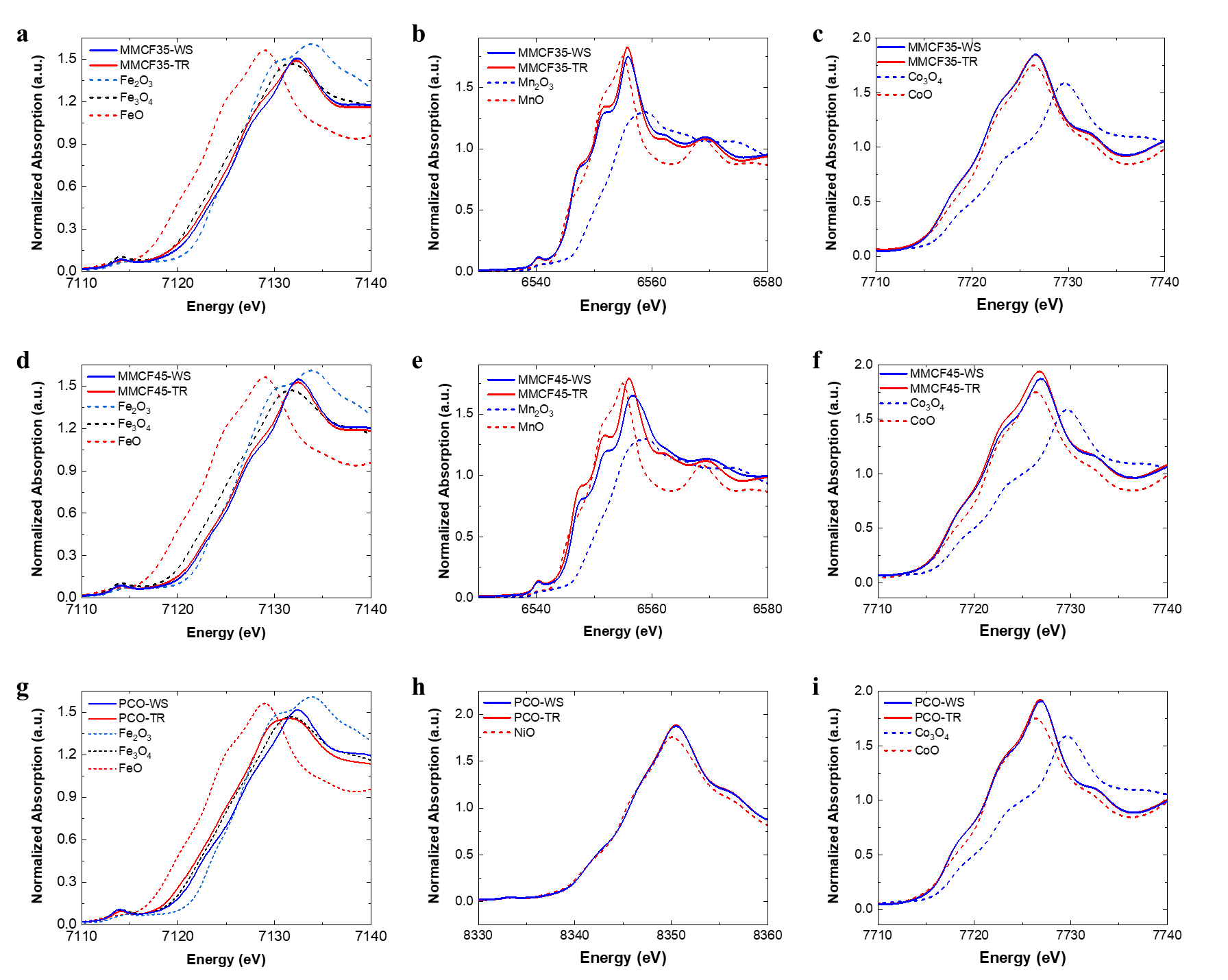


**Figure. S4** X-ray absorption near edge spectroscopy (XANES) results for **(a-c)** MMCF35 at Fe K-edge, Mn K-edge, and Co K-edge, respectively. (*T*_TR_ = 1300 ℃, *T*_WS_ = 1100 ℃, *P*(H_2_O) = 0.1 atm), **(d-f)** MMCF45 at Fe K-edge, Mn K-edge, and Co K-edge, respectively. (*T*_TR_ = 1300 ℃, *T*_WS_ = 1100 ℃, *P*(H_2_O) = 0.3 atm), **(g-i)** PCO at Fe K-edge, Ni K-edge, and Co K-edge, respectively. (*T*_TR_ = 1300 ℃, *T*_WS_ = 1100 ℃, *P*(H_2_O) = 0.3 atm).


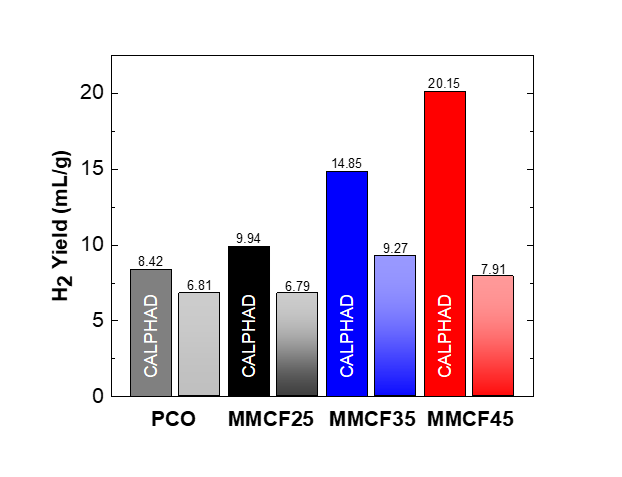


**Figure. S5** Comparison of CALPHAD prediction results and experimental H_2_ yields for PCO and MMCFs at T_TR_ = 1300 ℃, and T_WS_ = 1100 ℃ and P(H_2_O) = 0.3 atm.


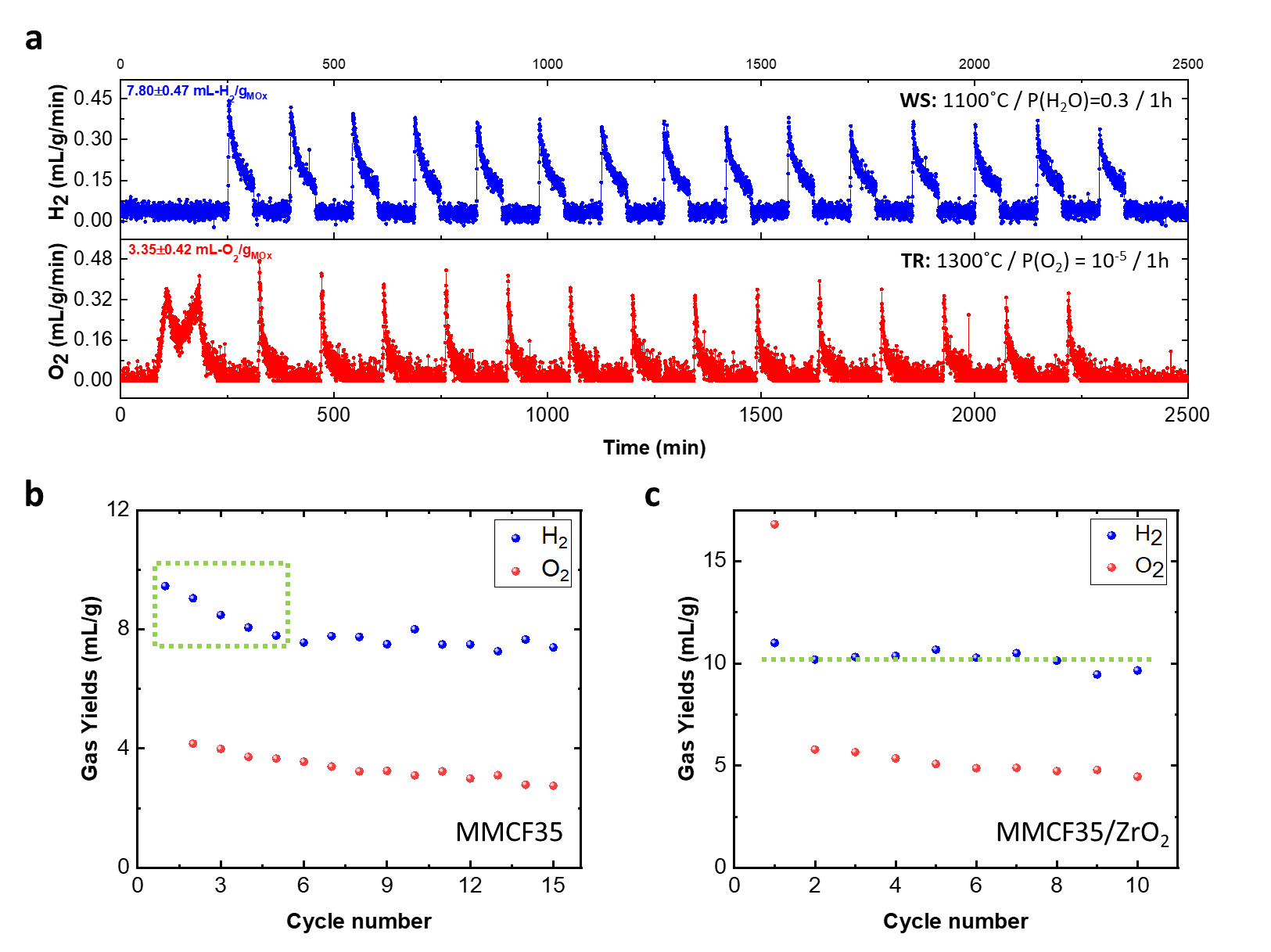


**Figure. S6** (a) Gas evolution rates of MMCF35 over 15 cycles. The evolution rates were quantified by a mass spectrometer (HPR-20, Hiden Analytical). (b) Gas yields per cycle for MMCF35 over 15 cycles. (c) Gas yields for the MMCF35/ZrO2 system over 10 cycles.


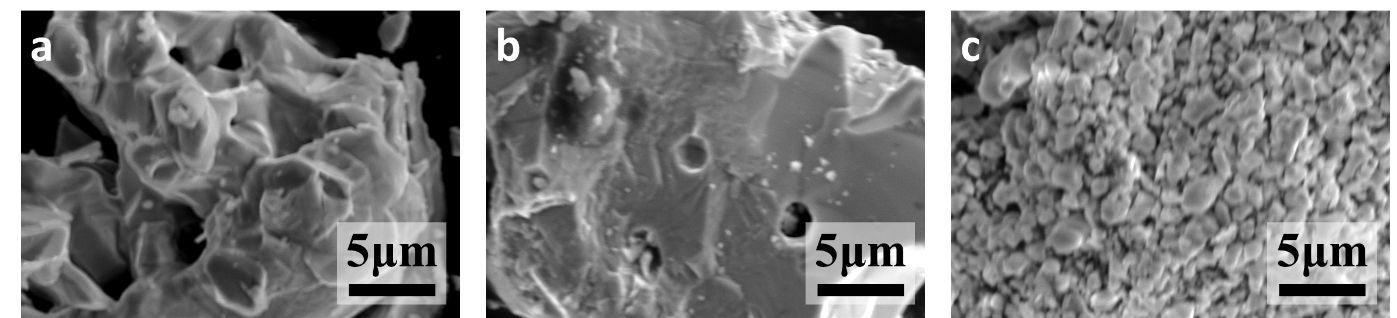


**Figure. S7** (a) SEM images of MMCF35 after 3 cycles, (b) after 15 cycles, and (c) MMCF35/ZrO_2_ composite after 10 cycles.


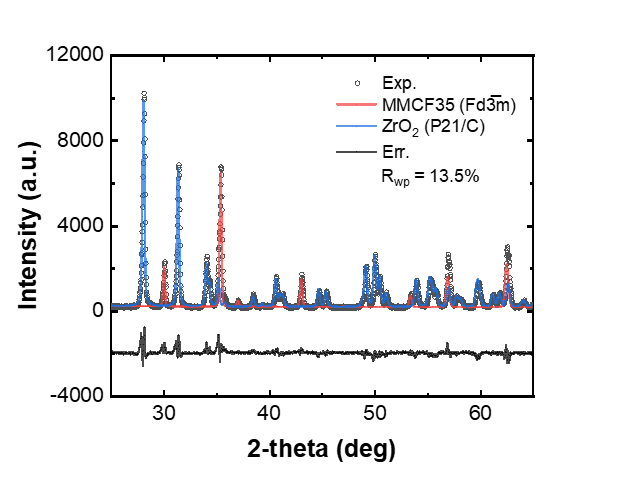


**Figure. S8** XRD pattern of as-synthesized MMCF35/ZrO_2_ composite and corresponding Rietveld refined pattern


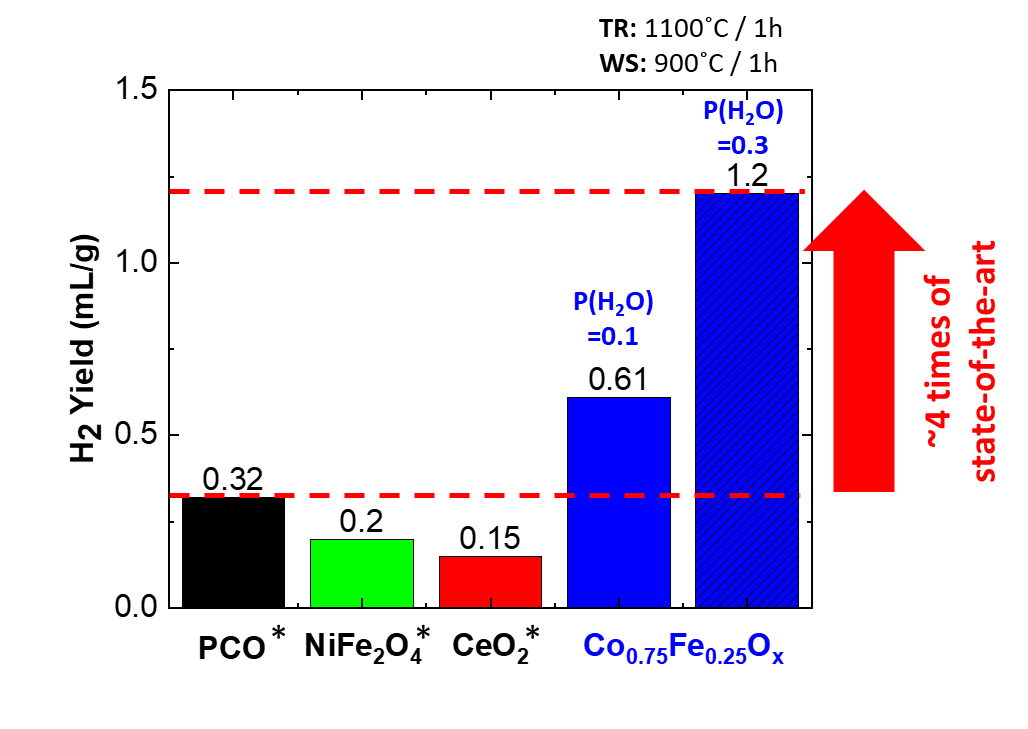


**Figure. S9** Experimental H_2_ yields of Co_0.75_Fe_0.25_O_y_ at T_TR_ = 1100℃ for 1hr and T_WS_ = 900 ℃ for 1hr compared with other benchmark materials. The experimental conditions for the benchmark materials (*) are T_TR_ = 1100℃ and P(O_2_) = 10^-5^ atm for 1hr, and T_WS_ = 800 ℃, H_2_:H_2_O = 1:2.1∙10^4^ for 1hr, and data were sourced from the literature.[4]

**
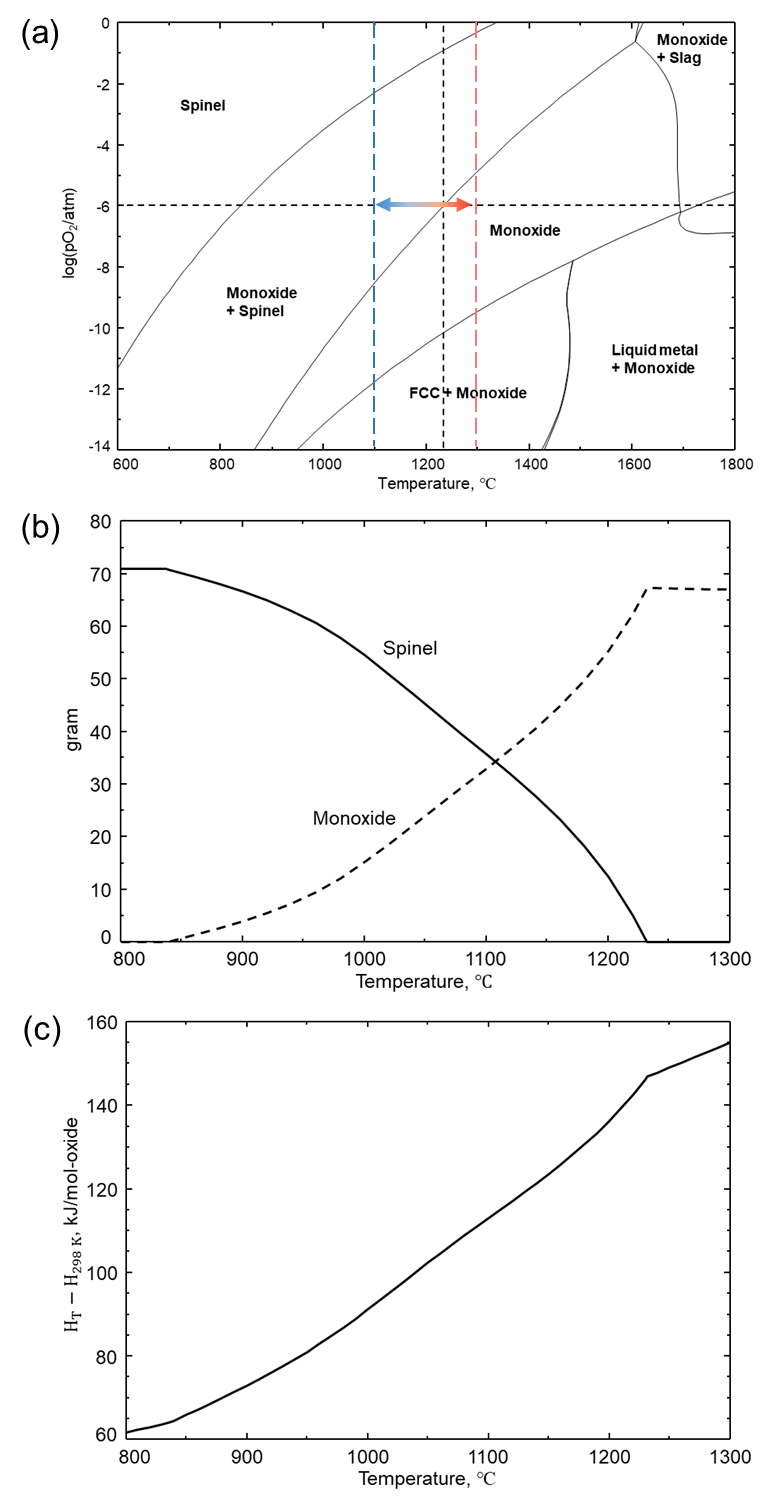
**

**Figure. S10** Calculated (a) phase diagram depending on temperature and oxygen partial pressure, (b) equilibrium phase amount and (c) enthalpy change of (MgMnCo)_0.65_Fe_0.35_O_y_ at 1231 ℃. The spinel-to-monoxide phase transition completes at 1231 ℃, which corresponds to the change in the slope of the enthalpy curve. This change in slope causes an abrupt drop in the calculated *C*_P_ of (MgMnCo)_0.65_Fe_0.35_O_y_


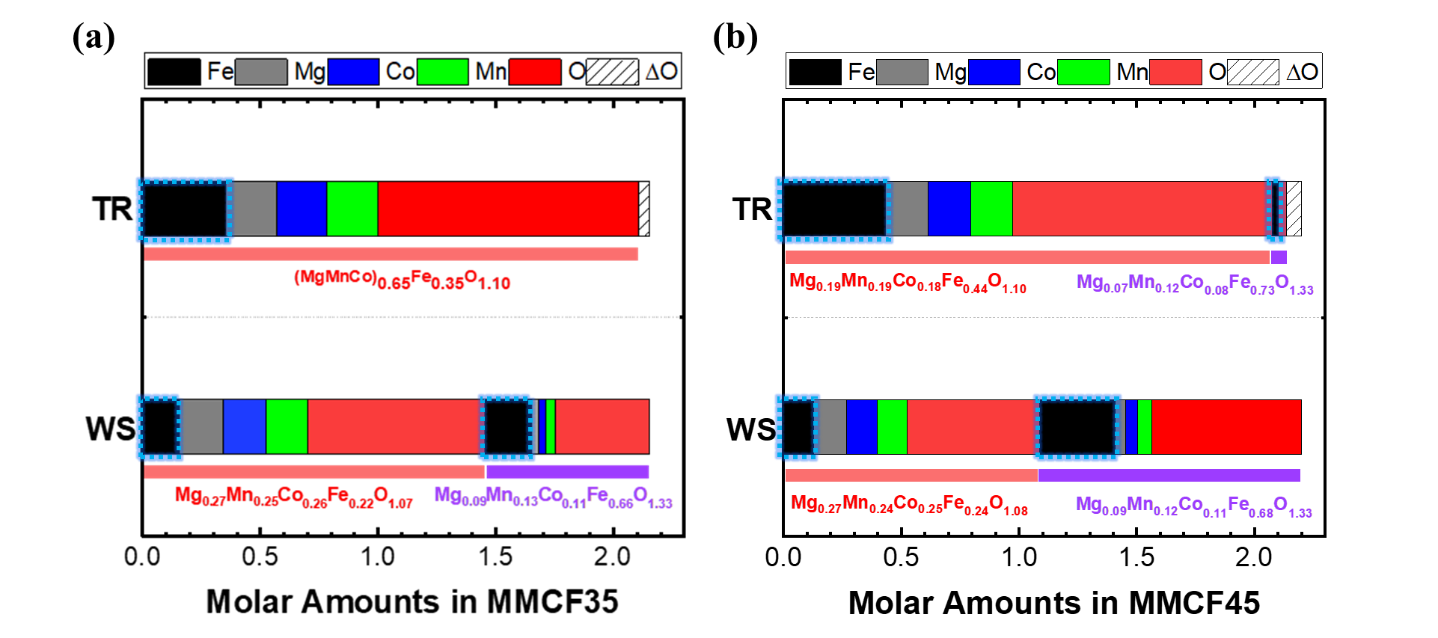


**Figure. S11** Calculated cation distributions for MMCFs: (a) for MMCF35 and (b) for MMCF45, obtained using the CALPHAD method. Red and violet bars represent the rocksalt and spinel phases, respectively.


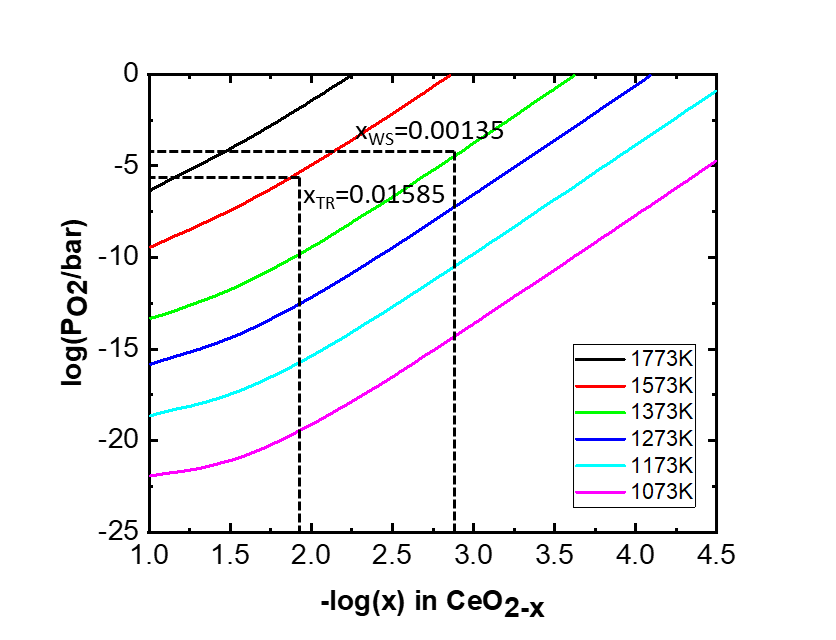


**Figure. S12** Theoretical oxygen non-stoichiometry of CeO_2_.[14]


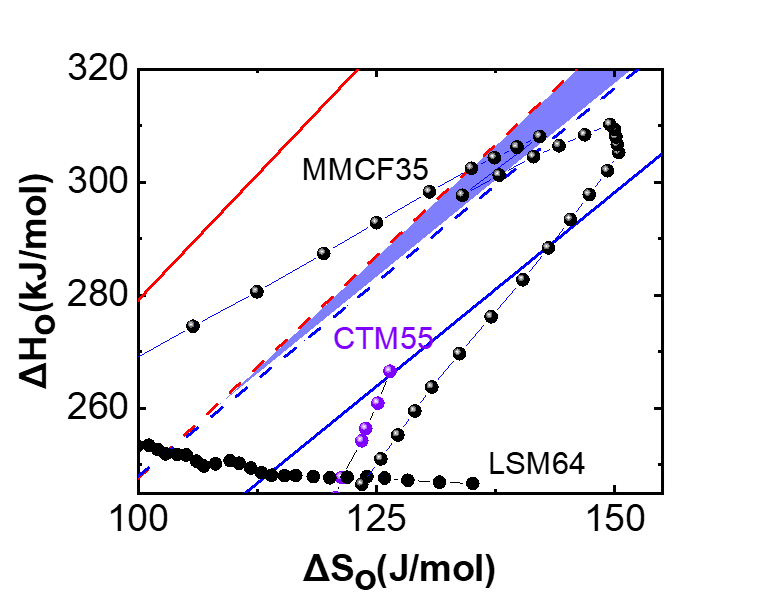


**Figure. S13** Thermodynamic characteristics of (MgMnCo)_0.65_Fe_0.35_O_y_ compared to other benchmarked materials in high background H_2_ region. Red solid line and dashed line indicate the region where ΔG is zero for the thermal reduction at T_TR_ = 1500 ℃ and T_TR_ = 1300 ℃ at P(O_2_) = 10^-6^ atm, respectively. Blue solid and dashed line indicate the region where the ΔG is zero for the water splitting at H_2_O:H_2_=1000:1 and 200:1 at T_WS_ = 1100 ℃, respectively.

| Materials | Element | Wt% | At% |
| --- | --- | --- | --- |
| MMCF25 | Mg | 11.32 | 22.9 |
|  | Mn | 27.57 | 24.69 |
|  | Fe | 30.08 | 26.5 |
|  | Co | 31.03 | 25.91 |
| MMCF35 | Mg | 11.83 | 23.77 |
|  | Mn | 22.25 | 19.77 |
|  | Fe | 40.02 | 35 |
|  | Co | 25.9 | 21.46 |
| MMCF45 | Mg | 9.56 | 19.69 |
|  | Mn | 18.76 | 17.1 |
|  | Fe | 49.64 | 44.5 |
|  | Co | 22.04 | 18.72 |

**Table. S1** EDS results for as-synthesized MMCFs.

| Rxn. | Site | Fe^2+^ | Fe^3+^ | Mg^2+^ | Co^2+^ | Co^3+^ | Mn^2+^ | Mn^3+^ | Mn^4+^ |
| --- | --- | --- | --- | --- | --- | --- | --- | --- | --- |
| TR | Spinel (oct) | 0.051 | 0.434 | 0.058 | 0.036 | 0.001 | 0.057 | 0.024 | 0.006 |
|  | Spinel (tet) | 0.033 | 0.197 | 0.014 | 0.040 | - | 0.049 | - | - |
|  | Rocksalt | 0.196 | 0.095 | 0.239 | 0.239 | - | 0.224 | 0.008 | - |
| WS | Spinel (oct) | 0.036 | 0.617 | 0.123 | 0.071 | 0.001 | 0.090 | 0.053 | 0.010 |
|  | Spinel (tet) | 0.051 | 0.620 | 0.038 | 0.192 | - | 0.100 | - | - |
|  | Rocksalt | 0.092 | 0.144 | 0.290 | 0.281 | - | 0.257 | 0.014 | - |

**Table. S2** Cation distribution of MMCF35 by CALPHAD simulation.

| Rxn. | Site | Fe^2+^ | Fe^3+^ | Mg^2+^ | Co^2+^ | Co^3+^ | Mn^2+^ | Mn^3+^ | Mn^4+^ |
| --- | --- | --- | --- | --- | --- | --- | --- | --- | --- |
| TR | Spinel (oct) | 0.064 | 0.442 | 0.053 | 0.035 | 0.000 | 0.051 | 0.017 | 0.004 |
|  | Spinel (tet) | 0.041 | 0.200 | 0.012 | 0.033 | - | 0.047 | - | - |
|  | Rocksalt | 0.254 | 0.128 | 0.208 | 0.208 | - | 0.194 | 0.007 | - |
| WS | Spinel (oct) | 0.044 | 0.628 | 0.120 | 0.072 | 0.001 | 0.085 | 0.043 | 0.007 |
|  | Spinel (tet) | 0.059 | 0.627 | 0.037 | 0.178 | - | 0.100 | - | - |
|  | Rocksalt | 0.111 | 0.151 | 0.288 | 0.273 | - | 0.246 | 0.012 | - |

**Table. S3** Cation distribution of MMCF45 by CALPHAD simulation.

**Table. S4** CALPHAD simulation results for M_1-x_Fe_x_O_y_ system which contains Mn. P(O_2_) and input H_2_O were set at 3∙10^-6^ atm and 35.4 mol, respectively.

| Composition M(1-x)Fe(x)O(y) | | | | | | | TR | | WS | |
| --- | --- | --- | --- | --- | --- | --- | --- | --- | --- | --- |
| x | Fe | Al | Mg | Co | Ni | Mn | T(TR) (℃) | O stoich. | T(WS) (℃) | O stoich. |
| 0.050 | 0.050 | 0.000 | 0.000 | 0.000 | 0.000 | 0.950 | 1300 | 1.02093 | 1300 | 1.04480 |
| 0.150 | 0.150 | 0.000 | 0.000 | 0.000 | 0.000 | 0.850 | 1300 | 1.05577 | 1300 | 1.09080 |
| 0.250 | 0.250 | 0.000 | 0.000 | 0.000 | 0.000 | 0.750 | 1300 | 1.09949 | 1300 | 1.14407 |
| 0.350 | 0.350 | 0.000 | 0.000 | 0.000 | 0.000 | 0.650 | 1300 | 1.15168 | 1300 | 1.20667 |
| 0.450 | 0.450 | 0.000 | 0.000 | 0.000 | 0.000 | 0.550 | 1300 | 1.22628 | 1300 | 1.28355 |
| 0.550 | 0.550 | 0.000 | 0.000 | 0.000 | 0.000 | 0.450 | 1300 | 1.30088 | 1300 | 1.33304 |
| 0.650 | 0.650 | 0.000 | 0.000 | 0.000 | 0.000 | 0.350 | 1300 | 1.33328 | 1300 | 1.33308 |
| 0.750 | 0.750 | 0.000 | 0.000 | 0.000 | 0.000 | 0.250 | 1300 | 1.33329 | 1300 | 1.33305 |
| 0.850 | 0.850 | 0.000 | 0.000 | 0.000 | 0.000 | 0.150 | 1300 | 1.33330 | 1300 | 1.33314 |
| 0.950 | 0.950 | 0.000 | 0.000 | 0.000 | 0.000 | 0.050 | 1300 | 1.33335 | 1300 | 1.33387 |
| 0.050 | 0.050 | 0.475 | 0.000 | 0.000 | 0.000 | 0.475 | 1300 | 1.33328 | 1300 | 1.33325 |
| 0.150 | 0.150 | 0.425 | 0.000 | 0.000 | 0.000 | 0.425 | 1300 | 1.33328 | 1300 | 1.33319 |
| 0.250 | 0.250 | 0.375 | 0.000 | 0.000 | 0.000 | 0.375 | 1300 | 1.33328 | 1300 | 1.33314 |
| 0.350 | 0.350 | 0.325 | 0.000 | 0.000 | 0.000 | 0.325 | 1300 | 1.33328 | 1300 | 1.33311 |
| 0.450 | 0.450 | 0.275 | 0.000 | 0.000 | 0.000 | 0.275 | 1300 | 1.33329 | 1300 | 1.33310 |
| 0.550 | 0.550 | 0.225 | 0.000 | 0.000 | 0.000 | 0.225 | 1300 | 1.33329 | 1300 | 1.33311 |
| 0.650 | 0.650 | 0.175 | 0.000 | 0.000 | 0.000 | 0.175 | 1300 | 1.33330 | 1300 | 1.33318 |
| 0.750 | 0.750 | 0.125 | 0.000 | 0.000 | 0.000 | 0.125 | 1300 | 1.33331 | 1300 | 1.33335 |
| 0.850 | 0.850 | 0.075 | 0.000 | 0.000 | 0.000 | 0.075 | 1300 | 1.33334 | 1300 | 1.33372 |
| 0.950 | 0.950 | 0.025 | 0.000 | 0.000 | 0.000 | 0.025 | 1300 | 1.33340 | 1300 | 1.33441 |
| 0.050 | 0.050 | 0.000 | 0.475 | 0.000 | 0.000 | 0.475 | 1300 | 1.01425 | 1300 | 1.02953 |
| 0.150 | 0.150 | 0.000 | 0.425 | 0.000 | 0.000 | 0.425 | 1300 | 1.04119 | 1300 | 1.06668 |
| 0.250 | 0.250 | 0.000 | 0.375 | 0.000 | 0.000 | 0.375 | 1300 | 1.07727 | 1300 | 1.10692 |
| 0.350 | 0.350 | 0.000 | 0.325 | 0.000 | 0.000 | 0.325 | 1300 | 1.11350 | 1300 | 1.14926 |
| 0.450 | 0.450 | 0.000 | 0.275 | 0.000 | 0.000 | 0.275 | 1300 | 1.15588 | 1300 | 1.19988 |
| 0.550 | 0.550 | 0.000 | 0.225 | 0.000 | 0.000 | 0.225 | 1300 | 1.21955 | 1300 | 1.26064 |
| 0.650 | 0.650 | 0.000 | 0.175 | 0.000 | 0.000 | 0.175 | 1300 | 1.28118 | 1300 | 1.31846 |
| 0.750 | 0.750 | 0.000 | 0.125 | 0.000 | 0.000 | 0.125 | 1300 | 1.33329 | 1300 | 1.33306 |
| 0.850 | 0.850 | 0.000 | 0.075 | 0.000 | 0.000 | 0.075 | 1300 | 1.33330 | 1300 | 1.33323 |
| 0.950 | 0.950 | 0.000 | 0.025 | 0.000 | 0.000 | 0.025 | 1300 | 1.33336 | 1300 | 1.33401 |
| 0.050 | 0.050 | 0.000 | 0.000 | 0.475 | 0.000 | 0.475 | 1300 | 1.02346 | 1300 | 1.04282 |
| 0.150 | 0.150 | 0.000 | 0.000 | 0.425 | 0.000 | 0.425 | 1300 | 1.05059 | 1300 | 1.07488 |
| 0.250 | 0.250 | 0.000 | 0.000 | 0.375 | 0.000 | 0.375 | 1300 | 1.07869 | 1300 | 1.10497 |
| 0.350 | 0.350 | 0.000 | 0.000 | 0.325 | 0.000 | 0.325 | 1300 | 1.10564 | 1300 | 1.14196 |
| 0.450 | 0.450 | 0.000 | 0.000 | 0.275 | 0.000 | 0.275 | 1300 | 1.14376 | 1300 | 1.19105 |
| 0.550 | 0.550 | 0.000 | 0.000 | 0.225 | 0.000 | 0.225 | 1300 | 1.21211 | 1300 | 1.25666 |
| 0.650 | 0.650 | 0.000 | 0.000 | 0.175 | 0.000 | 0.175 | 1300 | 1.27998 | 1300 | 1.32096 |
| 0.750 | 0.750 | 0.000 | 0.000 | 0.125 | 0.000 | 0.125 | 1300 | 1.33329 | 1300 | 1.33307 |
| 0.850 | 0.850 | 0.000 | 0.000 | 0.075 | 0.000 | 0.075 | 1300 | 1.33330 | 1300 | 1.33323 |
| 0.950 | 0.950 | 0.000 | 0.000 | 0.025 | 0.000 | 0.025 | 1300 | 1.33336 | 1300 | 1.33400 |
| 0.050 | 0.050 | 0.000 | 0.000 | 0.000 | 0.475 | 0.475 | 1300 | 1.01439 | 1300 | 1.02945 |
| 0.150 | 0.150 | 0.000 | 0.000 | 0.000 | 0.425 | 0.425 | 1300 | 1.03827 | 1300 | 1.06158 |
| 0.250 | 0.250 | 0.000 | 0.000 | 0.000 | 0.375 | 0.375 | 1300 | 1.06974 | 1300 | 1.09865 |
| 0.350 | 0.350 | 0.000 | 0.000 | 0.000 | 0.325 | 0.325 | 1300 | 1.10257 | 1300 | 1.14473 |
| 0.450 | 0.450 | 0.000 | 0.000 | 0.000 | 0.275 | 0.275 | 1300 | 1.16466 | 1300 | 1.20560 |
| 0.550 | 0.550 | 0.000 | 0.000 | 0.000 | 0.225 | 0.225 | 1300 | 1.22789 | 1300 | 1.26522 |
| 0.650 | 0.650 | 0.000 | 0.000 | 0.000 | 0.175 | 0.175 | 1300 | 1.28882 | 1300 | 1.32105 |
| 0.750 | 0.750 | 0.000 | 0.000 | 0.000 | 0.125 | 0.125 | 1300 | 1.33329 | 1300 | 1.33287 |
| 0.850 | 0.850 | 0.000 | 0.000 | 0.000 | 0.075 | 0.075 | 1300 | 1.33330 | 1300 | 1.33314 |
| 0.950 | 0.950 | 0.000 | 0.000 | 0.000 | 0.025 | 0.025 | 1300 | 1.33336 | 1300 | 1.33398 |
| 0.050 | 0.050 | 0.317 | 0.317 | 0.000 | 0.000 | 0.317 | 1300 | 1.19769 | 1300 | 1.22892 |
| 0.150 | 0.150 | 0.283 | 0.283 | 0.000 | 0.000 | 0.283 | 1300 | 1.22254 | 1300 | 1.25877 |
| 0.250 | 0.250 | 0.250 | 0.250 | 0.000 | 0.000 | 0.250 | 1300 | 1.25487 | 1300 | 1.29199 |
| 0.350 | 0.350 | 0.217 | 0.217 | 0.000 | 0.000 | 0.217 | 1300 | 1.28817 | 1300 | 1.32377 |
| 0.450 | 0.450 | 0.183 | 0.183 | 0.000 | 0.000 | 0.183 | 1300 | 1.31946 | 1300 | 1.33307 |
| 0.550 | 0.550 | 0.150 | 0.150 | 0.000 | 0.000 | 0.150 | 1300 | 1.33329 | 1300 | 1.33309 |
| 0.650 | 0.650 | 0.117 | 0.117 | 0.000 | 0.000 | 0.117 | 1300 | 1.33329 | 1300 | 1.33314 |
| 0.750 | 0.750 | 0.083 | 0.083 | 0.000 | 0.000 | 0.083 | 1300 | 1.33331 | 1300 | 1.33329 |
| 0.850 | 0.850 | 0.050 | 0.050 | 0.000 | 0.000 | 0.050 | 1300 | 1.33333 | 1300 | 1.33363 |
| 0.950 | 0.950 | 0.017 | 0.017 | 0.000 | 0.000 | 0.017 | 1300 | 1.33339 | 1300 | 1.33433 |
| 0.050 | 0.050 | 0.317 | 0.000 | 0.317 | 0.000 | 0.317 | 1300 | 1.19797 | 1300 | 1.22764 |
| 0.150 | 0.150 | 0.283 | 0.000 | 0.283 | 0.000 | 0.283 | 1300 | 1.21958 | 1300 | 1.25295 |
| 0.250 | 0.250 | 0.250 | 0.000 | 0.250 | 0.000 | 0.250 | 1300 | 1.24656 | 1300 | 1.28186 |
| 0.350 | 0.350 | 0.217 | 0.000 | 0.217 | 0.000 | 0.217 | 1300 | 1.27791 | 1300 | 1.31310 |
| 0.450 | 0.450 | 0.183 | 0.000 | 0.183 | 0.000 | 0.183 | 1300 | 1.31069 | 1300 | 1.33307 |
| 0.550 | 0.550 | 0.150 | 0.000 | 0.150 | 0.000 | 0.150 | 1300 | 1.33328 | 1300 | 1.33310 |
| 0.650 | 0.650 | 0.117 | 0.000 | 0.117 | 0.000 | 0.117 | 1300 | 1.33329 | 1300 | 1.33312 |
| 0.750 | 0.750 | 0.083 | 0.000 | 0.083 | 0.000 | 0.083 | 1300 | 1.33330 | 1300 | 1.33326 |
| 0.850 | 0.850 | 0.050 | 0.000 | 0.050 | 0.000 | 0.050 | 1300 | 1.33333 | 1300 | 1.33360 |
| 0.950 | 0.950 | 0.017 | 0.000 | 0.017 | 0.000 | 0.017 | 1300 | 1.33339 | 1300 | 1.33431 |
| 0.050 | 0.050 | 0.317 | 0.000 | 0.000 | 0.317 | 0.317 | 1300 | 1.18564 | 1300 | 1.20883 |
| 0.150 | 0.150 | 0.283 | 0.000 | 0.000 | 0.283 | 0.283 | 1300 | 1.20654 | 1300 | 1.23344 |
| 0.250 | 0.250 | 0.250 | 0.000 | 0.000 | 0.250 | 0.250 | 1300 | 1.23585 | 1300 | 1.26364 |
| 0.350 | 0.350 | 0.217 | 0.000 | 0.000 | 0.217 | 0.217 | 1300 | 1.26856 | 1300 | 1.29592 |
| 0.450 | 0.450 | 0.183 | 0.000 | 0.000 | 0.183 | 0.183 | 1300 | 1.30179 | 1300 | 1.32812 |
| 0.550 | 0.550 | 0.150 | 0.000 | 0.000 | 0.150 | 0.150 | 1300 | 1.33329 | 1300 | 1.33285 |
| 0.650 | 0.650 | 0.117 | 0.000 | 0.000 | 0.117 | 0.117 | 1300 | 1.33329 | 1300 | 1.33300 |
| 0.750 | 0.750 | 0.083 | 0.000 | 0.000 | 0.083 | 0.083 | 1300 | 1.33330 | 1300 | 1.33320 |
| 0.850 | 0.850 | 0.050 | 0.000 | 0.000 | 0.050 | 0.050 | 1300 | 1.33333 | 1300 | 1.33357 |
| 0.950 | 0.950 | 0.017 | 0.000 | 0.000 | 0.017 | 0.017 | 1300 | 1.33339 | 1300 | 1.33431 |
| 0.050 | 0.050 | 0.000 | 0.317 | 0.317 | 0.000 | 0.317 | 1300 | 1.01670 | 1300 | 1.03094 |
| 0.150 | 0.150 | 0.000 | 0.283 | 0.283 | 0.000 | 0.283 | 1300 | 1.04435 | 1300 | 1.06622 |
| 0.250 | 0.250 | 0.000 | 0.250 | 0.250 | 0.000 | 0.250 | 1300 | 1.07574 | 1300 | 1.10099 |
| 0.350 | 0.350 | 0.000 | 0.217 | 0.217 | 0.000 | 0.217 | 1300 | 1.10602 | 1300 | 1.13739 |
| 0.450 | 0.450 | 0.000 | 0.183 | 0.183 | 0.000 | 0.183 | 1300 | 1.13627 | 1300 | 1.18233 |
| 0.550 | 0.550 | 0.000 | 0.150 | 0.150 | 0.000 | 0.150 | 1300 | 1.20462 | 1300 | 1.24750 |
| 0.650 | 0.650 | 0.000 | 0.117 | 0.117 | 0.000 | 0.117 | 1300 | 1.27226 | 1300 | 1.31101 |
| 0.750 | 0.750 | 0.000 | 0.083 | 0.083 | 0.000 | 0.083 | 1300 | 1.33329 | 1300 | 1.33306 |
| 0.850 | 0.850 | 0.000 | 0.050 | 0.050 | 0.000 | 0.050 | 1300 | 1.33330 | 1300 | 1.33324 |
| 0.950 | 0.950 | 0.000 | 0.017 | 0.017 | 0.000 | 0.017 | 1300 | 1.33337 | 1300 | 1.33404 |
| 0.050 | 0.050 | 0.000 | 0.317 | 0.000 | 0.317 | 0.317 | 1300 | 1.01219 | 1300 | 1.02436 |
| 0.150 | 0.150 | 0.000 | 0.283 | 0.000 | 0.283 | 0.283 | 1300 | 1.03796 | 1300 | 1.05971 |
| 0.250 | 0.250 | 0.000 | 0.250 | 0.000 | 0.250 | 0.250 | 1300 | 1.07260 | 1300 | 1.09840 |
| 0.350 | 0.350 | 0.000 | 0.217 | 0.000 | 0.217 | 0.217 | 1300 | 1.10724 | 1300 | 1.14109 |
| 0.450 | 0.450 | 0.000 | 0.183 | 0.000 | 0.183 | 0.183 | 1300 | 1.15216 | 1300 | 1.19244 |
| 0.550 | 0.550 | 0.000 | 0.150 | 0.000 | 0.150 | 0.150 | 1300 | 1.21391 | 1300 | 1.25088 |
| 0.650 | 0.650 | 0.000 | 0.117 | 0.000 | 0.117 | 0.117 | 1300 | 1.27378 | 1300 | 1.30681 |
| 0.750 | 0.750 | 0.000 | 0.083 | 0.000 | 0.083 | 0.083 | 1300 | 1.33112 | 1300 | 1.33294 |
| 0.850 | 0.850 | 0.000 | 0.050 | 0.000 | 0.050 | 0.050 | 1300 | 1.33330 | 1300 | 1.33320 |
| 0.950 | 0.950 | 0.000 | 0.017 | 0.000 | 0.017 | 0.017 | 1300 | 1.33337 | 1300 | 1.33403 |
| 0.050 | 0.050 | 0.000 | 0.000 | 0.317 | 0.317 | 0.317 | 1300 | 1.01671 | 1300 | 1.03098 |
| 0.150 | 0.150 | 0.000 | 0.000 | 0.283 | 0.283 | 0.283 | 1300 | 1.04203 | 1300 | 1.06249 |
| 0.250 | 0.250 | 0.000 | 0.000 | 0.250 | 0.250 | 0.250 | 1300 | 1.07083 | 1300 | 1.09410 |
| 0.350 | 0.350 | 0.000 | 0.000 | 0.217 | 0.217 | 0.217 | 1300 | 1.09947 | 1300 | 1.13772 |
| 0.450 | 0.450 | 0.000 | 0.000 | 0.183 | 0.183 | 0.183 | 1300 | 1.15065 | 1300 | 1.19471 |
| 0.550 | 0.550 | 0.000 | 0.000 | 0.150 | 0.150 | 0.150 | 1300 | 1.21920 | 1300 | 1.26028 |
| 0.650 | 0.650 | 0.000 | 0.000 | 0.117 | 0.117 | 0.117 | 1300 | 1.28729 | 1300 | 1.32449 |
| 0.750 | 0.750 | 0.000 | 0.000 | 0.083 | 0.083 | 0.083 | 1300 | 1.33329 | 1300 | 1.33296 |
| 0.850 | 0.850 | 0.000 | 0.000 | 0.050 | 0.050 | 0.050 | 1300 | 1.33330 | 1300 | 1.33319 |
| 0.950 | 0.950 | 0.000 | 0.000 | 0.017 | 0.017 | 0.017 | 1300 | 1.33337 | 1300 | 1.33402 |
| 0.050 | 0.050 | 0.238 | 0.238 | 0.238 | 0.000 | 0.238 | 1300 | 1.14670 | 1300 | 1.16922 |
| 0.150 | 0.150 | 0.213 | 0.213 | 0.213 | 0.000 | 0.213 | 1300 | 1.16929 | 1300 | 1.19818 |
| 0.250 | 0.250 | 0.188 | 0.188 | 0.188 | 0.000 | 0.188 | 1300 | 1.19942 | 1300 | 1.23244 |
| 0.350 | 0.350 | 0.163 | 0.163 | 0.163 | 0.000 | 0.163 | 1300 | 1.23686 | 1300 | 1.27023 |
| 0.450 | 0.450 | 0.138 | 0.138 | 0.138 | 0.000 | 0.138 | 1300 | 1.27525 | 1300 | 1.30782 |
| 0.550 | 0.550 | 0.113 | 0.113 | 0.113 | 0.000 | 0.113 | 1300 | 1.31388 | 1300 | 1.33305 |
| 0.650 | 0.650 | 0.088 | 0.088 | 0.088 | 0.000 | 0.088 | 1300 | 1.33329 | 1300 | 1.33309 |
| 0.750 | 0.750 | 0.063 | 0.063 | 0.063 | 0.000 | 0.063 | 1300 | 1.33330 | 1300 | 1.33319 |
| 0.850 | 0.850 | 0.038 | 0.038 | 0.038 | 0.000 | 0.038 | 1300 | 1.33332 | 1300 | 1.33352 |
| 0.950 | 0.950 | 0.013 | 0.013 | 0.013 | 0.000 | 0.013 | 1300 | 1.33339 | 1300 | 1.33427 |
| 0.050 | 0.050 | 0.238 | 0.238 | 0.000 | 0.238 | 0.238 | 1300 | 1.14148 | 1300 | 1.16101 |
| 0.150 | 0.150 | 0.213 | 0.213 | 0.000 | 0.213 | 0.213 | 1300 | 1.16499 | 1300 | 1.19159 |
| 0.250 | 0.250 | 0.188 | 0.188 | 0.000 | 0.188 | 0.188 | 1300 | 1.19895 | 1300 | 1.22808 |
| 0.350 | 0.350 | 0.163 | 0.163 | 0.000 | 0.163 | 0.163 | 1300 | 1.23625 | 1300 | 1.26516 |
| 0.450 | 0.450 | 0.138 | 0.138 | 0.000 | 0.138 | 0.138 | 1300 | 1.27281 | 1300 | 1.30091 |
| 0.550 | 0.550 | 0.113 | 0.113 | 0.000 | 0.113 | 0.113 | 1300 | 1.30864 | 1300 | 1.33276 |
| 0.650 | 0.650 | 0.088 | 0.088 | 0.000 | 0.088 | 0.088 | 1300 | 1.33329 | 1300 | 1.33299 |
| 0.750 | 0.750 | 0.063 | 0.063 | 0.000 | 0.063 | 0.063 | 1300 | 1.33330 | 1300 | 1.33316 |
| 0.850 | 0.850 | 0.038 | 0.038 | 0.000 | 0.038 | 0.038 | 1300 | 1.33333 | 1300 | 1.33351 |
| 0.950 | 0.950 | 0.013 | 0.013 | 0.000 | 0.013 | 0.013 | 1300 | 1.33339 | 1300 | 1.33427 |
| 0.050 | 0.050 | 0.238 | 0.000 | 0.238 | 0.238 | 0.238 | 1300 | 1.14317 | 1300 | 1.16327 |
| 0.150 | 0.150 | 0.213 | 0.000 | 0.213 | 0.213 | 0.213 | 1300 | 1.16721 | 1300 | 1.19433 |
| 0.250 | 0.250 | 0.188 | 0.000 | 0.188 | 0.188 | 0.188 | 1300 | 1.20012 | 1300 | 1.23109 |
| 0.350 | 0.350 | 0.163 | 0.000 | 0.163 | 0.163 | 0.163 | 1300 | 1.23836 | 1300 | 1.27040 |
| 0.450 | 0.450 | 0.138 | 0.000 | 0.138 | 0.138 | 0.138 | 1300 | 1.27825 | 1300 | 1.31011 |
| 0.550 | 0.550 | 0.113 | 0.000 | 0.113 | 0.113 | 0.113 | 1300 | 1.31918 | 1300 | 1.33287 |
| 0.650 | 0.650 | 0.088 | 0.000 | 0.088 | 0.088 | 0.088 | 1300 | 1.33329 | 1300 | 1.33300 |
| 0.750 | 0.750 | 0.063 | 0.000 | 0.063 | 0.063 | 0.063 | 1300 | 1.33330 | 1300 | 1.33314 |
| 0.850 | 0.850 | 0.038 | 0.000 | 0.038 | 0.038 | 0.038 | 1300 | 1.33332 | 1300 | 1.33348 |
| 0.950 | 0.950 | 0.013 | 0.000 | 0.013 | 0.013 | 0.013 | 1300 | 1.33338 | 1300 | 1.33426 |
| 0.050 | 0.050 | 0.000 | 0.238 | 0.238 | 0.238 | 0.238 | 1300 | 1.01417 | 1300 | 1.02621 |
| 0.150 | 0.150 | 0.000 | 0.213 | 0.213 | 0.213 | 0.213 | 1300 | 1.04162 | 1300 | 1.06158 |
| 0.250 | 0.250 | 0.000 | 0.188 | 0.188 | 0.188 | 0.188 | 1300 | 1.07376 | 1300 | 1.09705 |
| 0.350 | 0.350 | 0.000 | 0.163 | 0.163 | 0.163 | 0.163 | 1300 | 1.10451 | 1300 | 1.13666 |
| 0.450 | 0.450 | 0.000 | 0.138 | 0.138 | 0.138 | 0.138 | 1300 | 1.14312 | 1300 | 1.18610 |
| 0.550 | 0.550 | 0.000 | 0.113 | 0.113 | 0.113 | 0.113 | 1300 | 1.20935 | 1300 | 1.24886 |
| 0.650 | 0.650 | 0.000 | 0.088 | 0.088 | 0.088 | 0.088 | 1300 | 1.27439 | 1300 | 1.30933 |
| 0.750 | 0.750 | 0.000 | 0.063 | 0.063 | 0.063 | 0.063 | 1300 | 1.33329 | 1300 | 1.33298 |
| 0.850 | 0.850 | 0.000 | 0.038 | 0.038 | 0.038 | 0.038 | 1300 | 1.33330 | 1300 | 1.33321 |
| 0.950 | 0.950 | 0.000 | 0.013 | 0.013 | 0.013 | 0.013 | 1300 | 1.33337 | 1300 | 1.33405 |
| 0.050 | 0.050 | 0.190 | 0.190 | 0.190 | 0.190 | 0.190 | 1300 | 1.11600 | 1300 | 1.13303 |
| 0.150 | 0.150 | 0.170 | 0.170 | 0.170 | 0.170 | 0.170 | 1300 | 1.14022 | 1300 | 1.16486 |
| 0.250 | 0.250 | 0.150 | 0.150 | 0.150 | 0.150 | 0.150 | 1300 | 1.17323 | 1300 | 1.20243 |
| 0.350 | 0.350 | 0.130 | 0.130 | 0.130 | 0.130 | 0.130 | 1300 | 1.21265 | 1300 | 1.24275 |
| 0.450 | 0.450 | 0.110 | 0.110 | 0.110 | 0.110 | 0.110 | 1300 | 1.25320 | 1300 | 1.28305 |
| 0.550 | 0.550 | 0.090 | 0.090 | 0.090 | 0.090 | 0.090 | 1300 | 1.29440 | 1300 | 1.32304 |
| 0.650 | 0.650 | 0.070 | 0.070 | 0.070 | 0.070 | 0.070 | 1300 | 1.33329 | 1300 | 1.33299 |
| 0.750 | 0.750 | 0.050 | 0.050 | 0.050 | 0.050 | 0.050 | 1300 | 1.33329 | 1300 | 1.33311 |
| 0.850 | 0.850 | 0.030 | 0.030 | 0.030 | 0.030 | 0.030 | 1300 | 1.33332 | 1300 | 1.33345 |
| 0.950 | 0.950 | 0.010 | 0.010 | 0.010 | 0.010 | 0.010 | 1300 | 1.33338 | 1300 | 1.33423 |
| 0.050 | 0.050 | 0.000 | 0.000 | 0.000 | 0.000 | 0.950 | 1300 | 1.02093 | 1100 | 1.03509 |
| 0.150 | 0.150 | 0.000 | 0.000 | 0.000 | 0.000 | 0.850 | 1300 | 1.05577 | 1100 | 1.08744 |
| 0.250 | 0.250 | 0.000 | 0.000 | 0.000 | 0.000 | 0.750 | 1300 | 1.09949 | 1100 | 1.14352 |
| 0.350 | 0.350 | 0.000 | 0.000 | 0.000 | 0.000 | 0.650 | 1300 | 1.15168 | 1100 | 1.20319 |
| 0.450 | 0.450 | 0.000 | 0.000 | 0.000 | 0.000 | 0.550 | 1300 | 1.22628 | 1100 | 1.27552 |
| 0.550 | 0.550 | 0.000 | 0.000 | 0.000 | 0.000 | 0.450 | 1300 | 1.30088 | 1100 | 1.33326 |
| 0.650 | 0.650 | 0.000 | 0.000 | 0.000 | 0.000 | 0.350 | 1300 | 1.33328 | 1100 | 1.33327 |
| 0.750 | 0.750 | 0.000 | 0.000 | 0.000 | 0.000 | 0.250 | 1300 | 1.33329 | 1100 | 1.33329 |
| 0.850 | 0.850 | 0.000 | 0.000 | 0.000 | 0.000 | 0.150 | 1300 | 1.33330 | 1100 | 1.33349 |
| 0.950 | 0.950 | 0.000 | 0.000 | 0.000 | 0.000 | 0.050 | 1300 | 1.33335 | 1100 | 1.33492 |
| 0.050 | 0.050 | 0.475 | 0.000 | 0.000 | 0.000 | 0.475 | 1300 | 1.33328 | 1100 | 1.33328 |
| 0.150 | 0.150 | 0.425 | 0.000 | 0.000 | 0.000 | 0.425 | 1300 | 1.33328 | 1100 | 1.33328 |
| 0.250 | 0.250 | 0.375 | 0.000 | 0.000 | 0.000 | 0.375 | 1300 | 1.33328 | 1100 | 1.33327 |
| 0.350 | 0.350 | 0.325 | 0.000 | 0.000 | 0.000 | 0.325 | 1300 | 1.33328 | 1100 | 1.33327 |
| 0.450 | 0.450 | 0.275 | 0.000 | 0.000 | 0.000 | 0.275 | 1300 | 1.33329 | 1100 | 1.33328 |
| 0.550 | 0.550 | 0.225 | 0.000 | 0.000 | 0.000 | 0.225 | 1300 | 1.33329 | 1100 | 1.33330 |
| 0.650 | 0.650 | 0.175 | 0.000 | 0.000 | 0.000 | 0.175 | 1300 | 1.33330 | 1100 | 1.33340 |
| 0.750 | 0.750 | 0.125 | 0.000 | 0.000 | 0.000 | 0.125 | 1300 | 1.33331 | 1100 | 1.33371 |
| 0.850 | 0.850 | 0.075 | 0.000 | 0.000 | 0.000 | 0.075 | 1300 | 1.33334 | 1100 | 1.33445 |
| 0.950 | 0.950 | 0.025 | 0.000 | 0.000 | 0.000 | 0.025 | 1300 | 1.33340 | 1100 | 1.33571 |
| 0.050 | 0.050 | 0.000 | 0.475 | 0.000 | 0.000 | 0.475 | 1300 | 1.01425 | 1100 | 1.02453 |
| 0.150 | 0.150 | 0.000 | 0.425 | 0.000 | 0.000 | 0.425 | 1300 | 1.04119 | 1100 | 1.06281 |
| 0.250 | 0.250 | 0.000 | 0.375 | 0.000 | 0.000 | 0.375 | 1300 | 1.07727 | 1100 | 1.11016 |
| 0.350 | 0.350 | 0.000 | 0.325 | 0.000 | 0.000 | 0.325 | 1300 | 1.11350 | 1100 | 1.15771 |
| 0.450 | 0.450 | 0.000 | 0.275 | 0.000 | 0.000 | 0.275 | 1300 | 1.15588 | 1100 | 1.20777 |
| 0.550 | 0.550 | 0.000 | 0.225 | 0.000 | 0.000 | 0.225 | 1300 | 1.21955 | 1100 | 1.26525 |
| 0.650 | 0.650 | 0.000 | 0.175 | 0.000 | 0.000 | 0.175 | 1300 | 1.28118 | 1100 | 1.32076 |
| 0.750 | 0.750 | 0.000 | 0.125 | 0.000 | 0.000 | 0.125 | 1300 | 1.33329 | 1100 | 1.33335 |
| 0.850 | 0.850 | 0.000 | 0.075 | 0.000 | 0.000 | 0.075 | 1300 | 1.33330 | 1100 | 1.33380 |
| 0.950 | 0.950 | 0.000 | 0.025 | 0.000 | 0.000 | 0.025 | 1300 | 1.33336 | 1100 | 1.33527 |
| 0.050 | 0.050 | 0.000 | 0.000 | 0.475 | 0.000 | 0.475 | 1300 | 1.02346 | 1100 | 1.03646 |
| 0.150 | 0.150 | 0.000 | 0.000 | 0.425 | 0.000 | 0.425 | 1300 | 1.05059 | 1100 | 1.06845 |
| 0.250 | 0.250 | 0.000 | 0.000 | 0.375 | 0.000 | 0.375 | 1300 | 1.07869 | 1100 | 1.10909 |
| 0.350 | 0.350 | 0.000 | 0.000 | 0.325 | 0.000 | 0.325 | 1300 | 1.10564 | 1100 | 1.15283 |
| 0.450 | 0.450 | 0.000 | 0.000 | 0.275 | 0.000 | 0.275 | 1300 | 1.14376 | 1100 | 1.20329 |
| 0.550 | 0.550 | 0.000 | 0.000 | 0.225 | 0.000 | 0.225 | 1300 | 1.21211 | 1100 | 1.26602 |
| 0.650 | 0.650 | 0.000 | 0.000 | 0.175 | 0.000 | 0.175 | 1300 | 1.27998 | 1100 | 1.32834 |
| 0.750 | 0.750 | 0.000 | 0.000 | 0.125 | 0.000 | 0.125 | 1300 | 1.33329 | 1100 | 1.33337 |
| 0.850 | 0.850 | 0.000 | 0.000 | 0.075 | 0.000 | 0.075 | 1300 | 1.33330 | 1100 | 1.33386 |
| 0.950 | 0.950 | 0.000 | 0.000 | 0.025 | 0.000 | 0.025 | 1300 | 1.33336 | 1100 | 1.33529 |
| 0.050 | 0.050 | 0.000 | 0.000 | 0.000 | 0.475 | 0.475 | 1300 | 1.01439 | 1100 | 1.02347 |
| 0.150 | 0.150 | 0.000 | 0.000 | 0.000 | 0.425 | 0.425 | 1300 | 1.03827 | 1100 | 1.05941 |
| 0.250 | 0.250 | 0.000 | 0.000 | 0.000 | 0.375 | 0.375 | 1300 | 1.06974 | 1100 | 1.10512 |
| 0.350 | 0.350 | 0.000 | 0.000 | 0.000 | 0.325 | 0.325 | 1300 | 1.10257 | 1100 | 1.15272 |
| 0.450 | 0.450 | 0.000 | 0.000 | 0.000 | 0.275 | 0.275 | 1300 | 1.16466 | 1100 | 1.21161 |
| 0.550 | 0.550 | 0.000 | 0.000 | 0.000 | 0.225 | 0.225 | 1300 | 1.22789 | 1100 | 1.27024 |
| 0.650 | 0.650 | 0.000 | 0.000 | 0.000 | 0.175 | 0.175 | 1300 | 1.28882 | 1100 | 1.32656 |
| 0.750 | 0.750 | 0.000 | 0.000 | 0.000 | 0.125 | 0.125 | 1300 | 1.33329 | 1100 | 1.33333 |
| 0.850 | 0.850 | 0.000 | 0.000 | 0.000 | 0.075 | 0.075 | 1300 | 1.33330 | 1100 | 1.33378 |
| 0.950 | 0.950 | 0.000 | 0.000 | 0.000 | 0.025 | 0.025 | 1300 | 1.33336 | 1100 | 1.33527 |
| 0.050 | 0.050 | 0.317 | 0.317 | 0.000 | 0.000 | 0.317 | 1300 | 1.19769 | 1100 | 1.22220 |
| 0.150 | 0.150 | 0.283 | 0.283 | 0.000 | 0.000 | 0.283 | 1300 | 1.22254 | 1100 | 1.25379 |
| 0.250 | 0.250 | 0.250 | 0.250 | 0.000 | 0.000 | 0.250 | 1300 | 1.25487 | 1100 | 1.28876 |
| 0.350 | 0.350 | 0.217 | 0.217 | 0.000 | 0.000 | 0.217 | 1300 | 1.28817 | 1100 | 1.32184 |
| 0.450 | 0.450 | 0.183 | 0.183 | 0.000 | 0.000 | 0.183 | 1300 | 1.31946 | 1100 | 1.33326 |
| 0.550 | 0.550 | 0.150 | 0.150 | 0.000 | 0.000 | 0.150 | 1300 | 1.33329 | 1100 | 1.33331 |
| 0.650 | 0.650 | 0.117 | 0.117 | 0.000 | 0.000 | 0.117 | 1300 | 1.33329 | 1100 | 1.33344 |
| 0.750 | 0.750 | 0.083 | 0.083 | 0.000 | 0.000 | 0.083 | 1300 | 1.33331 | 1100 | 1.33379 |
| 0.850 | 0.850 | 0.050 | 0.050 | 0.000 | 0.000 | 0.050 | 1300 | 1.33333 | 1100 | 1.33450 |
| 0.950 | 0.950 | 0.017 | 0.017 | 0.000 | 0.000 | 0.017 | 1300 | 1.33339 | 1100 | 1.33570 |
| 0.050 | 0.050 | 0.317 | 0.000 | 0.317 | 0.000 | 0.317 | 1300 | 1.19797 | 1100 | 1.22217 |
| 0.150 | 0.150 | 0.283 | 0.000 | 0.283 | 0.000 | 0.283 | 1300 | 1.21958 | 1100 | 1.25007 |
| 0.250 | 0.250 | 0.250 | 0.000 | 0.250 | 0.000 | 0.250 | 1300 | 1.24656 | 1100 | 1.28151 |
| 0.350 | 0.350 | 0.217 | 0.000 | 0.217 | 0.000 | 0.217 | 1300 | 1.27791 | 1100 | 1.31466 |
| 0.450 | 0.450 | 0.183 | 0.000 | 0.183 | 0.000 | 0.183 | 1300 | 1.31069 | 1100 | 1.33326 |
| 0.550 | 0.550 | 0.150 | 0.000 | 0.150 | 0.000 | 0.150 | 1300 | 1.33328 | 1100 | 1.33330 |
| 0.650 | 0.650 | 0.117 | 0.000 | 0.117 | 0.000 | 0.117 | 1300 | 1.33329 | 1100 | 1.33343 |
| 0.750 | 0.750 | 0.083 | 0.000 | 0.083 | 0.000 | 0.083 | 1300 | 1.33330 | 1100 | 1.33378 |
| 0.850 | 0.850 | 0.050 | 0.000 | 0.050 | 0.000 | 0.050 | 1300 | 1.33333 | 1100 | 1.33450 |
| 0.950 | 0.950 | 0.017 | 0.000 | 0.017 | 0.000 | 0.017 | 1300 | 1.33339 | 1100 | 1.33570 |
| 0.050 | 0.050 | 0.317 | 0.000 | 0.000 | 0.317 | 0.317 | 1300 | 1.18564 | 1100 | 1.20626 |
| 0.150 | 0.150 | 0.283 | 0.000 | 0.000 | 0.283 | 0.283 | 1300 | 1.20654 | 1100 | 1.23204 |
| 0.250 | 0.250 | 0.250 | 0.000 | 0.000 | 0.250 | 0.250 | 1300 | 1.23585 | 1100 | 1.26363 |
| 0.350 | 0.350 | 0.217 | 0.000 | 0.000 | 0.217 | 0.217 | 1300 | 1.26856 | 1100 | 1.29759 |
| 0.450 | 0.450 | 0.183 | 0.000 | 0.000 | 0.183 | 0.183 | 1300 | 1.30179 | 1100 | 1.33158 |
| 0.550 | 0.550 | 0.150 | 0.000 | 0.000 | 0.150 | 0.150 | 1300 | 1.33329 | 1100 | 1.33330 |
| 0.650 | 0.650 | 0.117 | 0.000 | 0.000 | 0.117 | 0.117 | 1300 | 1.33329 | 1100 | 1.33343 |
| 0.750 | 0.750 | 0.083 | 0.000 | 0.000 | 0.083 | 0.083 | 1300 | 1.33330 | 1100 | 1.33376 |
| 0.850 | 0.850 | 0.050 | 0.000 | 0.000 | 0.050 | 0.050 | 1300 | 1.33333 | 1100 | 1.33448 |
| 0.950 | 0.950 | 0.017 | 0.000 | 0.000 | 0.017 | 0.017 | 1300 | 1.33339 | 1100 | 1.33570 |
| 0.050 | 0.050 | 0.000 | 0.317 | 0.317 | 0.000 | 0.317 | 1300 | 1.01670 | 1100 | 1.02742 |
| 0.150 | 0.150 | 0.000 | 0.283 | 0.283 | 0.000 | 0.283 | 1300 | 1.04435 | 1100 | 1.06219 |
| 0.250 | 0.250 | 0.000 | 0.250 | 0.250 | 0.000 | 0.250 | 1300 | 1.07574 | 1100 | 1.10501 |
| 0.350 | 0.350 | 0.000 | 0.217 | 0.217 | 0.000 | 0.217 | 1300 | 1.10602 | 1100 | 1.15079 |
| 0.450 | 0.450 | 0.000 | 0.183 | 0.183 | 0.000 | 0.183 | 1300 | 1.13627 | 1100 | 1.19846 |
| 0.550 | 0.550 | 0.000 | 0.150 | 0.150 | 0.000 | 0.150 | 1300 | 1.20462 | 1100 | 1.26050 |
| 0.650 | 0.650 | 0.000 | 0.117 | 0.117 | 0.000 | 0.117 | 1300 | 1.27226 | 1100 | 1.32158 |
| 0.750 | 0.750 | 0.000 | 0.083 | 0.083 | 0.000 | 0.083 | 1300 | 1.33329 | 1100 | 1.33336 |
| 0.850 | 0.850 | 0.000 | 0.050 | 0.050 | 0.000 | 0.050 | 1300 | 1.33330 | 1100 | 1.33392 |
| 0.950 | 0.950 | 0.000 | 0.017 | 0.017 | 0.000 | 0.017 | 1300 | 1.33337 | 1100 | 1.33539 |
| 0.050 | 0.050 | 0.000 | 0.317 | 0.000 | 0.317 | 0.317 | 1300 | 1.01219 | 1100 | 1.02114 |
| 0.150 | 0.150 | 0.000 | 0.283 | 0.000 | 0.283 | 0.283 | 1300 | 1.03796 | 1100 | 1.05817 |
| 0.250 | 0.250 | 0.000 | 0.250 | 0.000 | 0.250 | 0.250 | 1300 | 1.07260 | 1100 | 1.10478 |
| 0.350 | 0.350 | 0.000 | 0.217 | 0.000 | 0.217 | 0.217 | 1300 | 1.10724 | 1100 | 1.15196 |
| 0.450 | 0.450 | 0.000 | 0.183 | 0.000 | 0.183 | 0.183 | 1300 | 1.15216 | 1100 | 1.20296 |
| 0.550 | 0.550 | 0.000 | 0.150 | 0.000 | 0.150 | 0.150 | 1300 | 1.21391 | 1100 | 1.25894 |
| 0.650 | 0.650 | 0.000 | 0.117 | 0.000 | 0.117 | 0.117 | 1300 | 1.27378 | 1100 | 1.31308 |
| 0.750 | 0.750 | 0.000 | 0.083 | 0.000 | 0.083 | 0.083 | 1300 | 1.33112 | 1100 | 1.33332 |
| 0.850 | 0.850 | 0.000 | 0.050 | 0.000 | 0.050 | 0.050 | 1300 | 1.33330 | 1100 | 1.33391 |
| 0.950 | 0.950 | 0.000 | 0.017 | 0.000 | 0.017 | 0.017 | 1300 | 1.33337 | 1100 | 1.33538 |
| 0.050 | 0.050 | 0.000 | 0.000 | 0.317 | 0.317 | 0.317 | 1300 | 1.01671 | 1100 | 1.02669 |
| 0.150 | 0.150 | 0.000 | 0.000 | 0.283 | 0.283 | 0.283 | 1300 | 1.04203 | 1100 | 1.06049 |
| 0.250 | 0.250 | 0.000 | 0.000 | 0.250 | 0.250 | 0.250 | 1300 | 1.07083 | 1100 | 1.10517 |
| 0.350 | 0.350 | 0.000 | 0.000 | 0.217 | 0.217 | 0.217 | 1300 | 1.09947 | 1100 | 1.15225 |
| 0.450 | 0.450 | 0.000 | 0.000 | 0.183 | 0.183 | 0.183 | 1300 | 1.15065 | 1100 | 1.20899 |
| 0.550 | 0.550 | 0.000 | 0.000 | 0.150 | 0.150 | 0.150 | 1300 | 1.21920 | 1100 | 1.27193 |
| 0.650 | 0.650 | 0.000 | 0.000 | 0.117 | 0.117 | 0.117 | 1300 | 1.28729 | 1100 | 1.33322 |
| 0.750 | 0.750 | 0.000 | 0.000 | 0.083 | 0.083 | 0.083 | 1300 | 1.33329 | 1100 | 1.33336 |
| 0.850 | 0.850 | 0.000 | 0.000 | 0.050 | 0.050 | 0.050 | 1300 | 1.33330 | 1100 | 1.33392 |
| 0.950 | 0.950 | 0.000 | 0.000 | 0.017 | 0.017 | 0.017 | 1300 | 1.33337 | 1100 | 1.33539 |
| 0.050 | 0.050 | 0.238 | 0.238 | 0.238 | 0.000 | 0.238 | 1300 | 1.14670 | 1100 | 1.16612 |
| 0.150 | 0.150 | 0.213 | 0.213 | 0.213 | 0.000 | 0.213 | 1300 | 1.16929 | 1100 | 1.19761 |
| 0.250 | 0.250 | 0.188 | 0.188 | 0.188 | 0.000 | 0.188 | 1300 | 1.19942 | 1100 | 1.23459 |
| 0.350 | 0.350 | 0.163 | 0.163 | 0.163 | 0.000 | 0.163 | 1300 | 1.23686 | 1100 | 1.27399 |
| 0.450 | 0.450 | 0.138 | 0.138 | 0.138 | 0.000 | 0.138 | 1300 | 1.27525 | 1100 | 1.31292 |
| 0.550 | 0.550 | 0.113 | 0.113 | 0.113 | 0.000 | 0.113 | 1300 | 1.31388 | 1100 | 1.33326 |
| 0.650 | 0.650 | 0.088 | 0.088 | 0.088 | 0.000 | 0.088 | 1300 | 1.33329 | 1100 | 1.33336 |
| 0.750 | 0.750 | 0.063 | 0.063 | 0.063 | 0.000 | 0.063 | 1300 | 1.33330 | 1100 | 1.33370 |
| 0.850 | 0.850 | 0.038 | 0.038 | 0.038 | 0.000 | 0.038 | 1300 | 1.33332 | 1100 | 1.33444 |
| 0.950 | 0.950 | 0.013 | 0.013 | 0.013 | 0.000 | 0.013 | 1300 | 1.33339 | 1100 | 1.33568 |
| 0.050 | 0.050 | 0.238 | 0.238 | 0.000 | 0.238 | 0.238 | 1300 | 1.14148 | 1100 | 1.15882 |
| 0.150 | 0.150 | 0.213 | 0.213 | 0.000 | 0.213 | 0.213 | 1300 | 1.16499 | 1100 | 1.19106 |
| 0.250 | 0.250 | 0.188 | 0.188 | 0.000 | 0.188 | 0.188 | 1300 | 1.19895 | 1100 | 1.22909 |
| 0.350 | 0.350 | 0.163 | 0.163 | 0.000 | 0.163 | 0.163 | 1300 | 1.23625 | 1100 | 1.26720 |
| 0.450 | 0.450 | 0.138 | 0.138 | 0.000 | 0.138 | 0.138 | 1300 | 1.27281 | 1100 | 1.30382 |
| 0.550 | 0.550 | 0.113 | 0.113 | 0.000 | 0.113 | 0.113 | 1300 | 1.30864 | 1100 | 1.33324 |
| 0.650 | 0.650 | 0.088 | 0.088 | 0.000 | 0.088 | 0.088 | 1300 | 1.33329 | 1100 | 1.33338 |
| 0.750 | 0.750 | 0.063 | 0.063 | 0.000 | 0.063 | 0.063 | 1300 | 1.33330 | 1100 | 1.33373 |
| 0.850 | 0.850 | 0.038 | 0.038 | 0.000 | 0.038 | 0.038 | 1300 | 1.33333 | 1100 | 1.33446 |
| 0.950 | 0.950 | 0.013 | 0.013 | 0.000 | 0.013 | 0.013 | 1300 | 1.33339 | 1100 | 1.33568 |
| 0.050 | 0.050 | 0.238 | 0.000 | 0.238 | 0.238 | 0.238 | 1300 | 1.14317 | 1100 | 1.16119 |
| 0.150 | 0.150 | 0.213 | 0.000 | 0.213 | 0.213 | 0.213 | 1300 | 1.16721 | 1100 | 1.19509 |
| 0.250 | 0.250 | 0.188 | 0.000 | 0.188 | 0.188 | 0.188 | 1300 | 1.20012 | 1100 | 1.23433 |
| 0.350 | 0.350 | 0.163 | 0.000 | 0.163 | 0.163 | 0.163 | 1300 | 1.23836 | 1100 | 1.27537 |
| 0.450 | 0.450 | 0.138 | 0.000 | 0.138 | 0.138 | 0.138 | 1300 | 1.27825 | 1100 | 1.31651 |
| 0.550 | 0.550 | 0.113 | 0.000 | 0.113 | 0.113 | 0.113 | 1300 | 1.31918 | 1100 | 1.33325 |
| 0.650 | 0.650 | 0.088 | 0.000 | 0.088 | 0.088 | 0.088 | 1300 | 1.33329 | 1100 | 1.33336 |
| 0.750 | 0.750 | 0.063 | 0.000 | 0.063 | 0.063 | 0.063 | 1300 | 1.33330 | 1100 | 1.33368 |
| 0.850 | 0.850 | 0.038 | 0.000 | 0.038 | 0.038 | 0.038 | 1300 | 1.33332 | 1100 | 1.33443 |
| 0.950 | 0.950 | 0.013 | 0.000 | 0.013 | 0.013 | 0.013 | 1300 | 1.33338 | 1100 | 1.33568 |
| 0.050 | 0.050 | 0.000 | 0.238 | 0.238 | 0.238 | 0.238 | 1300 | 1.01417 | 1100 | 1.02368 |
| 0.150 | 0.150 | 0.000 | 0.213 | 0.213 | 0.213 | 0.213 | 1300 | 1.04162 | 1100 | 1.05877 |
| 0.250 | 0.250 | 0.000 | 0.188 | 0.188 | 0.188 | 0.188 | 1300 | 1.07376 | 1100 | 1.10449 |
| 0.350 | 0.350 | 0.000 | 0.163 | 0.163 | 0.163 | 0.163 | 1300 | 1.10451 | 1100 | 1.15128 |
| 0.450 | 0.450 | 0.000 | 0.138 | 0.138 | 0.138 | 0.138 | 1300 | 1.14312 | 1100 | 1.20215 |
| 0.550 | 0.550 | 0.000 | 0.113 | 0.113 | 0.113 | 0.113 | 1300 | 1.20935 | 1100 | 1.26189 |
| 0.650 | 0.650 | 0.000 | 0.088 | 0.088 | 0.088 | 0.088 | 1300 | 1.27439 | 1100 | 1.31965 |
| 0.750 | 0.750 | 0.000 | 0.063 | 0.063 | 0.063 | 0.063 | 1300 | 1.33329 | 1100 | 1.33336 |
| 0.850 | 0.850 | 0.000 | 0.038 | 0.038 | 0.038 | 0.038 | 1300 | 1.33330 | 1100 | 1.33396 |
| 0.950 | 0.950 | 0.000 | 0.013 | 0.013 | 0.013 | 0.013 | 1300 | 1.33337 | 1100 | 1.33544 |
| 0.050 | 0.050 | 0.190 | 0.190 | 0.190 | 0.190 | 0.190 | 1300 | 1.11600 | 1100 | 1.13200 |
| 0.150 | 0.150 | 0.170 | 0.170 | 0.170 | 0.170 | 0.170 | 1300 | 1.14022 | 1100 | 1.16641 |
| 0.250 | 0.250 | 0.150 | 0.150 | 0.150 | 0.150 | 0.150 | 1300 | 1.17323 | 1100 | 1.20634 |
| 0.350 | 0.350 | 0.130 | 0.130 | 0.130 | 0.130 | 0.130 | 1300 | 1.21265 | 1100 | 1.24801 |
| 0.450 | 0.450 | 0.110 | 0.110 | 0.110 | 0.110 | 0.110 | 1300 | 1.25320 | 1100 | 1.28923 |
| 0.550 | 0.550 | 0.090 | 0.090 | 0.090 | 0.090 | 0.090 | 1300 | 1.29440 | 1100 | 1.32955 |
| 0.650 | 0.650 | 0.070 | 0.070 | 0.070 | 0.070 | 0.070 | 1300 | 1.33329 | 1100 | 1.33332 |
| 0.750 | 0.750 | 0.050 | 0.050 | 0.050 | 0.050 | 0.050 | 1300 | 1.33329 | 1100 | 1.33363 |
| 0.850 | 0.850 | 0.030 | 0.030 | 0.030 | 0.030 | 0.030 | 1300 | 1.33332 | 1100 | 1.33439 |
| 0.950 | 0.950 | 0.010 | 0.010 | 0.010 | 0.010 | 0.010 | 1300 | 1.33338 | 1100 | 1.33567 |
| 0.050 | 0.050 | 0.000 | 0.000 | 0.000 | 0.000 | 0.950 | 1300 | 1.02093 | 800 | 1.02970 |
| 0.150 | 0.150 | 0.000 | 0.000 | 0.000 | 0.000 | 0.850 | 1300 | 1.05577 | 800 | 1.07917 |
| 0.250 | 0.250 | 0.000 | 0.000 | 0.000 | 0.000 | 0.750 | 1300 | 1.09949 | 800 | 1.13143 |
| 0.350 | 0.350 | 0.000 | 0.000 | 0.000 | 0.000 | 0.650 | 1300 | 1.15168 | 800 | 1.18657 |
| 0.450 | 0.450 | 0.000 | 0.000 | 0.000 | 0.000 | 0.550 | 1300 | 1.22628 | 800 | 1.25368 |
| 0.550 | 0.550 | 0.000 | 0.000 | 0.000 | 0.000 | 0.450 | 1300 | 1.30088 | 800 | 1.32356 |
| 0.650 | 0.650 | 0.000 | 0.000 | 0.000 | 0.000 | 0.350 | 1300 | 1.33328 | 800 | 1.33328 |
| 0.750 | 0.750 | 0.000 | 0.000 | 0.000 | 0.000 | 0.250 | 1300 | 1.33329 | 800 | 1.33335 |
| 0.850 | 0.850 | 0.000 | 0.000 | 0.000 | 0.000 | 0.150 | 1300 | 1.33330 | 800 | 1.33361 |
| 0.950 | 0.950 | 0.000 | 0.000 | 0.000 | 0.000 | 0.050 | 1300 | 1.33335 | 800 | 1.33413 |
| 0.050 | 0.050 | 0.475 | 0.000 | 0.000 | 0.000 | 0.475 | 1300 | 1.33328 | 800 | 1.33328 |
| 0.150 | 0.150 | 0.425 | 0.000 | 0.000 | 0.000 | 0.425 | 1300 | 1.33328 | 800 | 1.33328 |
| 0.250 | 0.250 | 0.375 | 0.000 | 0.000 | 0.000 | 0.375 | 1300 | 1.33328 | 800 | 1.33328 |
| 0.350 | 0.350 | 0.325 | 0.000 | 0.000 | 0.000 | 0.325 | 1300 | 1.33328 | 800 | 1.33331 |
| 0.450 | 0.450 | 0.275 | 0.000 | 0.000 | 0.000 | 0.275 | 1300 | 1.33329 | 800 | 1.33341 |
| 0.550 | 0.550 | 0.225 | 0.000 | 0.000 | 0.000 | 0.225 | 1300 | 1.33329 | 800 | 1.33361 |
| 0.650 | 0.650 | 0.175 | 0.000 | 0.000 | 0.000 | 0.175 | 1300 | 1.33330 | 800 | 1.33391 |
| 0.750 | 0.750 | 0.125 | 0.000 | 0.000 | 0.000 | 0.125 | 1300 | 1.33331 | 800 | 1.33403 |
| 0.850 | 0.850 | 0.075 | 0.000 | 0.000 | 0.000 | 0.075 | 1300 | 1.33334 | 800 | 1.33402 |
| 0.950 | 0.950 | 0.025 | 0.000 | 0.000 | 0.000 | 0.025 | 1300 | 1.33340 | 800 | 1.33435 |
| 0.050 | 0.050 | 0.000 | 0.475 | 0.000 | 0.000 | 0.475 | 1300 | 1.01425 | 800 | 1.02094 |
| 0.150 | 0.150 | 0.000 | 0.425 | 0.000 | 0.000 | 0.425 | 1300 | 1.04119 | 800 | 1.06443 |
| 0.250 | 0.250 | 0.000 | 0.375 | 0.000 | 0.000 | 0.375 | 1300 | 1.07727 | 800 | 1.11178 |
| 0.350 | 0.350 | 0.000 | 0.325 | 0.000 | 0.000 | 0.325 | 1300 | 1.11350 | 800 | 1.15917 |
| 0.450 | 0.450 | 0.000 | 0.275 | 0.000 | 0.000 | 0.275 | 1300 | 1.15588 | 800 | 1.20809 |
| 0.550 | 0.550 | 0.000 | 0.225 | 0.000 | 0.000 | 0.225 | 1300 | 1.21955 | 800 | 1.26261 |
| 0.650 | 0.650 | 0.000 | 0.175 | 0.000 | 0.000 | 0.175 | 1300 | 1.28118 | 800 | 1.31740 |
| 0.750 | 0.750 | 0.000 | 0.125 | 0.000 | 0.000 | 0.125 | 1300 | 1.33329 | 800 | 1.33337 |
| 0.850 | 0.850 | 0.000 | 0.075 | 0.000 | 0.000 | 0.075 | 1300 | 1.33330 | 800 | 1.33357 |
| 0.950 | 0.950 | 0.000 | 0.025 | 0.000 | 0.000 | 0.025 | 1300 | 1.33336 | 800 | 1.33413 |
| 0.050 | 0.050 | 0.000 | 0.000 | 0.475 | 0.000 | 0.475 | 1300 | 1.02346 | 800 | 1.02776 |
| 0.150 | 0.150 | 0.000 | 0.000 | 0.425 | 0.000 | 0.425 | 1300 | 1.05059 | 800 | 1.06973 |
| 0.250 | 0.250 | 0.000 | 0.000 | 0.375 | 0.000 | 0.375 | 1300 | 1.07869 | 800 | 1.11606 |
| 0.350 | 0.350 | 0.000 | 0.000 | 0.325 | 0.000 | 0.325 | 1300 | 1.10564 | 800 | 1.16373 |
| 0.450 | 0.450 | 0.000 | 0.000 | 0.275 | 0.000 | 0.275 | 1300 | 1.14376 | 800 | 1.21437 |
| 0.550 | 0.550 | 0.000 | 0.000 | 0.225 | 0.000 | 0.225 | 1300 | 1.21211 | 800 | 1.27084 |
| 0.650 | 0.650 | 0.000 | 0.000 | 0.175 | 0.000 | 0.175 | 1300 | 1.27998 | 800 | 1.32796 |
| 0.750 | 0.750 | 0.000 | 0.000 | 0.125 | 0.000 | 0.125 | 1300 | 1.33329 | 800 | 1.33341 |
| 0.850 | 0.850 | 0.000 | 0.000 | 0.075 | 0.000 | 0.075 | 1300 | 1.33330 | 800 | 1.33358 |
| 0.950 | 0.950 | 0.000 | 0.000 | 0.025 | 0.000 | 0.025 | 1300 | 1.33336 | 800 | 1.33412 |
| 0.050 | 0.050 | 0.000 | 0.000 | 0.000 | 0.475 | 0.475 | 1300 | 1.01439 | 800 | 1.02006 |
| 0.150 | 0.150 | 0.000 | 0.000 | 0.000 | 0.425 | 0.425 | 1300 | 1.03827 | 800 | 1.06335 |
| 0.250 | 0.250 | 0.000 | 0.000 | 0.000 | 0.375 | 0.375 | 1300 | 1.06974 | 800 | 1.11145 |
| 0.350 | 0.350 | 0.000 | 0.000 | 0.000 | 0.325 | 0.325 | 1300 | 1.10257 | 800 | 1.16067 |
| 0.450 | 0.450 | 0.000 | 0.000 | 0.000 | 0.275 | 0.275 | 1300 | 1.16466 | 800 | 1.21591 |
| 0.550 | 0.550 | 0.000 | 0.000 | 0.000 | 0.225 | 0.225 | 1300 | 1.22789 | 800 | 1.27156 |
| 0.650 | 0.650 | 0.000 | 0.000 | 0.000 | 0.175 | 0.175 | 1300 | 1.28882 | 800 | 1.32646 |
| 0.750 | 0.750 | 0.000 | 0.000 | 0.000 | 0.125 | 0.125 | 1300 | 1.33329 | 800 | 1.33338 |
| 0.850 | 0.850 | 0.000 | 0.000 | 0.000 | 0.075 | 0.075 | 1300 | 1.33330 | 800 | 1.33356 |
| 0.950 | 0.950 | 0.000 | 0.000 | 0.000 | 0.025 | 0.025 | 1300 | 1.33336 | 800 | 1.33413 |
| 0.050 | 0.050 | 0.317 | 0.317 | 0.000 | 0.000 | 0.317 | 1300 | 1.19769 | 800 | 1.21141 |
| 0.150 | 0.150 | 0.283 | 0.283 | 0.000 | 0.000 | 0.283 | 1300 | 1.22254 | 800 | 1.24202 |
| 0.250 | 0.250 | 0.250 | 0.250 | 0.000 | 0.000 | 0.250 | 1300 | 1.25487 | 800 | 1.27497 |
| 0.350 | 0.350 | 0.217 | 0.217 | 0.000 | 0.000 | 0.217 | 1300 | 1.28817 | 800 | 1.30839 |
| 0.450 | 0.450 | 0.183 | 0.183 | 0.000 | 0.000 | 0.183 | 1300 | 1.31946 | 800 | 1.33328 |
| 0.550 | 0.550 | 0.150 | 0.150 | 0.000 | 0.000 | 0.150 | 1300 | 1.33329 | 800 | 1.33332 |
| 0.650 | 0.650 | 0.117 | 0.117 | 0.000 | 0.000 | 0.117 | 1300 | 1.33329 | 800 | 1.33339 |
| 0.750 | 0.750 | 0.083 | 0.083 | 0.000 | 0.000 | 0.083 | 1300 | 1.33331 | 800 | 1.33355 |
| 0.850 | 0.850 | 0.050 | 0.050 | 0.000 | 0.000 | 0.050 | 1300 | 1.33333 | 800 | 1.33384 |
| 0.950 | 0.950 | 0.017 | 0.017 | 0.000 | 0.000 | 0.017 | 1300 | 1.33339 | 800 | 1.33427 |
| 0.050 | 0.050 | 0.317 | 0.000 | 0.317 | 0.000 | 0.317 | 1300 | 1.19797 | 800 | 1.21289 |
| 0.150 | 0.150 | 0.283 | 0.000 | 0.283 | 0.000 | 0.283 | 1300 | 1.21958 | 800 | 1.24344 |
| 0.250 | 0.250 | 0.250 | 0.000 | 0.250 | 0.000 | 0.250 | 1300 | 1.24656 | 800 | 1.27682 |
| 0.350 | 0.350 | 0.217 | 0.000 | 0.217 | 0.000 | 0.217 | 1300 | 1.27791 | 800 | 1.31136 |
| 0.450 | 0.450 | 0.183 | 0.000 | 0.183 | 0.000 | 0.183 | 1300 | 1.31069 | 800 | 1.33328 |
| 0.550 | 0.550 | 0.150 | 0.000 | 0.150 | 0.000 | 0.150 | 1300 | 1.33328 | 800 | 1.33334 |
| 0.650 | 0.650 | 0.117 | 0.000 | 0.117 | 0.000 | 0.117 | 1300 | 1.33329 | 800 | 1.33342 |
| 0.750 | 0.750 | 0.083 | 0.000 | 0.083 | 0.000 | 0.083 | 1300 | 1.33330 | 800 | 1.33354 |
| 0.850 | 0.850 | 0.050 | 0.000 | 0.050 | 0.000 | 0.050 | 1300 | 1.33333 | 800 | 1.33382 |
| 0.950 | 0.950 | 0.017 | 0.000 | 0.017 | 0.000 | 0.017 | 1300 | 1.33339 | 800 | 1.33427 |
| 0.050 | 0.050 | 0.317 | 0.000 | 0.000 | 0.317 | 0.317 | 1300 | 1.18564 | 800 | 1.19946 |
| 0.150 | 0.150 | 0.283 | 0.000 | 0.000 | 0.283 | 0.283 | 1300 | 1.20654 | 800 | 1.22616 |
| 0.250 | 0.250 | 0.250 | 0.000 | 0.000 | 0.250 | 0.250 | 1300 | 1.23585 | 800 | 1.25967 |
| 0.350 | 0.350 | 0.217 | 0.000 | 0.000 | 0.217 | 0.217 | 1300 | 1.26856 | 800 | 1.29591 |
| 0.450 | 0.450 | 0.183 | 0.000 | 0.000 | 0.183 | 0.183 | 1300 | 1.30179 | 800 | 1.33238 |
| 0.550 | 0.550 | 0.150 | 0.000 | 0.000 | 0.150 | 0.150 | 1300 | 1.33329 | 800 | 1.33334 |
| 0.650 | 0.650 | 0.117 | 0.000 | 0.000 | 0.117 | 0.117 | 1300 | 1.33329 | 800 | 1.33341 |
| 0.750 | 0.750 | 0.083 | 0.000 | 0.000 | 0.083 | 0.083 | 1300 | 1.33330 | 800 | 1.33352 |
| 0.850 | 0.850 | 0.050 | 0.000 | 0.000 | 0.050 | 0.050 | 1300 | 1.33333 | 800 | 1.33383 |
| 0.950 | 0.950 | 0.017 | 0.000 | 0.000 | 0.017 | 0.017 | 1300 | 1.33339 | 800 | 1.33427 |
| 0.050 | 0.050 | 0.000 | 0.317 | 0.317 | 0.000 | 0.317 | 1300 | 1.01670 | 800 | 1.02233 |
| 0.150 | 0.150 | 0.000 | 0.283 | 0.283 | 0.000 | 0.283 | 1300 | 1.04435 | 800 | 1.06783 |
| 0.250 | 0.250 | 0.000 | 0.250 | 0.250 | 0.000 | 0.250 | 1300 | 1.07574 | 800 | 1.11644 |
| 0.350 | 0.350 | 0.000 | 0.217 | 0.217 | 0.000 | 0.217 | 1300 | 1.10602 | 800 | 1.16578 |
| 0.450 | 0.450 | 0.000 | 0.183 | 0.183 | 0.000 | 0.183 | 1300 | 1.13627 | 800 | 1.21588 |
| 0.550 | 0.550 | 0.000 | 0.150 | 0.150 | 0.000 | 0.150 | 1300 | 1.20462 | 800 | 1.27264 |
| 0.650 | 0.650 | 0.000 | 0.117 | 0.117 | 0.000 | 0.117 | 1300 | 1.27226 | 800 | 1.32934 |
| 0.750 | 0.750 | 0.000 | 0.083 | 0.083 | 0.000 | 0.083 | 1300 | 1.33329 | 800 | 1.33341 |
| 0.850 | 0.850 | 0.000 | 0.050 | 0.050 | 0.000 | 0.050 | 1300 | 1.33330 | 800 | 1.33362 |
| 0.950 | 0.950 | 0.000 | 0.017 | 0.017 | 0.000 | 0.017 | 1300 | 1.33337 | 800 | 1.33413 |
| 0.050 | 0.050 | 0.000 | 0.317 | 0.000 | 0.317 | 0.317 | 1300 | 1.01219 | 800 | 1.01938 |
| 0.150 | 0.150 | 0.000 | 0.283 | 0.000 | 0.283 | 0.283 | 1300 | 1.03796 | 800 | 1.06391 |
| 0.250 | 0.250 | 0.000 | 0.250 | 0.000 | 0.250 | 0.250 | 1300 | 1.07260 | 800 | 1.11201 |
| 0.350 | 0.350 | 0.000 | 0.217 | 0.000 | 0.217 | 0.217 | 1300 | 1.10724 | 800 | 1.16025 |
| 0.450 | 0.450 | 0.000 | 0.183 | 0.000 | 0.183 | 0.183 | 1300 | 1.15216 | 800 | 1.21047 |
| 0.550 | 0.550 | 0.000 | 0.150 | 0.000 | 0.150 | 0.150 | 1300 | 1.21391 | 800 | 1.26397 |
| 0.650 | 0.650 | 0.000 | 0.117 | 0.000 | 0.117 | 0.117 | 1300 | 1.27378 | 800 | 1.31708 |
| 0.750 | 0.750 | 0.000 | 0.083 | 0.000 | 0.083 | 0.083 | 1300 | 1.33112 | 800 | 1.33329 |
| 0.850 | 0.850 | 0.000 | 0.050 | 0.000 | 0.050 | 0.050 | 1300 | 1.33330 | 800 | 1.33361 |
| 0.950 | 0.950 | 0.000 | 0.017 | 0.000 | 0.017 | 0.017 | 1300 | 1.33337 | 800 | 1.33413 |
| 0.050 | 0.050 | 0.000 | 0.000 | 0.317 | 0.317 | 0.317 | 1300 | 1.01671 | 800 | 1.02255 |
| 0.150 | 0.150 | 0.000 | 0.000 | 0.283 | 0.283 | 0.283 | 1300 | 1.04203 | 800 | 1.06801 |
| 0.250 | 0.250 | 0.000 | 0.000 | 0.250 | 0.250 | 0.250 | 1300 | 1.07083 | 800 | 1.11722 |
| 0.350 | 0.350 | 0.000 | 0.000 | 0.217 | 0.217 | 0.217 | 1300 | 1.09947 | 800 | 1.16772 |
| 0.450 | 0.450 | 0.000 | 0.000 | 0.183 | 0.183 | 0.183 | 1300 | 1.15065 | 800 | 1.22263 |
| 0.550 | 0.550 | 0.000 | 0.000 | 0.150 | 0.150 | 0.150 | 1300 | 1.21920 | 800 | 1.28078 |
| 0.650 | 0.650 | 0.000 | 0.000 | 0.117 | 0.117 | 0.117 | 1300 | 1.28729 | 800 | 1.33328 |
| 0.750 | 0.750 | 0.000 | 0.000 | 0.083 | 0.083 | 0.083 | 1300 | 1.33329 | 800 | 1.33342 |
| 0.850 | 0.850 | 0.000 | 0.000 | 0.050 | 0.050 | 0.050 | 1300 | 1.33330 | 800 | 1.33363 |
| 0.950 | 0.950 | 0.000 | 0.000 | 0.017 | 0.017 | 0.017 | 1300 | 1.33337 | 800 | 1.33413 |
| 0.050 | 0.050 | 0.238 | 0.238 | 0.238 | 0.000 | 0.238 | 1300 | 1.14670 | 800 | 1.16032 |
| 0.150 | 0.150 | 0.213 | 0.213 | 0.213 | 0.000 | 0.213 | 1300 | 1.16929 | 800 | 1.19527 |
| 0.250 | 0.250 | 0.188 | 0.188 | 0.188 | 0.000 | 0.188 | 1300 | 1.19942 | 800 | 1.23339 |
| 0.350 | 0.350 | 0.163 | 0.163 | 0.163 | 0.000 | 0.163 | 1300 | 1.23686 | 800 | 1.27436 |
| 0.450 | 0.450 | 0.138 | 0.138 | 0.138 | 0.000 | 0.138 | 1300 | 1.27525 | 800 | 1.31536 |
| 0.550 | 0.550 | 0.113 | 0.113 | 0.113 | 0.000 | 0.113 | 1300 | 1.31388 | 800 | 1.33328 |
| 0.650 | 0.650 | 0.088 | 0.088 | 0.088 | 0.000 | 0.088 | 1300 | 1.33329 | 800 | 1.33340 |
| 0.750 | 0.750 | 0.063 | 0.063 | 0.063 | 0.000 | 0.063 | 1300 | 1.33330 | 800 | 1.33354 |
| 0.850 | 0.850 | 0.038 | 0.038 | 0.038 | 0.000 | 0.038 | 1300 | 1.33332 | 800 | 1.33378 |
| 0.950 | 0.950 | 0.013 | 0.013 | 0.013 | 0.000 | 0.013 | 1300 | 1.33339 | 800 | 1.33424 |
| 0.050 | 0.050 | 0.238 | 0.238 | 0.000 | 0.238 | 0.238 | 1300 | 1.14148 | 800 | 1.15355 |
| 0.150 | 0.150 | 0.213 | 0.213 | 0.000 | 0.213 | 0.213 | 1300 | 1.16499 | 800 | 1.18737 |
| 0.250 | 0.250 | 0.188 | 0.188 | 0.000 | 0.188 | 0.188 | 1300 | 1.19895 | 800 | 1.22554 |
| 0.350 | 0.350 | 0.163 | 0.163 | 0.000 | 0.163 | 0.163 | 1300 | 1.23625 | 800 | 1.26463 |
| 0.450 | 0.450 | 0.138 | 0.138 | 0.000 | 0.138 | 0.138 | 1300 | 1.27281 | 800 | 1.30311 |
| 0.550 | 0.550 | 0.113 | 0.113 | 0.000 | 0.113 | 0.113 | 1300 | 1.30864 | 800 | 1.33328 |
| 0.650 | 0.650 | 0.088 | 0.088 | 0.000 | 0.088 | 0.088 | 1300 | 1.33329 | 800 | 1.33342 |
| 0.750 | 0.750 | 0.063 | 0.063 | 0.000 | 0.063 | 0.063 | 1300 | 1.33330 | 800 | 1.33355 |
| 0.850 | 0.850 | 0.038 | 0.038 | 0.000 | 0.038 | 0.038 | 1300 | 1.33333 | 800 | 1.33377 |
| 0.950 | 0.950 | 0.013 | 0.013 | 0.000 | 0.013 | 0.013 | 1300 | 1.33339 | 800 | 1.33424 |
| 0.050 | 0.050 | 0.238 | 0.000 | 0.238 | 0.238 | 0.238 | 1300 | 1.14317 | 800 | 1.15612 |
| 0.150 | 0.150 | 0.213 | 0.000 | 0.213 | 0.213 | 0.213 | 1300 | 1.16721 | 800 | 1.19467 |
| 0.250 | 0.250 | 0.188 | 0.000 | 0.188 | 0.188 | 0.188 | 1300 | 1.20012 | 800 | 1.23611 |
| 0.350 | 0.350 | 0.163 | 0.000 | 0.163 | 0.163 | 0.163 | 1300 | 1.23836 | 800 | 1.27812 |
| 0.450 | 0.450 | 0.138 | 0.000 | 0.138 | 0.138 | 0.138 | 1300 | 1.27825 | 800 | 1.31975 |
| 0.550 | 0.550 | 0.113 | 0.000 | 0.113 | 0.113 | 0.113 | 1300 | 1.31918 | 800 | 1.33328 |
| 0.650 | 0.650 | 0.088 | 0.000 | 0.088 | 0.088 | 0.088 | 1300 | 1.33329 | 800 | 1.33340 |
| 0.750 | 0.750 | 0.063 | 0.000 | 0.063 | 0.063 | 0.063 | 1300 | 1.33330 | 800 | 1.33355 |
| 0.850 | 0.850 | 0.038 | 0.000 | 0.038 | 0.038 | 0.038 | 1300 | 1.33332 | 800 | 1.33377 |
| 0.950 | 0.950 | 0.013 | 0.000 | 0.013 | 0.013 | 0.013 | 1300 | 1.33338 | 800 | 1.33424 |
| 0.050 | 0.050 | 0.000 | 0.238 | 0.238 | 0.238 | 0.238 | 1300 | 1.01417 | 800 | 1.02104 |
| 0.150 | 0.150 | 0.000 | 0.213 | 0.213 | 0.213 | 0.213 | 1300 | 1.04162 | 800 | 1.06762 |
| 0.250 | 0.250 | 0.000 | 0.188 | 0.188 | 0.188 | 0.188 | 1300 | 1.07376 | 800 | 1.11707 |
| 0.350 | 0.350 | 0.000 | 0.163 | 0.163 | 0.163 | 0.163 | 1300 | 1.10451 | 800 | 1.16698 |
| 0.450 | 0.450 | 0.000 | 0.138 | 0.138 | 0.138 | 0.138 | 1300 | 1.14312 | 800 | 1.21854 |
| 0.550 | 0.550 | 0.000 | 0.113 | 0.113 | 0.113 | 0.113 | 1300 | 1.20935 | 800 | 1.27413 |
| 0.650 | 0.650 | 0.000 | 0.088 | 0.088 | 0.088 | 0.088 | 1300 | 1.27439 | 800 | 1.32886 |
| 0.750 | 0.750 | 0.000 | 0.063 | 0.063 | 0.063 | 0.063 | 1300 | 1.33329 | 800 | 1.33342 |
| 0.850 | 0.850 | 0.000 | 0.038 | 0.038 | 0.038 | 0.038 | 1300 | 1.33330 | 800 | 1.33365 |
| 0.950 | 0.950 | 0.000 | 0.013 | 0.013 | 0.013 | 0.013 | 1300 | 1.33337 | 800 | 1.33414 |
| 0.050 | 0.050 | 0.190 | 0.190 | 0.190 | 0.190 | 0.190 | 1300 | 1.11600 | 800 | 1.12907 |
| 0.150 | 0.150 | 0.170 | 0.170 | 0.170 | 0.170 | 0.170 | 1300 | 1.14022 | 800 | 1.16816 |
| 0.250 | 0.250 | 0.150 | 0.150 | 0.150 | 0.150 | 0.150 | 1300 | 1.17323 | 800 | 1.20973 |
| 0.350 | 0.350 | 0.130 | 0.130 | 0.130 | 0.130 | 0.130 | 1300 | 1.21265 | 800 | 1.25226 |
| 0.450 | 0.450 | 0.110 | 0.110 | 0.110 | 0.110 | 0.110 | 1300 | 1.25320 | 800 | 1.29411 |
| 0.550 | 0.550 | 0.090 | 0.090 | 0.090 | 0.090 | 0.090 | 1300 | 1.29440 | 800 | 1.33328 |
| 0.650 | 0.650 | 0.070 | 0.070 | 0.070 | 0.070 | 0.070 | 1300 | 1.33329 | 800 | 1.33338 |
| 0.750 | 0.750 | 0.050 | 0.050 | 0.050 | 0.050 | 0.050 | 1300 | 1.33329 | 800 | 1.33354 |
| 0.850 | 0.850 | 0.030 | 0.030 | 0.030 | 0.030 | 0.030 | 1300 | 1.33332 | 800 | 1.33376 |
| 0.950 | 0.950 | 0.010 | 0.010 | 0.010 | 0.010 | 0.010 | 1300 | 1.33338 | 800 | 1.33422 |
| 0.050 | 0.050 | 0.000 | 0.000 | 0.000 | 0.000 | 0.950 | 1100 | 1.18024 | 900 | 1.18424 |
| 0.150 | 0.150 | 0.000 | 0.000 | 0.000 | 0.000 | 0.850 | 1100 | 1.33328 | 900 | 1.33328 |
| 0.250 | 0.250 | 0.000 | 0.000 | 0.000 | 0.000 | 0.750 | 1100 | 1.33328 | 900 | 1.33328 |
| 0.350 | 0.350 | 0.000 | 0.000 | 0.000 | 0.000 | 0.650 | 1100 | 1.33328 | 900 | 1.33328 |
| 0.450 | 0.450 | 0.000 | 0.000 | 0.000 | 0.000 | 0.550 | 1100 | 1.33328 | 900 | 1.33328 |
| 0.550 | 0.550 | 0.000 | 0.000 | 0.000 | 0.000 | 0.450 | 1100 | 1.33328 | 900 | 1.33328 |
| 0.650 | 0.650 | 0.000 | 0.000 | 0.000 | 0.000 | 0.350 | 1100 | 1.33328 | 900 | 1.33328 |
| 0.750 | 0.750 | 0.000 | 0.000 | 0.000 | 0.000 | 0.250 | 1100 | 1.33329 | 900 | 1.33329 |
| 0.850 | 0.850 | 0.000 | 0.000 | 0.000 | 0.000 | 0.150 | 1100 | 1.33334 | 900 | 1.33362 |
| 0.950 | 0.950 | 0.000 | 0.000 | 0.000 | 0.000 | 0.050 | 1100 | 1.33388 | 900 | 1.33469 |
| 0.050 | 0.050 | 0.475 | 0.000 | 0.000 | 0.000 | 0.475 | 1100 | 1.33328 | 900 | 1.33328 |
| 0.150 | 0.150 | 0.425 | 0.000 | 0.000 | 0.000 | 0.425 | 1100 | 1.33328 | 900 | 1.33328 |
| 0.250 | 0.250 | 0.375 | 0.000 | 0.000 | 0.000 | 0.375 | 1100 | 1.33328 | 900 | 1.33328 |
| 0.350 | 0.350 | 0.325 | 0.000 | 0.000 | 0.000 | 0.325 | 1100 | 1.33328 | 900 | 1.33328 |
| 0.450 | 0.450 | 0.275 | 0.000 | 0.000 | 0.000 | 0.275 | 1100 | 1.33329 | 900 | 1.33329 |
| 0.550 | 0.550 | 0.225 | 0.000 | 0.000 | 0.000 | 0.225 | 1100 | 1.33329 | 900 | 1.33356 |
| 0.650 | 0.650 | 0.175 | 0.000 | 0.000 | 0.000 | 0.175 | 1100 | 1.33332 | 900 | 1.33383 |
| 0.750 | 0.750 | 0.125 | 0.000 | 0.000 | 0.000 | 0.125 | 1100 | 1.33340 | 900 | 1.33394 |
| 0.850 | 0.850 | 0.075 | 0.000 | 0.000 | 0.000 | 0.075 | 1100 | 1.33366 | 900 | 1.33436 |
| 0.950 | 0.950 | 0.025 | 0.000 | 0.000 | 0.000 | 0.025 | 1100 | 1.33437 | 900 | 1.33536 |
| 0.050 | 0.050 | 0.000 | 0.475 | 0.000 | 0.000 | 0.475 | 1100 | 1.03025 | 900 | 1.03325 |
| 0.150 | 0.150 | 0.000 | 0.425 | 0.000 | 0.000 | 0.425 | 1100 | 1.10503 | 900 | 1.10831 |
| 0.250 | 0.250 | 0.000 | 0.375 | 0.000 | 0.000 | 0.375 | 1100 | 1.17176 | 900 | 1.17476 |
| 0.350 | 0.350 | 0.000 | 0.325 | 0.000 | 0.000 | 0.325 | 1100 | 1.23176 | 900 | 1.23493 |
| 0.450 | 0.450 | 0.000 | 0.275 | 0.000 | 0.000 | 0.275 | 1100 | 1.28674 | 900 | 1.29027 |
| 0.550 | 0.550 | 0.000 | 0.225 | 0.000 | 0.000 | 0.225 | 1100 | 1.33328 | 900 | 1.33329 |
| 0.650 | 0.650 | 0.000 | 0.175 | 0.000 | 0.000 | 0.175 | 1100 | 1.33329 | 900 | 1.33330 |
| 0.750 | 0.750 | 0.000 | 0.125 | 0.000 | 0.000 | 0.125 | 1100 | 1.33330 | 900 | 1.33342 |
| 0.850 | 0.850 | 0.000 | 0.075 | 0.000 | 0.000 | 0.075 | 1100 | 1.33343 | 900 | 1.33381 |
| 0.950 | 0.950 | 0.000 | 0.025 | 0.000 | 0.000 | 0.025 | 1100 | 1.33408 | 900 | 1.33489 |
| 0.050 | 0.050 | 0.000 | 0.000 | 0.475 | 0.000 | 0.475 | 1100 | 1.04947 | 900 | 1.05091 |
| 0.150 | 0.150 | 0.000 | 0.000 | 0.425 | 0.000 | 0.425 | 1100 | 1.10222 | 900 | 1.10545 |
| 0.250 | 0.250 | 0.000 | 0.000 | 0.375 | 0.000 | 0.375 | 1100 | 1.17175 | 900 | 1.17505 |
| 0.350 | 0.350 | 0.000 | 0.000 | 0.325 | 0.000 | 0.325 | 1100 | 1.23576 | 900 | 1.23929 |
| 0.450 | 0.450 | 0.000 | 0.000 | 0.275 | 0.000 | 0.275 | 1100 | 1.29441 | 900 | 1.29837 |
| 0.550 | 0.550 | 0.000 | 0.000 | 0.225 | 0.000 | 0.225 | 1100 | 1.33328 | 900 | 1.33328 |
| 0.650 | 0.650 | 0.000 | 0.000 | 0.175 | 0.000 | 0.175 | 1100 | 1.33329 | 900 | 1.33330 |
| 0.750 | 0.750 | 0.000 | 0.000 | 0.125 | 0.000 | 0.125 | 1100 | 1.33331 | 900 | 1.33348 |
| 0.850 | 0.850 | 0.000 | 0.000 | 0.075 | 0.000 | 0.075 | 1100 | 1.33345 | 900 | 1.33388 |
| 0.950 | 0.950 | 0.000 | 0.000 | 0.025 | 0.000 | 0.025 | 1100 | 1.33409 | 900 | 1.33491 |
| 0.050 | 0.050 | 0.000 | 0.000 | 0.000 | 0.475 | 0.475 | 1100 | 1.02775 | 900 | 1.03107 |
| 0.150 | 0.150 | 0.000 | 0.000 | 0.000 | 0.425 | 0.425 | 1100 | 1.10852 | 900 | 1.11208 |
| 0.250 | 0.250 | 0.000 | 0.000 | 0.000 | 0.375 | 0.375 | 1100 | 1.17253 | 900 | 1.17587 |
| 0.350 | 0.350 | 0.000 | 0.000 | 0.000 | 0.325 | 0.325 | 1100 | 1.22639 | 900 | 1.22988 |
| 0.450 | 0.450 | 0.000 | 0.000 | 0.000 | 0.275 | 0.275 | 1100 | 1.27715 | 900 | 1.28125 |
| 0.550 | 0.550 | 0.000 | 0.000 | 0.000 | 0.225 | 0.225 | 1100 | 1.32522 | 900 | 1.33041 |
| 0.650 | 0.650 | 0.000 | 0.000 | 0.000 | 0.175 | 0.175 | 1100 | 1.33329 | 900 | 1.33330 |
| 0.750 | 0.750 | 0.000 | 0.000 | 0.000 | 0.125 | 0.125 | 1100 | 1.33330 | 900 | 1.33342 |
| 0.850 | 0.850 | 0.000 | 0.000 | 0.000 | 0.075 | 0.075 | 1100 | 1.33342 | 900 | 1.33381 |
| 0.950 | 0.950 | 0.000 | 0.000 | 0.000 | 0.025 | 0.025 | 1100 | 1.33408 | 900 | 1.33489 |
| 0.050 | 0.050 | 0.317 | 0.317 | 0.000 | 0.000 | 0.317 | 1100 | 1.29193 | 900 | 1.29498 |
| 0.150 | 0.150 | 0.283 | 0.283 | 0.000 | 0.000 | 0.283 | 1100 | 1.33223 | 900 | 1.33328 |
| 0.250 | 0.250 | 0.250 | 0.250 | 0.000 | 0.000 | 0.250 | 1100 | 1.33328 | 900 | 1.33328 |
| 0.350 | 0.350 | 0.217 | 0.217 | 0.000 | 0.000 | 0.217 | 1100 | 1.33328 | 900 | 1.33329 |
| 0.450 | 0.450 | 0.183 | 0.183 | 0.000 | 0.000 | 0.183 | 1100 | 1.33329 | 900 | 1.33329 |
| 0.550 | 0.550 | 0.150 | 0.150 | 0.000 | 0.000 | 0.150 | 1100 | 1.33329 | 900 | 1.33333 |
| 0.650 | 0.650 | 0.117 | 0.117 | 0.000 | 0.000 | 0.117 | 1100 | 1.33333 | 900 | 1.33349 |
| 0.750 | 0.750 | 0.083 | 0.083 | 0.000 | 0.000 | 0.083 | 1100 | 1.33342 | 900 | 1.33377 |
| 0.850 | 0.850 | 0.050 | 0.050 | 0.000 | 0.000 | 0.050 | 1100 | 1.33368 | 900 | 1.33426 |
| 0.950 | 0.950 | 0.017 | 0.017 | 0.000 | 0.000 | 0.017 | 1100 | 1.33437 | 900 | 1.33528 |
| 0.050 | 0.050 | 0.317 | 0.000 | 0.317 | 0.000 | 0.317 | 1100 | 1.28074 | 900 | 1.28399 |
| 0.150 | 0.150 | 0.283 | 0.000 | 0.283 | 0.000 | 0.283 | 1100 | 1.31410 | 900 | 1.31788 |
| 0.250 | 0.250 | 0.250 | 0.000 | 0.250 | 0.000 | 0.250 | 1100 | 1.33328 | 900 | 1.33328 |
| 0.350 | 0.350 | 0.217 | 0.000 | 0.217 | 0.000 | 0.217 | 1100 | 1.33328 | 900 | 1.33328 |
| 0.450 | 0.450 | 0.183 | 0.000 | 0.183 | 0.000 | 0.183 | 1100 | 1.33328 | 900 | 1.33329 |
| 0.550 | 0.550 | 0.150 | 0.000 | 0.150 | 0.000 | 0.150 | 1100 | 1.33329 | 900 | 1.33334 |
| 0.650 | 0.650 | 0.117 | 0.000 | 0.117 | 0.000 | 0.117 | 1100 | 1.33332 | 900 | 1.33351 |
| 0.750 | 0.750 | 0.083 | 0.000 | 0.083 | 0.000 | 0.083 | 1100 | 1.33342 | 900 | 1.33380 |
| 0.850 | 0.850 | 0.050 | 0.000 | 0.050 | 0.000 | 0.050 | 1100 | 1.33368 | 900 | 1.33429 |
| 0.950 | 0.950 | 0.017 | 0.000 | 0.017 | 0.000 | 0.017 | 1100 | 1.33437 | 900 | 1.33529 |
| 0.050 | 0.050 | 0.317 | 0.000 | 0.000 | 0.317 | 0.317 | 1100 | 1.23293 | 900 | 1.23612 |
| 0.150 | 0.150 | 0.283 | 0.000 | 0.000 | 0.283 | 0.283 | 1100 | 1.26496 | 900 | 1.26908 |
| 0.250 | 0.250 | 0.250 | 0.000 | 0.000 | 0.250 | 0.250 | 1100 | 1.29916 | 900 | 1.30377 |
| 0.350 | 0.350 | 0.217 | 0.000 | 0.000 | 0.217 | 0.217 | 1100 | 1.33260 | 900 | 1.33328 |
| 0.450 | 0.450 | 0.183 | 0.000 | 0.000 | 0.183 | 0.183 | 1100 | 1.33329 | 900 | 1.33330 |
| 0.550 | 0.550 | 0.150 | 0.000 | 0.000 | 0.150 | 0.150 | 1100 | 1.33329 | 900 | 1.33335 |
| 0.650 | 0.650 | 0.117 | 0.000 | 0.000 | 0.117 | 0.117 | 1100 | 1.33332 | 900 | 1.33350 |
| 0.750 | 0.750 | 0.083 | 0.000 | 0.000 | 0.083 | 0.083 | 1100 | 1.33342 | 900 | 1.33376 |
| 0.850 | 0.850 | 0.050 | 0.000 | 0.000 | 0.050 | 0.050 | 1100 | 1.33367 | 900 | 1.33426 |
| 0.950 | 0.950 | 0.017 | 0.000 | 0.000 | 0.017 | 0.017 | 1100 | 1.33437 | 900 | 1.33528 |
| 0.050 | 0.050 | 0.000 | 0.317 | 0.317 | 0.000 | 0.317 | 1100 | 1.03296 | 900 | 1.03465 |
| 0.150 | 0.150 | 0.000 | 0.283 | 0.283 | 0.000 | 0.283 | 1100 | 1.08521 | 900 | 1.08974 |
| 0.250 | 0.250 | 0.000 | 0.250 | 0.250 | 0.000 | 0.250 | 1100 | 1.15295 | 900 | 1.15728 |
| 0.350 | 0.350 | 0.000 | 0.217 | 0.217 | 0.000 | 0.217 | 1100 | 1.21632 | 900 | 1.22077 |
| 0.450 | 0.450 | 0.000 | 0.183 | 0.183 | 0.000 | 0.183 | 1100 | 1.27443 | 900 | 1.27923 |
| 0.550 | 0.550 | 0.000 | 0.150 | 0.150 | 0.000 | 0.150 | 1100 | 1.32644 | 900 | 1.33178 |
| 0.650 | 0.650 | 0.000 | 0.117 | 0.117 | 0.000 | 0.117 | 1100 | 1.33328 | 900 | 1.33329 |
| 0.750 | 0.750 | 0.000 | 0.083 | 0.083 | 0.000 | 0.083 | 1100 | 1.33331 | 900 | 1.33348 |
| 0.850 | 0.850 | 0.000 | 0.050 | 0.050 | 0.000 | 0.050 | 1100 | 1.33346 | 900 | 1.33395 |
| 0.950 | 0.950 | 0.000 | 0.017 | 0.017 | 0.000 | 0.017 | 1100 | 1.33415 | 900 | 1.33502 |
| 0.050 | 0.050 | 0.000 | 0.317 | 0.000 | 0.317 | 0.317 | 1100 | 1.02425 | 900 | 1.02719 |
| 0.150 | 0.150 | 0.000 | 0.283 | 0.000 | 0.283 | 0.283 | 1100 | 1.08510 | 900 | 1.08865 |
| 0.250 | 0.250 | 0.000 | 0.250 | 0.000 | 0.250 | 0.250 | 1100 | 1.14694 | 900 | 1.15060 |
| 0.350 | 0.350 | 0.000 | 0.217 | 0.000 | 0.217 | 0.217 | 1100 | 1.20454 | 900 | 1.20843 |
| 0.450 | 0.450 | 0.000 | 0.183 | 0.000 | 0.183 | 0.183 | 1100 | 1.25812 | 900 | 1.26230 |
| 0.550 | 0.550 | 0.000 | 0.150 | 0.000 | 0.150 | 0.150 | 1100 | 1.30747 | 900 | 1.31194 |
| 0.650 | 0.650 | 0.000 | 0.117 | 0.000 | 0.117 | 0.117 | 1100 | 1.33329 | 900 | 1.33330 |
| 0.750 | 0.750 | 0.000 | 0.083 | 0.000 | 0.083 | 0.083 | 1100 | 1.33331 | 900 | 1.33348 |
| 0.850 | 0.850 | 0.000 | 0.050 | 0.000 | 0.050 | 0.050 | 1100 | 1.33346 | 900 | 1.33393 |
| 0.950 | 0.950 | 0.000 | 0.017 | 0.000 | 0.017 | 0.017 | 1100 | 1.33415 | 900 | 1.33501 |
| 0.050 | 0.050 | 0.000 | 0.000 | 0.317 | 0.317 | 0.317 | 1100 | 1.03163 | 900 | 1.03374 |
| 0.150 | 0.150 | 0.000 | 0.000 | 0.283 | 0.283 | 0.283 | 1100 | 1.09069 | 900 | 1.09504 |
| 0.250 | 0.250 | 0.000 | 0.000 | 0.250 | 0.250 | 0.250 | 1100 | 1.16059 | 900 | 1.16495 |
| 0.350 | 0.350 | 0.000 | 0.000 | 0.217 | 0.217 | 0.217 | 1100 | 1.22601 | 900 | 1.23057 |
| 0.450 | 0.450 | 0.000 | 0.000 | 0.183 | 0.183 | 0.183 | 1100 | 1.28564 | 900 | 1.29060 |
| 0.550 | 0.550 | 0.000 | 0.000 | 0.150 | 0.150 | 0.150 | 1100 | 1.33328 | 900 | 1.33328 |
| 0.650 | 0.650 | 0.000 | 0.000 | 0.117 | 0.117 | 0.117 | 1100 | 1.33328 | 900 | 1.33329 |
| 0.750 | 0.750 | 0.000 | 0.000 | 0.083 | 0.083 | 0.083 | 1100 | 1.33331 | 900 | 1.33349 |
| 0.850 | 0.850 | 0.000 | 0.000 | 0.050 | 0.050 | 0.050 | 1100 | 1.33346 | 900 | 1.33395 |
| 0.950 | 0.950 | 0.000 | 0.000 | 0.017 | 0.017 | 0.017 | 1100 | 1.33415 | 900 | 1.33502 |
| 0.050 | 0.050 | 0.238 | 0.238 | 0.238 | 0.000 | 0.238 | 1100 | 1.20123 | 900 | 1.20448 |
| 0.150 | 0.150 | 0.213 | 0.213 | 0.213 | 0.000 | 0.213 | 1100 | 1.24931 | 900 | 1.25332 |
| 0.250 | 0.250 | 0.188 | 0.188 | 0.188 | 0.000 | 0.188 | 1100 | 1.29007 | 900 | 1.29456 |
| 0.350 | 0.350 | 0.163 | 0.163 | 0.163 | 0.000 | 0.163 | 1100 | 1.32534 | 900 | 1.33029 |
| 0.450 | 0.450 | 0.138 | 0.138 | 0.138 | 0.000 | 0.138 | 1100 | 1.33328 | 900 | 1.33328 |
| 0.550 | 0.550 | 0.113 | 0.113 | 0.113 | 0.000 | 0.113 | 1100 | 1.33329 | 900 | 1.33330 |
| 0.650 | 0.650 | 0.088 | 0.088 | 0.088 | 0.000 | 0.088 | 1100 | 1.33331 | 900 | 1.33346 |
| 0.750 | 0.750 | 0.063 | 0.063 | 0.063 | 0.000 | 0.063 | 1100 | 1.33340 | 900 | 1.33377 |
| 0.850 | 0.850 | 0.038 | 0.038 | 0.038 | 0.000 | 0.038 | 1100 | 1.33366 | 900 | 1.33428 |
| 0.950 | 0.950 | 0.013 | 0.013 | 0.013 | 0.000 | 0.013 | 1100 | 1.33436 | 900 | 1.33529 |
| 0.050 | 0.050 | 0.238 | 0.238 | 0.000 | 0.238 | 0.238 | 1100 | 1.18343 | 900 | 1.18646 |
| 0.150 | 0.150 | 0.213 | 0.213 | 0.000 | 0.213 | 0.213 | 1100 | 1.22949 | 900 | 1.23325 |
| 0.250 | 0.250 | 0.188 | 0.188 | 0.000 | 0.188 | 0.188 | 1100 | 1.27005 | 900 | 1.27431 |
| 0.350 | 0.350 | 0.163 | 0.163 | 0.000 | 0.163 | 0.163 | 1100 | 1.30593 | 900 | 1.31052 |
| 0.450 | 0.450 | 0.138 | 0.138 | 0.000 | 0.138 | 0.138 | 1100 | 1.33328 | 900 | 1.33329 |
| 0.550 | 0.550 | 0.113 | 0.113 | 0.000 | 0.113 | 0.113 | 1100 | 1.33329 | 900 | 1.33332 |
| 0.650 | 0.650 | 0.088 | 0.088 | 0.000 | 0.088 | 0.088 | 1100 | 1.33331 | 900 | 1.33349 |
| 0.750 | 0.750 | 0.063 | 0.063 | 0.000 | 0.063 | 0.063 | 1100 | 1.33340 | 900 | 1.33378 |
| 0.850 | 0.850 | 0.038 | 0.038 | 0.000 | 0.038 | 0.038 | 1100 | 1.33366 | 900 | 1.33428 |
| 0.950 | 0.950 | 0.013 | 0.013 | 0.000 | 0.013 | 0.013 | 1100 | 1.33436 | 900 | 1.33529 |
| 0.050 | 0.050 | 0.238 | 0.000 | 0.238 | 0.238 | 0.238 | 1100 | 1.19304 | 900 | 1.19645 |
| 0.150 | 0.150 | 0.213 | 0.000 | 0.213 | 0.213 | 0.213 | 1100 | 1.24503 | 900 | 1.24935 |
| 0.250 | 0.250 | 0.188 | 0.000 | 0.188 | 0.188 | 0.188 | 1100 | 1.28988 | 900 | 1.29483 |
| 0.350 | 0.350 | 0.163 | 0.000 | 0.163 | 0.163 | 0.163 | 1100 | 1.32851 | 900 | 1.33328 |
| 0.450 | 0.450 | 0.138 | 0.000 | 0.138 | 0.138 | 0.138 | 1100 | 1.33328 | 900 | 1.33328 |
| 0.550 | 0.550 | 0.113 | 0.000 | 0.113 | 0.113 | 0.113 | 1100 | 1.33329 | 900 | 1.33330 |
| 0.650 | 0.650 | 0.088 | 0.000 | 0.088 | 0.088 | 0.088 | 1100 | 1.33331 | 900 | 1.33346 |
| 0.750 | 0.750 | 0.063 | 0.000 | 0.063 | 0.063 | 0.063 | 1100 | 1.33339 | 900 | 1.33377 |
| 0.850 | 0.850 | 0.038 | 0.000 | 0.038 | 0.038 | 0.038 | 1100 | 1.33365 | 900 | 1.33428 |
| 0.950 | 0.950 | 0.013 | 0.000 | 0.013 | 0.013 | 0.013 | 1100 | 1.33436 | 900 | 1.33529 |
| 0.050 | 0.050 | 0.000 | 0.238 | 0.238 | 0.238 | 0.238 | 1100 | 1.02711 | 900 | 1.02923 |
| 0.150 | 0.150 | 0.000 | 0.213 | 0.213 | 0.213 | 0.213 | 1100 | 1.08077 | 900 | 1.08575 |
| 0.250 | 0.250 | 0.000 | 0.188 | 0.188 | 0.188 | 0.188 | 1100 | 1.14521 | 900 | 1.14997 |
| 0.350 | 0.350 | 0.000 | 0.163 | 0.163 | 0.163 | 0.163 | 1100 | 1.20541 | 900 | 1.21024 |
| 0.450 | 0.450 | 0.000 | 0.138 | 0.138 | 0.138 | 0.138 | 1100 | 1.26066 | 900 | 1.26568 |
| 0.550 | 0.550 | 0.000 | 0.113 | 0.113 | 0.113 | 0.113 | 1100 | 1.31045 | 900 | 1.31553 |
| 0.650 | 0.650 | 0.000 | 0.088 | 0.088 | 0.088 | 0.088 | 1100 | 1.33328 | 900 | 1.33329 |
| 0.750 | 0.750 | 0.000 | 0.063 | 0.063 | 0.063 | 0.063 | 1100 | 1.33331 | 900 | 1.33349 |
| 0.850 | 0.850 | 0.000 | 0.038 | 0.038 | 0.038 | 0.038 | 1100 | 1.33348 | 900 | 1.33399 |
| 0.950 | 0.950 | 0.000 | 0.013 | 0.013 | 0.013 | 0.013 | 1100 | 1.33419 | 900 | 1.33508 |
| 0.050 | 0.050 | 0.190 | 0.190 | 0.190 | 0.190 | 0.190 | 1100 | 1.15223 | 900 | 1.15571 |
| 0.150 | 0.150 | 0.170 | 0.170 | 0.170 | 0.170 | 0.170 | 1100 | 1.20338 | 900 | 1.20767 |
| 0.250 | 0.250 | 0.150 | 0.150 | 0.150 | 0.150 | 0.150 | 1100 | 1.24798 | 900 | 1.25261 |
| 0.350 | 0.350 | 0.130 | 0.130 | 0.130 | 0.130 | 0.130 | 1100 | 1.28755 | 900 | 1.29236 |
| 0.450 | 0.450 | 0.110 | 0.110 | 0.110 | 0.110 | 0.110 | 1100 | 1.32361 | 900 | 1.32836 |
| 0.550 | 0.550 | 0.090 | 0.090 | 0.090 | 0.090 | 0.090 | 1100 | 1.33328 | 900 | 1.33329 |
| 0.650 | 0.650 | 0.070 | 0.070 | 0.070 | 0.070 | 0.070 | 1100 | 1.33330 | 900 | 1.33341 |
| 0.750 | 0.750 | 0.050 | 0.050 | 0.050 | 0.050 | 0.050 | 1100 | 1.33338 | 900 | 1.33374 |
| 0.850 | 0.850 | 0.030 | 0.030 | 0.030 | 0.030 | 0.030 | 1100 | 1.33364 | 900 | 1.33427 |
| 0.950 | 0.950 | 0.010 | 0.010 | 0.010 | 0.010 | 0.010 | 1100 | 1.33435 | 900 | 1.33529 |
| 0.050 | 0.050 | 0.000 | 0.000 | 0.000 | 0.000 | 0.950 | 1000 | 1.33328 | 800 | 1.33328 |
| 0.150 | 0.150 | 0.000 | 0.000 | 0.000 | 0.000 | 0.850 | 1000 | 1.33328 | 800 | 1.33328 |
| 0.250 | 0.250 | 0.000 | 0.000 | 0.000 | 0.000 | 0.750 | 1000 | 1.33328 | 800 | 1.33328 |
| 0.350 | 0.350 | 0.000 | 0.000 | 0.000 | 0.000 | 0.650 | 1000 | 1.33328 | 800 | 1.33328 |
| 0.450 | 0.450 | 0.000 | 0.000 | 0.000 | 0.000 | 0.550 | 1000 | 1.33328 | 800 | 1.33328 |
| 0.550 | 0.550 | 0.000 | 0.000 | 0.000 | 0.000 | 0.450 | 1000 | 1.33328 | 800 | 1.33328 |
| 0.650 | 0.650 | 0.000 | 0.000 | 0.000 | 0.000 | 0.350 | 1000 | 1.33328 | 800 | 1.33328 |
| 0.750 | 0.750 | 0.000 | 0.000 | 0.000 | 0.000 | 0.250 | 1000 | 1.33329 | 800 | 1.33335 |
| 0.850 | 0.850 | 0.000 | 0.000 | 0.000 | 0.000 | 0.150 | 1000 | 1.33342 | 800 | 1.33372 |
| 0.950 | 0.950 | 0.000 | 0.000 | 0.000 | 0.000 | 0.050 | 1000 | 1.33542 | 800 | 1.33619 |
| 0.050 | 0.050 | 0.475 | 0.000 | 0.000 | 0.000 | 0.475 | 1000 | 1.33328 | 800 | 1.33328 |
| 0.150 | 0.150 | 0.425 | 0.000 | 0.000 | 0.000 | 0.425 | 1000 | 1.33328 | 800 | 1.33328 |
| 0.250 | 0.250 | 0.375 | 0.000 | 0.000 | 0.000 | 0.375 | 1000 | 1.33328 | 800 | 1.33328 |
| 0.350 | 0.350 | 0.325 | 0.000 | 0.000 | 0.000 | 0.325 | 1000 | 1.33328 | 800 | 1.33331 |
| 0.450 | 0.450 | 0.275 | 0.000 | 0.000 | 0.000 | 0.275 | 1000 | 1.33329 | 800 | 1.33341 |
| 0.550 | 0.550 | 0.225 | 0.000 | 0.000 | 0.000 | 0.225 | 1000 | 1.33330 | 800 | 1.33361 |
| 0.650 | 0.650 | 0.175 | 0.000 | 0.000 | 0.000 | 0.175 | 1000 | 1.33334 | 800 | 1.33396 |
| 0.750 | 0.750 | 0.125 | 0.000 | 0.000 | 0.000 | 0.125 | 1000 | 1.33356 | 800 | 1.33427 |
| 0.850 | 0.850 | 0.075 | 0.000 | 0.000 | 0.000 | 0.075 | 1000 | 1.33446 | 800 | 1.33514 |
| 0.950 | 0.950 | 0.025 | 0.000 | 0.000 | 0.000 | 0.025 | 1000 | 1.33745 | 800 | 1.33840 |
| 0.050 | 0.050 | 0.000 | 0.475 | 0.000 | 0.000 | 0.475 | 1000 | 1.11453 | 800 | 1.11508 |
| 0.150 | 0.150 | 0.000 | 0.425 | 0.000 | 0.000 | 0.425 | 1000 | 1.18701 | 800 | 1.18752 |
| 0.250 | 0.250 | 0.000 | 0.375 | 0.000 | 0.000 | 0.375 | 1000 | 1.26760 | 800 | 1.26819 |
| 0.350 | 0.350 | 0.000 | 0.325 | 0.000 | 0.000 | 0.325 | 1000 | 1.31772 | 800 | 1.31846 |
| 0.450 | 0.450 | 0.000 | 0.275 | 0.000 | 0.000 | 0.275 | 1000 | 1.33328 | 800 | 1.33328 |
| 0.550 | 0.550 | 0.000 | 0.225 | 0.000 | 0.000 | 0.225 | 1000 | 1.33328 | 800 | 1.33329 |
| 0.650 | 0.650 | 0.000 | 0.175 | 0.000 | 0.000 | 0.175 | 1000 | 1.33329 | 800 | 1.33330 |
| 0.750 | 0.750 | 0.000 | 0.125 | 0.000 | 0.000 | 0.125 | 1000 | 1.33334 | 800 | 1.33341 |
| 0.850 | 0.850 | 0.000 | 0.075 | 0.000 | 0.000 | 0.075 | 1000 | 1.33378 | 800 | 1.33405 |
| 0.950 | 0.950 | 0.000 | 0.025 | 0.000 | 0.000 | 0.025 | 1000 | 1.33639 | 800 | 1.33715 |
| 0.050 | 0.050 | 0.000 | 0.000 | 0.475 | 0.000 | 0.475 | 1000 | 1.09793 | 800 | 1.09843 |
| 0.150 | 0.150 | 0.000 | 0.000 | 0.425 | 0.000 | 0.425 | 1000 | 1.19727 | 800 | 1.19782 |
| 0.250 | 0.250 | 0.000 | 0.000 | 0.375 | 0.000 | 0.375 | 1000 | 1.26064 | 800 | 1.26123 |
| 0.350 | 0.350 | 0.000 | 0.000 | 0.325 | 0.000 | 0.325 | 1000 | 1.31218 | 800 | 1.31286 |
| 0.450 | 0.450 | 0.000 | 0.000 | 0.275 | 0.000 | 0.275 | 1000 | 1.33328 | 800 | 1.33328 |
| 0.550 | 0.550 | 0.000 | 0.000 | 0.225 | 0.000 | 0.225 | 1000 | 1.33328 | 800 | 1.33329 |
| 0.650 | 0.650 | 0.000 | 0.000 | 0.175 | 0.000 | 0.175 | 1000 | 1.33329 | 800 | 1.33331 |
| 0.750 | 0.750 | 0.000 | 0.000 | 0.125 | 0.000 | 0.125 | 1000 | 1.33337 | 800 | 1.33347 |
| 0.850 | 0.850 | 0.000 | 0.000 | 0.075 | 0.000 | 0.075 | 1000 | 1.33388 | 800 | 1.33415 |
| 0.950 | 0.950 | 0.000 | 0.000 | 0.025 | 0.000 | 0.025 | 1000 | 1.33646 | 800 | 1.33721 |
| 0.050 | 0.050 | 0.000 | 0.000 | 0.000 | 0.475 | 0.475 | 1000 | 1.13284 | 800 | 1.13340 |
| 0.150 | 0.150 | 0.000 | 0.000 | 0.000 | 0.425 | 0.425 | 1000 | 1.18560 | 800 | 1.18617 |
| 0.250 | 0.250 | 0.000 | 0.000 | 0.000 | 0.375 | 0.375 | 1000 | 1.22951 | 800 | 1.23026 |
| 0.350 | 0.350 | 0.000 | 0.000 | 0.000 | 0.325 | 0.325 | 1000 | 1.27838 | 800 | 1.27926 |
| 0.450 | 0.450 | 0.000 | 0.000 | 0.000 | 0.275 | 0.275 | 1000 | 1.32704 | 800 | 1.32811 |
| 0.550 | 0.550 | 0.000 | 0.000 | 0.000 | 0.225 | 0.225 | 1000 | 1.33328 | 800 | 1.33329 |
| 0.650 | 0.650 | 0.000 | 0.000 | 0.000 | 0.175 | 0.175 | 1000 | 1.33329 | 800 | 1.33330 |
| 0.750 | 0.750 | 0.000 | 0.000 | 0.000 | 0.125 | 0.125 | 1000 | 1.33334 | 800 | 1.33341 |
| 0.850 | 0.850 | 0.000 | 0.000 | 0.000 | 0.075 | 0.075 | 1000 | 1.33378 | 800 | 1.33404 |
| 0.950 | 0.950 | 0.000 | 0.000 | 0.000 | 0.025 | 0.025 | 1000 | 1.33640 | 800 | 1.33715 |
| 0.050 | 0.050 | 0.317 | 0.317 | 0.000 | 0.000 | 0.317 | 1000 | 1.33328 | 800 | 1.33328 |
| 0.150 | 0.150 | 0.283 | 0.283 | 0.000 | 0.000 | 0.283 | 1000 | 1.33328 | 800 | 1.33328 |
| 0.250 | 0.250 | 0.250 | 0.250 | 0.000 | 0.000 | 0.250 | 1000 | 1.33328 | 800 | 1.33328 |
| 0.350 | 0.350 | 0.217 | 0.217 | 0.000 | 0.000 | 0.217 | 1000 | 1.33328 | 800 | 1.33329 |
| 0.450 | 0.450 | 0.183 | 0.183 | 0.000 | 0.000 | 0.183 | 1000 | 1.33329 | 800 | 1.33329 |
| 0.550 | 0.550 | 0.150 | 0.150 | 0.000 | 0.000 | 0.150 | 1000 | 1.33331 | 800 | 1.33333 |
| 0.650 | 0.650 | 0.117 | 0.117 | 0.000 | 0.000 | 0.117 | 1000 | 1.33341 | 800 | 1.33350 |
| 0.750 | 0.750 | 0.083 | 0.083 | 0.000 | 0.000 | 0.083 | 1000 | 1.33373 | 800 | 1.33397 |
| 0.850 | 0.850 | 0.050 | 0.050 | 0.000 | 0.000 | 0.050 | 1000 | 1.33471 | 800 | 1.33521 |
| 0.950 | 0.950 | 0.017 | 0.017 | 0.000 | 0.000 | 0.017 | 1000 | 1.33756 | 800 | 1.33844 |
| 0.050 | 0.050 | 0.317 | 0.000 | 0.317 | 0.000 | 0.317 | 1000 | 1.33328 | 800 | 1.33328 |
| 0.150 | 0.150 | 0.283 | 0.000 | 0.283 | 0.000 | 0.283 | 1000 | 1.33328 | 800 | 1.33328 |
| 0.250 | 0.250 | 0.250 | 0.000 | 0.250 | 0.000 | 0.250 | 1000 | 1.33328 | 800 | 1.33328 |
| 0.350 | 0.350 | 0.217 | 0.000 | 0.217 | 0.000 | 0.217 | 1000 | 1.33328 | 800 | 1.33328 |
| 0.450 | 0.450 | 0.183 | 0.000 | 0.183 | 0.000 | 0.183 | 1000 | 1.33329 | 800 | 1.33329 |
| 0.550 | 0.550 | 0.150 | 0.000 | 0.150 | 0.000 | 0.150 | 1000 | 1.33331 | 800 | 1.33335 |
| 0.650 | 0.650 | 0.117 | 0.000 | 0.117 | 0.000 | 0.117 | 1000 | 1.33341 | 800 | 1.33350 |
| 0.750 | 0.750 | 0.083 | 0.000 | 0.083 | 0.000 | 0.083 | 1000 | 1.33375 | 800 | 1.33398 |
| 0.850 | 0.850 | 0.050 | 0.000 | 0.050 | 0.000 | 0.050 | 1000 | 1.33476 | 800 | 1.33524 |
| 0.950 | 0.950 | 0.017 | 0.000 | 0.017 | 0.000 | 0.017 | 1000 | 1.33758 | 800 | 1.33846 |
| 0.050 | 0.050 | 0.317 | 0.000 | 0.000 | 0.317 | 0.317 | 1000 | 1.26046 | 800 | 1.26108 |
| 0.150 | 0.150 | 0.283 | 0.000 | 0.000 | 0.283 | 0.283 | 1000 | 1.29729 | 800 | 1.29805 |
| 0.250 | 0.250 | 0.250 | 0.000 | 0.000 | 0.250 | 0.250 | 1000 | 1.33328 | 800 | 1.33328 |
| 0.350 | 0.350 | 0.217 | 0.000 | 0.000 | 0.217 | 0.217 | 1000 | 1.33328 | 800 | 1.33329 |
| 0.450 | 0.450 | 0.183 | 0.000 | 0.000 | 0.183 | 0.183 | 1000 | 1.33329 | 800 | 1.33331 |
| 0.550 | 0.550 | 0.150 | 0.000 | 0.000 | 0.150 | 0.150 | 1000 | 1.33331 | 800 | 1.33336 |
| 0.650 | 0.650 | 0.117 | 0.000 | 0.000 | 0.117 | 0.117 | 1000 | 1.33340 | 800 | 1.33351 |
| 0.750 | 0.750 | 0.083 | 0.000 | 0.000 | 0.083 | 0.083 | 1000 | 1.33371 | 800 | 1.33393 |
| 0.850 | 0.850 | 0.050 | 0.000 | 0.000 | 0.050 | 0.050 | 1000 | 1.33469 | 800 | 1.33519 |
| 0.950 | 0.950 | 0.017 | 0.000 | 0.000 | 0.017 | 0.017 | 1000 | 1.33756 | 800 | 1.33844 |
| 0.050 | 0.050 | 0.000 | 0.317 | 0.317 | 0.000 | 0.317 | 1000 | 1.05759 | 800 | 1.05822 |
| 0.150 | 0.150 | 0.000 | 0.283 | 0.283 | 0.000 | 0.283 | 1000 | 1.15824 | 800 | 1.15890 |
| 0.250 | 0.250 | 0.000 | 0.250 | 0.250 | 0.000 | 0.250 | 1000 | 1.22663 | 800 | 1.22734 |
| 0.350 | 0.350 | 0.000 | 0.217 | 0.217 | 0.000 | 0.217 | 1000 | 1.27993 | 800 | 1.28072 |
| 0.450 | 0.450 | 0.000 | 0.183 | 0.183 | 0.000 | 0.183 | 1000 | 1.32428 | 800 | 1.32514 |
| 0.550 | 0.550 | 0.000 | 0.150 | 0.150 | 0.000 | 0.150 | 1000 | 1.33328 | 800 | 1.33328 |
| 0.650 | 0.650 | 0.000 | 0.117 | 0.117 | 0.000 | 0.117 | 1000 | 1.33329 | 800 | 1.33330 |
| 0.750 | 0.750 | 0.000 | 0.083 | 0.083 | 0.000 | 0.083 | 1000 | 1.33337 | 800 | 1.33346 |
| 0.850 | 0.850 | 0.000 | 0.050 | 0.050 | 0.000 | 0.050 | 1000 | 1.33399 | 800 | 1.33428 |
| 0.950 | 0.950 | 0.000 | 0.017 | 0.017 | 0.000 | 0.017 | 1000 | 1.33677 | 800 | 1.33753 |
| 0.050 | 0.050 | 0.000 | 0.317 | 0.000 | 0.317 | 0.317 | 1000 | 1.06751 | 800 | 1.06812 |
| 0.150 | 0.150 | 0.000 | 0.283 | 0.000 | 0.283 | 0.283 | 1000 | 1.13630 | 800 | 1.13693 |
| 0.250 | 0.250 | 0.000 | 0.250 | 0.000 | 0.250 | 0.250 | 1000 | 1.20078 | 800 | 1.20144 |
| 0.350 | 0.350 | 0.000 | 0.217 | 0.000 | 0.217 | 0.217 | 1000 | 1.25595 | 800 | 1.25669 |
| 0.450 | 0.450 | 0.000 | 0.183 | 0.000 | 0.183 | 0.183 | 1000 | 1.30295 | 800 | 1.30376 |
| 0.550 | 0.550 | 0.000 | 0.150 | 0.000 | 0.150 | 0.150 | 1000 | 1.33328 | 800 | 1.33329 |
| 0.650 | 0.650 | 0.000 | 0.117 | 0.000 | 0.117 | 0.117 | 1000 | 1.33329 | 800 | 1.33331 |
| 0.750 | 0.750 | 0.000 | 0.083 | 0.000 | 0.083 | 0.083 | 1000 | 1.33337 | 800 | 1.33347 |
| 0.850 | 0.850 | 0.000 | 0.050 | 0.000 | 0.050 | 0.050 | 1000 | 1.33396 | 800 | 1.33424 |
| 0.950 | 0.950 | 0.000 | 0.017 | 0.000 | 0.017 | 0.017 | 1000 | 1.33675 | 800 | 1.33751 |
| 0.050 | 0.050 | 0.000 | 0.000 | 0.317 | 0.317 | 0.317 | 1000 | 1.06520 | 800 | 1.06582 |
| 0.150 | 0.150 | 0.000 | 0.000 | 0.283 | 0.283 | 0.283 | 1000 | 1.16741 | 800 | 1.16809 |
| 0.250 | 0.250 | 0.000 | 0.000 | 0.250 | 0.250 | 0.250 | 1000 | 1.23680 | 800 | 1.23754 |
| 0.350 | 0.350 | 0.000 | 0.000 | 0.217 | 0.217 | 0.217 | 1000 | 1.29016 | 800 | 1.29096 |
| 0.450 | 0.450 | 0.000 | 0.000 | 0.183 | 0.183 | 0.183 | 1000 | 1.33328 | 800 | 1.33328 |
| 0.550 | 0.550 | 0.000 | 0.000 | 0.150 | 0.150 | 0.150 | 1000 | 1.33328 | 800 | 1.33328 |
| 0.650 | 0.650 | 0.000 | 0.000 | 0.117 | 0.117 | 0.117 | 1000 | 1.33329 | 800 | 1.33330 |
| 0.750 | 0.750 | 0.000 | 0.000 | 0.083 | 0.083 | 0.083 | 1000 | 1.33337 | 800 | 1.33347 |
| 0.850 | 0.850 | 0.000 | 0.000 | 0.050 | 0.050 | 0.050 | 1000 | 1.33400 | 800 | 1.33429 |
| 0.950 | 0.950 | 0.000 | 0.000 | 0.017 | 0.017 | 0.017 | 1000 | 1.33678 | 800 | 1.33754 |
| 0.050 | 0.050 | 0.238 | 0.238 | 0.238 | 0.000 | 0.238 | 1000 | 1.25308 | 800 | 1.25362 |
| 0.150 | 0.150 | 0.213 | 0.213 | 0.213 | 0.000 | 0.213 | 1000 | 1.29421 | 800 | 1.29485 |
| 0.250 | 0.250 | 0.188 | 0.188 | 0.188 | 0.000 | 0.188 | 1000 | 1.32739 | 800 | 1.32807 |
| 0.350 | 0.350 | 0.163 | 0.163 | 0.163 | 0.000 | 0.163 | 1000 | 1.33328 | 800 | 1.33328 |
| 0.450 | 0.450 | 0.138 | 0.138 | 0.138 | 0.000 | 0.138 | 1000 | 1.33328 | 800 | 1.33328 |
| 0.550 | 0.550 | 0.113 | 0.113 | 0.113 | 0.000 | 0.113 | 1000 | 1.33329 | 800 | 1.33331 |
| 0.650 | 0.650 | 0.088 | 0.088 | 0.088 | 0.000 | 0.088 | 1000 | 1.33336 | 800 | 1.33345 |
| 0.750 | 0.750 | 0.063 | 0.063 | 0.063 | 0.000 | 0.063 | 1000 | 1.33369 | 800 | 1.33389 |
| 0.850 | 0.850 | 0.038 | 0.038 | 0.038 | 0.000 | 0.038 | 1000 | 1.33472 | 800 | 1.33517 |
| 0.950 | 0.950 | 0.013 | 0.013 | 0.013 | 0.000 | 0.013 | 1000 | 1.33759 | 800 | 1.33843 |
| 0.050 | 0.050 | 0.238 | 0.238 | 0.000 | 0.238 | 0.238 | 1000 | 1.22054 | 800 | 1.22106 |
| 0.150 | 0.150 | 0.213 | 0.213 | 0.000 | 0.213 | 0.213 | 1000 | 1.26834 | 800 | 1.26906 |
| 0.250 | 0.250 | 0.188 | 0.188 | 0.000 | 0.188 | 0.188 | 1000 | 1.30696 | 800 | 1.30780 |
| 0.350 | 0.350 | 0.163 | 0.163 | 0.000 | 0.163 | 0.163 | 1000 | 1.33328 | 800 | 1.33328 |
| 0.450 | 0.450 | 0.138 | 0.138 | 0.000 | 0.138 | 0.138 | 1000 | 1.33328 | 800 | 1.33329 |
| 0.550 | 0.550 | 0.113 | 0.113 | 0.000 | 0.113 | 0.113 | 1000 | 1.33330 | 800 | 1.33333 |
| 0.650 | 0.650 | 0.088 | 0.088 | 0.000 | 0.088 | 0.088 | 1000 | 1.33338 | 800 | 1.33348 |
| 0.750 | 0.750 | 0.063 | 0.063 | 0.000 | 0.063 | 0.063 | 1000 | 1.33372 | 800 | 1.33389 |
| 0.850 | 0.850 | 0.038 | 0.038 | 0.000 | 0.038 | 0.038 | 1000 | 1.33473 | 800 | 1.33516 |
| 0.950 | 0.950 | 0.013 | 0.013 | 0.000 | 0.013 | 0.013 | 1000 | 1.33758 | 800 | 1.33843 |
| 0.050 | 0.050 | 0.238 | 0.000 | 0.238 | 0.238 | 0.238 | 1000 | 1.24606 | 800 | 1.24667 |
| 0.150 | 0.150 | 0.213 | 0.000 | 0.213 | 0.213 | 0.213 | 1000 | 1.29497 | 800 | 1.29573 |
| 0.250 | 0.250 | 0.188 | 0.000 | 0.188 | 0.188 | 0.188 | 1000 | 1.33149 | 800 | 1.33230 |
| 0.350 | 0.350 | 0.163 | 0.000 | 0.163 | 0.163 | 0.163 | 1000 | 1.33328 | 800 | 1.33328 |
| 0.450 | 0.450 | 0.138 | 0.000 | 0.138 | 0.138 | 0.138 | 1000 | 1.33328 | 800 | 1.33328 |
| 0.550 | 0.550 | 0.113 | 0.000 | 0.113 | 0.113 | 0.113 | 1000 | 1.33329 | 800 | 1.33331 |
| 0.650 | 0.650 | 0.088 | 0.000 | 0.088 | 0.088 | 0.088 | 1000 | 1.33336 | 800 | 1.33345 |
| 0.750 | 0.750 | 0.063 | 0.000 | 0.063 | 0.063 | 0.063 | 1000 | 1.33369 | 800 | 1.33389 |
| 0.850 | 0.850 | 0.038 | 0.000 | 0.038 | 0.038 | 0.038 | 1000 | 1.33472 | 800 | 1.33517 |
| 0.950 | 0.950 | 0.013 | 0.000 | 0.013 | 0.013 | 0.013 | 1000 | 1.33759 | 800 | 1.33844 |
| 0.050 | 0.050 | 0.000 | 0.238 | 0.238 | 0.238 | 0.238 | 1000 | 1.04591 | 800 | 1.04664 |
| 0.150 | 0.150 | 0.000 | 0.213 | 0.213 | 0.213 | 0.213 | 1000 | 1.13476 | 800 | 1.13549 |
| 0.250 | 0.250 | 0.000 | 0.188 | 0.188 | 0.188 | 0.188 | 1000 | 1.19980 | 800 | 1.20059 |
| 0.350 | 0.350 | 0.000 | 0.163 | 0.163 | 0.163 | 0.163 | 1000 | 1.25254 | 800 | 1.25338 |
| 0.450 | 0.450 | 0.000 | 0.138 | 0.138 | 0.138 | 0.138 | 1000 | 1.29767 | 800 | 1.29854 |
| 0.550 | 0.550 | 0.000 | 0.113 | 0.113 | 0.113 | 0.113 | 1000 | 1.33328 | 800 | 1.33328 |
| 0.650 | 0.650 | 0.000 | 0.088 | 0.088 | 0.088 | 0.088 | 1000 | 1.33329 | 800 | 1.33329 |
| 0.750 | 0.750 | 0.000 | 0.063 | 0.063 | 0.063 | 0.063 | 1000 | 1.33337 | 800 | 1.33348 |
| 0.850 | 0.850 | 0.000 | 0.038 | 0.038 | 0.038 | 0.038 | 1000 | 1.33406 | 800 | 1.33436 |
| 0.950 | 0.950 | 0.000 | 0.013 | 0.013 | 0.013 | 0.013 | 1000 | 1.33695 | 800 | 1.33771 |
| 0.050 | 0.050 | 0.190 | 0.190 | 0.190 | 0.190 | 0.190 | 1000 | 1.19425 | 800 | 1.19486 |
| 0.150 | 0.150 | 0.170 | 0.170 | 0.170 | 0.170 | 0.170 | 1000 | 1.24341 | 800 | 1.24414 |
| 0.250 | 0.250 | 0.150 | 0.150 | 0.150 | 0.150 | 0.150 | 1000 | 1.28216 | 800 | 1.28294 |
| 0.350 | 0.350 | 0.130 | 0.130 | 0.130 | 0.130 | 0.130 | 1000 | 1.31569 | 800 | 1.31646 |
| 0.450 | 0.450 | 0.110 | 0.110 | 0.110 | 0.110 | 0.110 | 1000 | 1.33328 | 800 | 1.33328 |
| 0.550 | 0.550 | 0.090 | 0.090 | 0.090 | 0.090 | 0.090 | 1000 | 1.33329 | 800 | 1.33329 |
| 0.650 | 0.650 | 0.070 | 0.070 | 0.070 | 0.070 | 0.070 | 1000 | 1.33333 | 800 | 1.33341 |
| 0.750 | 0.750 | 0.050 | 0.050 | 0.050 | 0.050 | 0.050 | 1000 | 1.33364 | 800 | 1.33381 |
| 0.850 | 0.850 | 0.030 | 0.030 | 0.030 | 0.030 | 0.030 | 1000 | 1.33468 | 800 | 1.33510 |
| 0.950 | 0.950 | 0.010 | 0.010 | 0.010 | 0.010 | 0.010 | 1000 | 1.33758 | 800 | 1.33841 |

**Table. S5** CALPHAD simulation results for M_1-x_Fe_x_O_y_ system, excluding Mn. P(O_2_) and input H_2_O were set at 3∙10^-6^ atm and 35.4 mol, respectively.

| Composition M(1-x)Fe(x)O(z) | | | | | | TR | | WS | |
| --- | --- | --- | --- | --- | --- | --- | --- | --- | --- |
| x | Fe | Al | Mg | Co | Ni | T(TR) (℃) | O stoich. | T(WS) (℃) | O stoich. |
| 0.050 | 0.050 | 0.950 | 0.000 | 0.000 | 0.000 | 1300 | 1.48884 | 1300 | 1.49869 |
| 0.150 | 0.150 | 0.850 | 0.000 | 0.000 | 0.000 | 1300 | 1.44815 | 1300 | 1.46546 |
| 0.250 | 0.250 | 0.750 | 0.000 | 0.000 | 0.000 | 1300 | 1.40746 | 1300 | 1.43005 |
| 0.350 | 0.350 | 0.650 | 0.000 | 0.000 | 0.000 | 1300 | 1.36677 | 1300 | 1.39348 |
| 0.450 | 0.450 | 0.550 | 0.000 | 0.000 | 0.000 | 1300 | 1.33570 | 1300 | 1.36096 |
| 0.550 | 0.550 | 0.450 | 0.000 | 0.000 | 0.000 | 1300 | 1.33412 | 1300 | 1.34617 |
| 0.650 | 0.650 | 0.350 | 0.000 | 0.000 | 0.000 | 1300 | 1.33378 | 1300 | 1.33808 |
| 0.750 | 0.750 | 0.250 | 0.000 | 0.000 | 0.000 | 1300 | 1.33364 | 1300 | 1.33672 |
| 0.850 | 0.850 | 0.150 | 0.000 | 0.000 | 0.000 | 1300 | 1.33357 | 1300 | 1.33588 |
| 0.950 | 0.950 | 0.050 | 0.000 | 0.000 | 0.000 | 1300 | 1.33351 | 1300 | 1.33524 |
| 0.050 | 0.050 | 0.000 | 0.950 | 0.000 | 0.000 | 1300 | 1.00743 | 1300 | 1.01316 |
| 0.150 | 0.150 | 0.000 | 0.850 | 0.000 | 0.000 | 1300 | 1.02872 | 1300 | 1.04485 |
| 0.250 | 0.250 | 0.000 | 0.750 | 0.000 | 0.000 | 1300 | 1.05920 | 1300 | 1.08175 |
| 0.350 | 0.350 | 0.000 | 0.650 | 0.000 | 0.000 | 1300 | 1.09416 | 1300 | 1.12486 |
| 0.450 | 0.450 | 0.000 | 0.550 | 0.000 | 0.000 | 1300 | 1.12893 | 1300 | 1.17048 |
| 0.550 | 0.550 | 0.000 | 0.450 | 0.000 | 0.000 | 1300 | 1.19093 | 1300 | 1.22841 |
| 0.650 | 0.650 | 0.000 | 0.350 | 0.000 | 0.000 | 1300 | 1.25293 | 1300 | 1.28577 |
| 0.750 | 0.750 | 0.000 | 0.250 | 0.000 | 0.000 | 1300 | 1.31493 | 1300 | 1.33302 |
| 0.850 | 0.850 | 1.000 | 0.150 | 0.000 | 0.000 | 1300 | 1.33336 | 1300 | 1.33334 |
| 0.950 | 0.950 | 0.000 | 0.050 | 0.000 | 0.000 | 1300 | 1.33342 | 1300 | 1.33419 |
| 0.050 | 0.050 | 0.000 | 0.000 | 0.950 | 0.000 | 1300 | 1.01369 | 1300 | 1.01836 |
| 0.150 | 0.150 | 0.000 | 0.000 | 0.850 | 0.000 | 1300 | 1.03957 | 1300 | 1.05046 |
| 0.250 | 0.250 | 0.000 | 0.000 | 0.750 | 0.000 | 1300 | 1.06413 | 1300 | 1.07890 |
| 0.350 | 0.350 | 0.000 | 0.000 | 0.650 | 0.000 | 1300 | 1.08781 | 1300 | 1.10919 |
| 0.450 | 0.450 | 0.000 | 0.000 | 0.550 | 0.000 | 1300 | 1.11087 | 1300 | 1.15072 |
| 0.550 | 0.550 | 0.000 | 0.000 | 0.450 | 0.000 | 1300 | 1.17084 | 1300 | 1.21258 |
| 0.650 | 0.650 | 0.000 | 0.000 | 0.350 | 0.000 | 1300 | 1.24407 | 1300 | 1.28007 |
| 0.750 | 0.750 | 0.000 | 0.000 | 0.250 | 0.000 | 1300 | 1.31731 | 1300 | 1.33306 |
| 0.850 | 0.850 | 0.000 | 0.000 | 0.150 | 0.000 | 1300 | 1.33335 | 1300 | 1.33329 |
| 0.950 | 0.950 | 0.000 | 0.000 | 0.050 | 0.000 | 1300 | 1.33342 | 1300 | 1.33414 |
| 0.050 | 0.050 | 0.000 | 0.000 | 0.000 | 0.950 | 1300 | 1.00610 | 1300 | 1.01043 |
| 0.150 | 0.150 | 0.000 | 0.000 | 0.000 | 0.850 | 1300 | 1.02458 | 1300 | 1.03656 |
| 0.250 | 0.250 | 0.000 | 0.000 | 0.000 | 0.750 | 1300 | 1.05012 | 1300 | 1.07092 |
| 0.350 | 0.350 | 0.000 | 0.000 | 0.000 | 0.650 | 1300 | 1.07913 | 1300 | 1.11435 |
| 0.450 | 0.450 | 0.000 | 0.000 | 0.000 | 0.550 | 1300 | 1.13526 | 1300 | 1.16998 |
| 0.550 | 0.550 | 0.000 | 0.000 | 0.000 | 0.450 | 1300 | 1.19622 | 1300 | 1.22730 |
| 0.650 | 0.650 | 0.000 | 0.000 | 0.000 | 0.350 | 1300 | 1.25718 | 1300 | 1.28422 |
| 0.750 | 0.750 | 0.000 | 0.000 | 0.000 | 0.250 | 1300 | 1.31814 | 1300 | 1.33254 |
| 0.850 | 0.850 | 0.000 | 0.000 | 0.000 | 0.150 | 1300 | 1.33335 | 1300 | 1.33316 |
| 0.950 | 0.950 | 0.000 | 0.000 | 0.000 | 0.050 | 1300 | 1.33342 | 1300 | 1.33412 |
| 0.050 | 0.050 | 0.475 | 0.475 | 0.000 | 0.000 | 1300 | 1.25677 | 1300 | 1.26000 |
| 0.150 | 0.150 | 0.425 | 0.425 | 0.000 | 0.000 | 1300 | 1.27496 | 1300 | 1.28247 |
| 0.250 | 0.250 | 0.375 | 0.375 | 0.000 | 0.000 | 1300 | 1.29812 | 1300 | 1.30687 |
| 0.350 | 0.350 | 0.325 | 0.325 | 0.000 | 0.000 | 1300 | 1.32274 | 1300 | 1.33162 |
| 0.450 | 0.450 | 0.275 | 0.275 | 0.000 | 0.000 | 1300 | 1.33334 | 1300 | 1.33316 |
| 0.550 | 0.550 | 0.225 | 0.225 | 0.000 | 0.000 | 1300 | 1.33336 | 1300 | 1.33338 |
| 0.650 | 0.650 | 0.175 | 0.175 | 0.000 | 0.000 | 1300 | 1.33338 | 1300 | 1.33368 |
| 0.750 | 0.750 | 0.125 | 0.125 | 0.000 | 0.000 | 1300 | 1.33340 | 1300 | 1.33398 |
| 0.850 | 0.850 | 0.075 | 0.075 | 0.000 | 0.000 | 1300 | 1.33343 | 1300 | 1.33428 |
| 0.950 | 0.950 | 0.025 | 0.025 | 0.000 | 0.000 | 1300 | 1.33346 | 1300 | 1.33468 |
| 0.050 | 0.050 | 0.475 | 0.000 | 0.475 | 0.000 | 1300 | 1.25678 | 1300 | 1.26402 |
| 0.150 | 0.150 | 0.425 | 0.000 | 0.425 | 0.000 | 1300 | 1.26759 | 1300 | 1.28124 |
| 0.250 | 0.250 | 0.375 | 0.000 | 0.375 | 0.000 | 1300 | 1.28441 | 1300 | 1.30253 |
| 0.350 | 0.350 | 0.325 | 0.000 | 0.325 | 0.000 | 1300 | 1.30788 | 1300 | 1.32719 |
| 0.450 | 0.450 | 0.275 | 0.000 | 0.275 | 0.000 | 1300 | 1.33334 | 1300 | 1.33317 |
| 0.550 | 0.550 | 0.225 | 0.000 | 0.225 | 0.000 | 1300 | 1.33334 | 1300 | 1.33323 |
| 0.650 | 0.650 | 0.175 | 0.000 | 0.175 | 0.000 | 1300 | 1.33336 | 1300 | 1.33341 |
| 0.750 | 0.750 | 0.125 | 0.000 | 0.125 | 0.000 | 1300 | 1.33338 | 1300 | 1.33369 |
| 0.850 | 0.850 | 0.075 | 0.000 | 0.075 | 0.000 | 1300 | 1.33341 | 1300 | 1.33409 |
| 0.950 | 0.950 | 0.025 | 0.000 | 0.025 | 0.000 | 1300 | 1.33346 | 1300 | 1.33463 |
| 0.050 | 0.050 | 0.475 | 0.000 | 0.000 | 0.475 | 1300 | 1.25343 | 1300 | 1.25742 |
| 0.150 | 0.150 | 0.425 | 0.000 | 0.000 | 0.425 | 1300 | 1.26689 | 1300 | 1.27706 |
| 0.250 | 0.250 | 0.375 | 0.000 | 0.000 | 0.375 | 1300 | 1.28708 | 1300 | 1.29996 |
| 0.350 | 0.350 | 0.325 | 0.000 | 0.000 | 0.325 | 1300 | 1.31046 | 1300 | 1.32423 |
| 0.450 | 0.450 | 0.275 | 0.000 | 0.000 | 0.275 | 1300 | 1.33334 | 1300 | 1.33290 |
| 0.550 | 0.550 | 0.225 | 0.000 | 0.000 | 0.225 | 1300 | 1.33336 | 1300 | 1.33323 |
| 0.650 | 0.650 | 0.175 | 0.000 | 0.000 | 0.175 | 1300 | 1.33338 | 1300 | 1.33352 |
| 0.750 | 0.750 | 0.125 | 0.000 | 0.000 | 0.125 | 1300 | 1.33340 | 1300 | 1.33381 |
| 0.850 | 0.850 | 0.075 | 0.000 | 0.000 | 0.075 | 1300 | 1.33342 | 1300 | 1.33416 |
| 0.950 | 0.950 | 0.025 | 0.000 | 0.000 | 0.025 | 1300 | 1.33346 | 1300 | 1.33464 |
| 0.050 | 0.050 | 0.000 | 0.475 | 0.475 | 0.000 | 1300 | 1.01114 | 1300 | 1.01696 |
| 0.150 | 0.150 | 0.000 | 0.425 | 0.425 | 0.000 | 1300 | 1.03894 | 1300 | 1.05333 |
| 0.250 | 0.250 | 0.000 | 0.375 | 0.375 | 0.000 | 1300 | 1.06970 | 1300 | 1.08879 |
| 0.350 | 0.350 | 0.000 | 0.325 | 0.325 | 0.000 | 1300 | 1.09893 | 1300 | 1.12130 |
| 0.450 | 0.450 | 0.000 | 0.275 | 0.275 | 0.000 | 1300 | 1.12511 | 1300 | 1.16395 |
| 0.550 | 0.550 | 0.000 | 0.225 | 0.225 | 0.000 | 1300 | 1.18320 | 1300 | 1.22292 |
| 0.650 | 0.650 | 0.000 | 0.175 | 0.175 | 0.000 | 1300 | 1.25146 | 1300 | 1.28583 |
| 0.750 | 0.750 | 0.000 | 0.125 | 0.125 | 0.000 | 1300 | 1.31954 | 1300 | 1.33305 |
| 0.850 | 0.850 | 0.000 | 0.075 | 0.075 | 0.000 | 1300 | 1.33335 | 1300 | 1.33329 |
| 0.950 | 0.950 | 0.000 | 0.025 | 0.025 | 0.000 | 1300 | 1.33342 | 1300 | 1.33415 |
| 0.050 | 0.050 | 0.000 | 0.475 | 0.000 | 0.475 | 1300 | 1.00721 | 1300 | 1.01273 |
| 0.150 | 0.150 | 0.000 | 0.425 | 0.000 | 0.425 | 1300 | 1.03178 | 1300 | 1.04730 |
| 0.250 | 0.250 | 0.000 | 0.375 | 0.000 | 0.375 | 1300 | 1.06641 | 1300 | 1.08606 |
| 0.350 | 0.350 | 0.000 | 0.325 | 0.000 | 0.325 | 1300 | 1.10075 | 1300 | 1.12502 |
| 0.450 | 0.450 | 0.000 | 0.275 | 0.000 | 0.275 | 1300 | 1.13227 | 1300 | 1.16978 |
| 0.550 | 0.550 | 0.000 | 0.225 | 0.000 | 0.225 | 1300 | 1.19303 | 1300 | 1.22720 |
| 0.650 | 0.650 | 0.000 | 0.175 | 0.000 | 0.175 | 1300 | 1.25368 | 1300 | 1.28420 |
| 0.750 | 0.750 | 0.000 | 0.125 | 0.000 | 0.125 | 1300 | 1.31414 | 1300 | 1.33282 |
| 0.850 | 0.850 | 0.000 | 0.075 | 0.000 | 0.075 | 1300 | 1.33336 | 1300 | 1.33327 |
| 0.950 | 0.950 | 0.000 | 0.025 | 0.000 | 0.025 | 1300 | 1.33342 | 1300 | 1.33415 |
| 0.050 | 0.050 | 0.000 | 0.000 | 0.475 | 0.475 | 1300 | 1.01010 | 1300 | 1.01503 |
| 0.150 | 0.150 | 0.000 | 0.000 | 0.425 | 0.425 | 1300 | 1.03467 | 1300 | 1.04612 |
| 0.250 | 0.250 | 0.000 | 0.000 | 0.375 | 0.375 | 1300 | 1.06175 | 1300 | 1.07676 |
| 0.350 | 0.350 | 0.000 | 0.000 | 0.325 | 0.325 | 1300 | 1.08883 | 1300 | 1.11748 |
| 0.450 | 0.450 | 0.000 | 0.000 | 0.275 | 0.275 | 1300 | 1.12767 | 1300 | 1.16803 |
| 0.550 | 0.550 | 0.000 | 0.000 | 0.225 | 0.225 | 1300 | 1.19661 | 1300 | 1.23233 |
| 0.650 | 0.650 | 0.000 | 0.000 | 0.175 | 0.175 | 1300 | 1.26557 | 1300 | 1.29580 |
| 0.750 | 0.750 | 0.000 | 0.000 | 0.125 | 0.125 | 1300 | 1.33333 | 1300 | 1.33295 |
| 0.850 | 0.850 | 0.000 | 0.000 | 0.075 | 0.075 | 1300 | 1.33335 | 1300 | 1.33323 |
| 0.950 | 0.950 | 0.000 | 0.000 | 0.025 | 0.025 | 1300 | 1.33342 | 1300 | 1.33413 |
| 0.050 | 0.050 | 0.317 | 0.317 | 0.317 | 0.000 | 1300 | 1.17469 | 1300 | 1.18063 |
| 0.150 | 0.150 | 0.283 | 0.283 | 0.283 | 0.000 | 1300 | 1.19322 | 1300 | 1.20645 |
| 0.250 | 0.250 | 0.250 | 0.250 | 0.250 | 0.000 | 1300 | 1.21693 | 1300 | 1.23608 |
| 0.350 | 0.350 | 0.217 | 0.217 | 0.217 | 0.000 | 1300 | 1.24965 | 1300 | 1.26983 |
| 0.450 | 0.450 | 0.183 | 0.183 | 0.183 | 0.000 | 1300 | 1.28486 | 1300 | 1.30430 |
| 0.550 | 0.550 | 0.150 | 0.150 | 0.150 | 0.000 | 1300 | 1.32016 | 1300 | 1.33311 |
| 0.650 | 0.650 | 0.117 | 0.117 | 0.117 | 0.000 | 1300 | 1.33334 | 1300 | 1.33316 |
| 0.750 | 0.750 | 0.083 | 0.083 | 0.083 | 0.000 | 1300 | 1.33336 | 1300 | 1.33337 |
| 0.850 | 0.850 | 0.050 | 0.050 | 0.050 | 0.000 | 1300 | 1.33339 | 1300 | 1.33379 |
| 0.950 | 0.950 | 0.017 | 0.017 | 0.017 | 0.000 | 1300 | 1.33345 | 1300 | 1.33448 |
| 0.050 | 0.050 | 0.317 | 0.317 | 0.000 | 0.317 | 1300 | 1.17266 | 1300 | 1.17748 |
| 0.150 | 0.150 | 0.283 | 0.283 | 0.000 | 0.283 | 1300 | 1.19259 | 1300 | 1.20470 |
| 0.250 | 0.250 | 0.250 | 0.250 | 0.000 | 0.250 | 1300 | 1.22082 | 1300 | 1.23622 |
| 0.350 | 0.350 | 0.217 | 0.217 | 0.000 | 0.217 | 1300 | 1.25299 | 1300 | 1.26911 |
| 0.450 | 0.450 | 0.183 | 0.183 | 0.000 | 0.183 | 1300 | 1.28550 | 1300 | 1.30195 |
| 0.550 | 0.550 | 0.150 | 0.150 | 0.000 | 0.150 | 1300 | 1.31772 | 1300 | 1.33278 |
| 0.650 | 0.650 | 0.117 | 0.117 | 0.000 | 0.117 | 1300 | 1.33334 | 1300 | 1.33310 |
| 0.750 | 0.750 | 0.083 | 0.083 | 0.000 | 0.083 | 1300 | 1.33336 | 1300 | 1.33340 |
| 0.850 | 0.850 | 0.050 | 0.050 | 0.000 | 0.050 | 1300 | 1.33340 | 1300 | 1.33382 |
| 0.950 | 0.950 | 0.017 | 0.017 | 0.000 | 0.017 | 1300 | 1.33345 | 1300 | 1.33448 |
| 0.050 | 0.050 | 0.317 | 0.000 | 0.317 | 0.317 | 1300 | 1.17414 | 1300 | 1.17947 |
| 0.150 | 0.150 | 0.283 | 0.000 | 0.283 | 0.283 | 1300 | 1.19312 | 1300 | 1.20551 |
| 0.250 | 0.250 | 0.250 | 0.000 | 0.250 | 0.250 | 1300 | 1.21936 | 1300 | 1.23623 |
| 0.350 | 0.350 | 0.217 | 0.000 | 0.217 | 0.217 | 1300 | 1.25203 | 1300 | 1.27009 |
| 0.450 | 0.450 | 0.183 | 0.000 | 0.183 | 0.183 | 1300 | 1.28738 | 1300 | 1.30515 |
| 0.550 | 0.550 | 0.150 | 0.000 | 0.150 | 0.150 | 1300 | 1.32403 | 1300 | 1.33289 |
| 0.650 | 0.650 | 0.117 | 0.000 | 0.117 | 0.117 | 1300 | 1.33334 | 1300 | 1.33306 |
| 0.750 | 0.750 | 0.083 | 0.000 | 0.083 | 0.083 | 1300 | 1.33336 | 1300 | 1.33329 |
| 0.850 | 0.850 | 0.050 | 0.000 | 0.050 | 0.050 | 1300 | 1.33339 | 1300 | 1.33373 |
| 0.950 | 0.950 | 0.017 | 0.000 | 0.017 | 0.017 | 1300 | 1.33345 | 1300 | 1.33445 |
| 0.050 | 0.050 | 0.000 | 0.317 | 0.317 | 0.317 | 1300 | 1.00993 | 1300 | 1.01571 |
| 0.150 | 0.150 | 0.000 | 0.283 | 0.283 | 0.283 | 1300 | 1.03867 | 1300 | 1.05262 |
| 0.250 | 0.250 | 0.000 | 0.250 | 0.250 | 0.250 | 1300 | 1.07115 | 1300 | 1.08883 |
| 0.350 | 0.350 | 0.000 | 0.217 | 0.217 | 0.217 | 1300 | 1.10103 | 1300 | 1.12470 |
| 0.450 | 0.450 | 0.000 | 0.183 | 0.183 | 0.183 | 1300 | 1.12842 | 1300 | 1.16868 |
| 0.550 | 0.550 | 0.000 | 0.150 | 0.150 | 0.150 | 1300 | 1.19387 | 1300 | 1.22993 |
| 0.650 | 0.650 | 0.000 | 0.117 | 0.117 | 0.117 | 1300 | 1.25892 | 1300 | 1.29014 |
| 0.750 | 0.750 | 0.000 | 0.083 | 0.083 | 0.083 | 1300 | 1.32307 | 1300 | 1.33295 |
| 0.850 | 0.850 | 0.000 | 0.050 | 0.050 | 0.050 | 1300 | 1.33335 | 1300 | 1.33326 |
| 0.950 | 0.950 | 0.000 | 0.017 | 0.017 | 0.017 | 1300 | 1.33342 | 1300 | 1.33414 |
| 0.050 | 0.050 | 0.238 | 0.238 | 0.238 | 0.238 | 1300 | 1.13291 | 1300 | 1.13848 |
| 0.150 | 0.150 | 0.213 | 0.213 | 0.213 | 0.213 | 1300 | 1.15503 | 1300 | 1.16796 |
| 0.250 | 0.250 | 0.188 | 0.188 | 0.188 | 0.188 | 1300 | 1.18307 | 1300 | 1.20162 |
| 0.350 | 0.350 | 0.163 | 0.163 | 0.163 | 0.163 | 1300 | 1.21882 | 1300 | 1.23893 |
| 0.450 | 0.450 | 0.138 | 0.138 | 0.138 | 0.138 | 1300 | 1.25697 | 1300 | 1.27711 |
| 0.550 | 0.550 | 0.113 | 0.113 | 0.113 | 0.113 | 1300 | 1.29576 | 1300 | 1.31555 |
| 0.650 | 0.650 | 0.088 | 0.088 | 0.088 | 0.088 | 1300 | 1.33334 | 1300 | 1.33303 |
| 0.750 | 0.750 | 0.063 | 0.063 | 0.063 | 0.063 | 1300 | 1.33335 | 1300 | 1.33320 |
| 0.850 | 0.850 | 0.038 | 0.038 | 0.038 | 0.038 | 1300 | 1.33338 | 1300 | 1.33361 |
| 0.950 | 0.950 | 0.013 | 0.013 | 0.013 | 0.013 | 1300 | 1.33344 | 1300 | 1.33438 |
| 0.050 | 0.050 | 0.950 | 0.000 | 0.000 | 0.000 | 1300 | 1.48884 | 1100 | 1.49937 |
| 0.150 | 0.150 | 0.850 | 0.000 | 0.000 | 0.000 | 1300 | 1.44815 | 1100 | 1.46506 |
| 0.250 | 0.250 | 0.750 | 0.000 | 0.000 | 0.000 | 1300 | 1.40746 | 1100 | 1.42817 |
| 0.350 | 0.350 | 0.650 | 0.000 | 0.000 | 0.000 | 1300 | 1.36677 | 1100 | 1.39034 |
| 0.450 | 0.450 | 0.550 | 0.000 | 0.000 | 0.000 | 1300 | 1.33570 | 1100 | 1.35790 |
| 0.550 | 0.550 | 0.450 | 0.000 | 0.000 | 0.000 | 1300 | 1.33412 | 1100 | 1.34837 |
| 0.650 | 0.650 | 0.350 | 0.000 | 0.000 | 0.000 | 1300 | 1.33378 | 1100 | 1.34380 |
| 0.750 | 0.750 | 0.250 | 0.000 | 0.000 | 0.000 | 1300 | 1.33364 | 1100 | 1.34002 |
| 0.850 | 0.850 | 0.150 | 0.000 | 0.000 | 0.000 | 1300 | 1.33357 | 1100 | 1.33704 |
| 0.950 | 0.950 | 0.050 | 0.000 | 0.000 | 0.000 | 1300 | 1.33351 | 1100 | 1.33673 |
| 0.050 | 0.050 | 0.000 | 0.950 | 0.000 | 0.000 | 1300 | 1.00743 | 1100 | 1.01523 |
| 0.150 | 0.150 | 0.000 | 0.850 | 0.000 | 0.000 | 1300 | 1.02872 | 1100 | 1.05151 |
| 0.250 | 0.250 | 0.000 | 0.750 | 0.000 | 0.000 | 1300 | 1.05920 | 1100 | 1.09534 |
| 0.350 | 0.350 | 0.000 | 0.650 | 0.000 | 0.000 | 1300 | 1.09416 | 1100 | 1.14129 |
| 0.450 | 0.450 | 0.000 | 0.550 | 0.000 | 0.000 | 1300 | 1.12893 | 1100 | 1.18773 |
| 0.550 | 0.550 | 0.000 | 0.450 | 0.000 | 0.000 | 1300 | 1.19093 | 1100 | 1.24090 |
| 0.650 | 0.650 | 0.000 | 0.350 | 0.000 | 0.000 | 1300 | 1.25293 | 1100 | 1.29409 |
| 0.750 | 0.750 | 0.000 | 0.250 | 0.000 | 0.000 | 1300 | 1.31493 | 1100 | 1.33330 |
| 0.850 | 0.850 | 0.000 | 0.150 | 0.000 | 0.000 | 1300 | 1.33336 | 1100 | 1.33420 |
| 0.950 | 0.950 | 0.000 | 0.050 | 0.000 | 0.000 | 1300 | 1.33342 | 1100 | 1.33565 |
| 0.050 | 0.050 | 0.000 | 0.000 | 0.950 | 0.000 | 1300 | 1.01369 | 1100 | 1.02032 |
| 0.150 | 0.150 | 0.000 | 0.000 | 0.850 | 0.000 | 1300 | 1.03957 | 1100 | 1.05318 |
| 0.250 | 0.250 | 0.000 | 0.000 | 0.750 | 0.000 | 1300 | 1.06413 | 1100 | 1.08446 |
| 0.350 | 0.350 | 0.000 | 0.000 | 0.650 | 0.000 | 1300 | 1.08781 | 1100 | 1.12616 |
| 0.450 | 0.450 | 0.000 | 0.000 | 0.550 | 0.000 | 1300 | 1.11087 | 1100 | 1.17175 |
| 0.550 | 0.550 | 0.000 | 0.000 | 0.450 | 0.000 | 1300 | 1.17084 | 1100 | 1.23139 |
| 0.650 | 0.650 | 0.000 | 0.000 | 0.350 | 0.000 | 1300 | 1.24407 | 1100 | 1.29426 |
| 0.750 | 0.750 | 0.000 | 0.000 | 0.250 | 0.000 | 1300 | 1.31731 | 1100 | 1.33331 |
| 0.850 | 0.850 | 0.000 | 0.000 | 0.150 | 0.000 | 1300 | 1.33335 | 1100 | 1.33412 |
| 0.950 | 0.950 | 0.000 | 0.000 | 0.050 | 0.000 | 1300 | 1.33342 | 1100 | 1.33563 |
| 0.050 | 0.050 | 0.000 | 0.000 | 0.000 | 0.950 | 1300 | 1.00610 | 1100 | 1.01281 |
| 0.150 | 0.150 | 0.000 | 0.000 | 0.000 | 0.850 | 1300 | 1.02458 | 1100 | 1.04382 |
| 0.250 | 0.250 | 0.000 | 0.000 | 0.000 | 0.750 | 1300 | 1.05012 | 1100 | 1.08612 |
| 0.350 | 0.350 | 0.000 | 0.000 | 0.000 | 0.650 | 1300 | 1.07913 | 1100 | 1.13188 |
| 0.450 | 0.450 | 0.000 | 0.000 | 0.000 | 0.550 | 1300 | 1.13526 | 1100 | 1.18561 |
| 0.550 | 0.550 | 0.000 | 0.000 | 0.000 | 0.450 | 1300 | 1.19622 | 1100 | 1.24039 |
| 0.650 | 0.650 | 0.000 | 0.000 | 0.000 | 0.350 | 1300 | 1.25718 | 1100 | 1.29499 |
| 0.750 | 0.750 | 0.000 | 0.000 | 0.000 | 0.250 | 1300 | 1.31814 | 1100 | 1.33328 |
| 0.850 | 0.850 | 0.000 | 0.000 | 0.000 | 0.150 | 1300 | 1.33335 | 1100 | 1.33410 |
| 0.950 | 0.950 | 0.000 | 0.000 | 0.000 | 0.050 | 1300 | 1.33342 | 1100 | 1.33563 |
| 0.050 | 0.050 | 0.475 | 0.475 | 0.000 | 0.000 | 1300 | 1.25677 | 1100 | 1.26123 |
| 0.150 | 0.150 | 0.425 | 0.425 | 0.000 | 0.000 | 1300 | 1.27496 | 1100 | 1.28430 |
| 0.250 | 0.250 | 0.375 | 0.375 | 0.000 | 0.000 | 1300 | 1.29812 | 1100 | 1.30881 |
| 0.350 | 0.350 | 0.325 | 0.325 | 0.000 | 0.000 | 1300 | 1.32274 | 1100 | 1.33331 |
| 0.450 | 0.450 | 0.275 | 0.275 | 0.000 | 0.000 | 1300 | 1.33334 | 1100 | 1.33350 |
| 0.550 | 0.550 | 0.225 | 0.225 | 0.000 | 0.000 | 1300 | 1.33336 | 1100 | 1.33398 |
| 0.650 | 0.650 | 0.175 | 0.175 | 0.000 | 0.000 | 1300 | 1.33338 | 1100 | 1.33456 |
| 0.750 | 0.750 | 0.125 | 0.125 | 0.000 | 0.000 | 1300 | 1.33340 | 1100 | 1.33509 |
| 0.850 | 0.850 | 0.075 | 0.075 | 0.000 | 0.000 | 1300 | 1.33343 | 1100 | 1.33560 |
| 0.950 | 0.950 | 0.025 | 0.025 | 0.000 | 0.000 | 1300 | 1.33346 | 1100 | 1.33620 |
| 0.050 | 0.050 | 0.475 | 0.000 | 0.475 | 0.000 | 1300 | 1.25678 | 1100 | 1.26430 |
| 0.150 | 0.150 | 0.425 | 0.000 | 0.425 | 0.000 | 1300 | 1.26759 | 1100 | 1.28251 |
| 0.250 | 0.250 | 0.375 | 0.000 | 0.375 | 0.000 | 1300 | 1.28441 | 1100 | 1.30475 |
| 0.350 | 0.350 | 0.325 | 0.000 | 0.325 | 0.000 | 1300 | 1.30788 | 1100 | 1.32986 |
| 0.450 | 0.450 | 0.275 | 0.000 | 0.275 | 0.000 | 1300 | 1.33334 | 1100 | 1.33338 |
| 0.550 | 0.550 | 0.225 | 0.000 | 0.225 | 0.000 | 1300 | 1.33334 | 1100 | 1.33361 |
| 0.650 | 0.650 | 0.175 | 0.000 | 0.175 | 0.000 | 1300 | 1.33336 | 1100 | 1.33407 |
| 0.750 | 0.750 | 0.125 | 0.000 | 0.125 | 0.000 | 1300 | 1.33338 | 1100 | 1.33470 |
| 0.850 | 0.850 | 0.075 | 0.000 | 0.075 | 0.000 | 1300 | 1.33341 | 1100 | 1.33542 |
| 0.950 | 0.950 | 0.025 | 0.000 | 0.025 | 0.000 | 1300 | 1.33346 | 1100 | 1.33617 |
| 0.050 | 0.050 | 0.475 | 0.000 | 0.000 | 0.475 | 1300 | 1.25343 | 1100 | 1.25993 |
| 0.150 | 0.150 | 0.425 | 0.000 | 0.000 | 0.425 | 1300 | 1.26689 | 1100 | 1.28068 |
| 0.250 | 0.250 | 0.375 | 0.000 | 0.000 | 0.375 | 1300 | 1.28708 | 1100 | 1.30409 |
| 0.350 | 0.350 | 0.325 | 0.000 | 0.000 | 0.325 | 1300 | 1.31046 | 1100 | 1.32863 |
| 0.450 | 0.450 | 0.275 | 0.000 | 0.000 | 0.275 | 1300 | 1.33334 | 1100 | 1.33354 |
| 0.550 | 0.550 | 0.225 | 0.000 | 0.000 | 0.225 | 1300 | 1.33336 | 1100 | 1.33399 |
| 0.650 | 0.650 | 0.175 | 0.000 | 0.000 | 0.175 | 1300 | 1.33338 | 1100 | 1.33446 |
| 0.750 | 0.750 | 0.125 | 0.000 | 0.000 | 0.125 | 1300 | 1.33340 | 1100 | 1.33496 |
| 0.850 | 0.850 | 0.075 | 0.000 | 0.000 | 0.075 | 1300 | 1.33342 | 1100 | 1.33552 |
| 0.950 | 0.950 | 0.025 | 0.000 | 0.000 | 0.025 | 1300 | 1.33346 | 1100 | 1.33618 |
| 0.050 | 0.050 | 0.000 | 0.475 | 0.475 | 0.000 | 1300 | 1.01114 | 1100 | 1.01903 |
| 0.150 | 0.150 | 0.000 | 0.425 | 0.425 | 0.000 | 1300 | 1.03894 | 1100 | 1.05594 |
| 0.250 | 0.250 | 0.000 | 0.375 | 0.375 | 0.000 | 1300 | 1.06970 | 1100 | 1.09355 |
| 0.350 | 0.350 | 0.000 | 0.325 | 0.325 | 0.000 | 1300 | 1.09893 | 1100 | 1.13810 |
| 0.450 | 0.450 | 0.000 | 0.275 | 0.275 | 0.000 | 1300 | 1.12511 | 1100 | 1.18417 |
| 0.550 | 0.550 | 0.000 | 0.225 | 0.225 | 0.000 | 1300 | 1.18320 | 1100 | 1.24076 |
| 0.650 | 0.650 | 0.000 | 0.175 | 0.175 | 0.000 | 1300 | 1.25146 | 1100 | 1.29941 |
| 0.750 | 0.750 | 0.000 | 0.125 | 0.125 | 0.000 | 1300 | 1.31954 | 1100 | 1.33331 |
| 0.850 | 0.850 | 0.000 | 0.075 | 0.075 | 0.000 | 1300 | 1.33335 | 1100 | 1.33410 |
| 0.950 | 0.950 | 0.000 | 0.025 | 0.025 | 0.000 | 1300 | 1.33342 | 1100 | 1.33563 |
| 0.050 | 0.050 | 0.000 | 0.475 | 0.000 | 0.475 | 1300 | 1.00721 | 1100 | 1.01497 |
| 0.150 | 0.150 | 0.000 | 0.425 | 0.000 | 0.425 | 1300 | 1.03178 | 1100 | 1.05087 |
| 0.250 | 0.250 | 0.000 | 0.375 | 0.000 | 0.375 | 1300 | 1.06641 | 1100 | 1.09597 |
| 0.350 | 0.350 | 0.000 | 0.325 | 0.000 | 0.325 | 1300 | 1.10075 | 1100 | 1.14192 |
| 0.450 | 0.450 | 0.000 | 0.275 | 0.000 | 0.275 | 1300 | 1.13227 | 1100 | 1.18797 |
| 0.550 | 0.550 | 0.000 | 0.225 | 0.000 | 0.225 | 1300 | 1.19303 | 1100 | 1.24133 |
| 0.650 | 0.650 | 0.000 | 0.175 | 0.000 | 0.175 | 1300 | 1.25368 | 1100 | 1.29451 |
| 0.750 | 0.750 | 0.000 | 0.125 | 0.000 | 0.125 | 1300 | 1.31414 | 1100 | 1.33329 |
| 0.850 | 0.850 | 0.000 | 0.075 | 0.000 | 0.075 | 1300 | 1.33336 | 1100 | 1.33416 |
| 0.950 | 0.950 | 0.000 | 0.025 | 0.000 | 0.025 | 1300 | 1.33342 | 1100 | 1.33565 |
| 0.050 | 0.050 | 0.000 | 0.000 | 0.475 | 0.475 | 1300 | 1.01010 | 1100 | 1.01727 |
| 0.150 | 0.150 | 0.000 | 0.000 | 0.425 | 0.425 | 1300 | 1.03467 | 1100 | 1.04855 |
| 0.250 | 0.250 | 0.000 | 0.000 | 0.375 | 0.375 | 1300 | 1.06175 | 1100 | 1.09052 |
| 0.350 | 0.350 | 0.000 | 0.000 | 0.325 | 0.325 | 1300 | 1.08883 | 1100 | 1.13660 |
| 0.450 | 0.450 | 0.000 | 0.000 | 0.275 | 0.275 | 1300 | 1.12767 | 1100 | 1.18852 |
| 0.550 | 0.550 | 0.000 | 0.000 | 0.225 | 0.225 | 1300 | 1.19661 | 1100 | 1.24843 |
| 0.650 | 0.650 | 0.000 | 0.000 | 0.175 | 0.175 | 1300 | 1.26557 | 1100 | 1.30756 |
| 0.750 | 0.750 | 0.000 | 0.000 | 0.125 | 0.125 | 1300 | 1.33333 | 1100 | 1.33339 |
| 0.850 | 0.850 | 0.000 | 0.000 | 0.075 | 0.075 | 1300 | 1.33335 | 1100 | 1.33409 |
| 0.950 | 0.950 | 0.000 | 0.000 | 0.025 | 0.025 | 1300 | 1.33342 | 1100 | 1.33563 |
| 0.050 | 0.050 | 0.317 | 0.317 | 0.317 | 0.000 | 1300 | 1.17469 | 1100 | 1.18179 |
| 0.150 | 0.150 | 0.283 | 0.283 | 0.283 | 0.000 | 1300 | 1.19322 | 1100 | 1.20891 |
| 0.250 | 0.250 | 0.250 | 0.250 | 0.250 | 0.000 | 1300 | 1.21693 | 1100 | 1.24020 |
| 0.350 | 0.350 | 0.217 | 0.217 | 0.217 | 0.000 | 1300 | 1.24965 | 1100 | 1.27442 |
| 0.450 | 0.450 | 0.183 | 0.183 | 0.183 | 0.000 | 1300 | 1.28486 | 1100 | 1.30884 |
| 0.550 | 0.550 | 0.150 | 0.150 | 0.150 | 0.000 | 1300 | 1.32016 | 1100 | 1.33331 |
| 0.650 | 0.650 | 0.117 | 0.117 | 0.117 | 0.000 | 1300 | 1.33334 | 1100 | 1.33355 |
| 0.750 | 0.750 | 0.083 | 0.083 | 0.083 | 0.000 | 1300 | 1.33336 | 1100 | 1.33412 |
| 0.850 | 0.850 | 0.050 | 0.050 | 0.050 | 0.000 | 1300 | 1.33339 | 1100 | 1.33498 |
| 0.950 | 0.950 | 0.017 | 0.017 | 0.017 | 0.000 | 1300 | 1.33345 | 1100 | 1.33600 |
| 0.050 | 0.050 | 0.317 | 0.317 | 0.000 | 0.317 | 1300 | 1.17266 | 1100 | 1.17978 |
| 0.150 | 0.150 | 0.283 | 0.283 | 0.000 | 0.283 | 1300 | 1.19259 | 1100 | 1.20818 |
| 0.250 | 0.250 | 0.250 | 0.250 | 0.000 | 0.250 | 1300 | 1.22082 | 1100 | 1.24036 |
| 0.350 | 0.350 | 0.217 | 0.217 | 0.000 | 0.217 | 1300 | 1.25299 | 1100 | 1.27338 |
| 0.450 | 0.450 | 0.183 | 0.183 | 0.000 | 0.183 | 1300 | 1.28550 | 1100 | 1.30611 |
| 0.550 | 0.550 | 0.150 | 0.150 | 0.000 | 0.150 | 1300 | 1.31772 | 1100 | 1.33329 |
| 0.650 | 0.650 | 0.117 | 0.117 | 0.000 | 0.117 | 1300 | 1.33334 | 1100 | 1.33367 |
| 0.750 | 0.750 | 0.083 | 0.083 | 0.000 | 0.083 | 1300 | 1.33336 | 1100 | 1.33430 |
| 0.850 | 0.850 | 0.050 | 0.050 | 0.000 | 0.050 | 1300 | 1.33340 | 1100 | 1.33507 |
| 0.950 | 0.950 | 0.017 | 0.017 | 0.000 | 0.017 | 1300 | 1.33345 | 1100 | 1.33601 |
| 0.050 | 0.050 | 0.317 | 0.000 | 0.317 | 0.317 | 1300 | 1.17414 | 1100 | 1.18144 |
| 0.150 | 0.150 | 0.283 | 0.000 | 0.283 | 0.283 | 1300 | 1.19312 | 1100 | 1.20914 |
| 0.250 | 0.250 | 0.250 | 0.000 | 0.250 | 0.250 | 1300 | 1.21936 | 1100 | 1.24101 |
| 0.350 | 0.350 | 0.217 | 0.000 | 0.217 | 0.217 | 1300 | 1.25203 | 1100 | 1.27519 |
| 0.450 | 0.450 | 0.183 | 0.000 | 0.183 | 0.183 | 1300 | 1.28738 | 1100 | 1.31007 |
| 0.550 | 0.550 | 0.150 | 0.000 | 0.150 | 0.150 | 1300 | 1.32403 | 1100 | 1.33330 |
| 0.650 | 0.650 | 0.117 | 0.000 | 0.117 | 0.117 | 1300 | 1.33334 | 1100 | 1.33352 |
| 0.750 | 0.750 | 0.083 | 0.000 | 0.083 | 0.083 | 1300 | 1.33336 | 1100 | 1.33408 |
| 0.850 | 0.850 | 0.050 | 0.000 | 0.050 | 0.050 | 1300 | 1.33339 | 1100 | 1.33496 |
| 0.950 | 0.950 | 0.017 | 0.000 | 0.017 | 0.017 | 1300 | 1.33345 | 1100 | 1.33599 |
| 0.050 | 0.050 | 0.000 | 0.317 | 0.317 | 0.317 | 1300 | 1.00993 | 1100 | 1.01790 |
| 0.150 | 0.150 | 0.000 | 0.283 | 0.283 | 0.283 | 1300 | 1.03867 | 1100 | 1.05500 |
| 0.250 | 0.250 | 0.000 | 0.250 | 0.250 | 0.250 | 1300 | 1.07115 | 1100 | 1.09625 |
| 0.350 | 0.350 | 0.000 | 0.217 | 0.217 | 0.217 | 1300 | 1.10103 | 1100 | 1.14193 |
| 0.450 | 0.450 | 0.000 | 0.183 | 0.183 | 0.183 | 1300 | 1.12842 | 1100 | 1.18901 |
| 0.550 | 0.550 | 0.000 | 0.150 | 0.150 | 0.150 | 1300 | 1.19387 | 1100 | 1.24628 |
| 0.650 | 0.650 | 0.000 | 0.117 | 0.117 | 0.117 | 1300 | 1.25892 | 1100 | 1.30250 |
| 0.750 | 0.750 | 0.000 | 0.083 | 0.083 | 0.083 | 1300 | 1.32307 | 1100 | 1.33331 |
| 0.850 | 0.850 | 0.000 | 0.050 | 0.050 | 0.050 | 1300 | 1.33335 | 1100 | 1.33411 |
| 0.950 | 0.950 | 0.000 | 0.017 | 0.017 | 0.017 | 1300 | 1.33342 | 1100 | 1.33563 |
| 0.050 | 0.050 | 0.238 | 0.238 | 0.238 | 0.238 | 1300 | 1.13291 | 1100 | 1.14044 |
| 0.150 | 0.150 | 0.213 | 0.213 | 0.213 | 0.213 | 1300 | 1.15503 | 1100 | 1.17164 |
| 0.250 | 0.250 | 0.188 | 0.188 | 0.188 | 0.188 | 1300 | 1.18307 | 1100 | 1.20726 |
| 0.350 | 0.350 | 0.163 | 0.163 | 0.163 | 0.163 | 1300 | 1.21882 | 1100 | 1.24514 |
| 0.450 | 0.450 | 0.138 | 0.138 | 0.138 | 0.138 | 1300 | 1.25697 | 1100 | 1.28332 |
| 0.550 | 0.550 | 0.113 | 0.113 | 0.113 | 0.113 | 1300 | 1.29576 | 1100 | 1.32138 |
| 0.650 | 0.650 | 0.088 | 0.088 | 0.088 | 0.088 | 1300 | 1.33334 | 1100 | 1.33340 |
| 0.750 | 0.750 | 0.063 | 0.063 | 0.063 | 0.063 | 1300 | 1.33335 | 1100 | 1.33386 |
| 0.850 | 0.850 | 0.038 | 0.038 | 0.038 | 0.038 | 1300 | 1.33338 | 1100 | 1.33475 |
| 0.950 | 0.950 | 0.013 | 0.013 | 0.013 | 0.013 | 1300 | 1.33344 | 1100 | 1.33591 |
| 0.050 | 0.050 | 0.950 | 0.000 | 0.000 | 0.000 | 1300 | 1.48884 | 800 | 1.49568 |
| 0.150 | 0.150 | 0.850 | 0.000 | 0.000 | 0.000 | 1300 | 1.44815 | 800 | 1.45715 |
| 0.250 | 0.250 | 0.750 | 0.000 | 0.000 | 0.000 | 1300 | 1.40746 | 800 | 1.41763 |
| 0.350 | 0.350 | 0.650 | 0.000 | 0.000 | 0.000 | 1300 | 1.36677 | 800 | 1.37775 |
| 0.450 | 0.450 | 0.550 | 0.000 | 0.000 | 0.000 | 1300 | 1.33570 | 800 | 1.34521 |
| 0.550 | 0.550 | 0.450 | 0.000 | 0.000 | 0.000 | 1300 | 1.33412 | 800 | 1.34073 |
| 0.650 | 0.650 | 0.350 | 0.000 | 0.000 | 0.000 | 1300 | 1.33378 | 800 | 1.34038 |
| 0.750 | 0.750 | 0.250 | 0.000 | 0.000 | 0.000 | 1300 | 1.33364 | 800 | 1.34025 |
| 0.850 | 0.850 | 0.150 | 0.000 | 0.000 | 0.000 | 1300 | 1.33357 | 800 | 1.33746 |
| 0.950 | 0.950 | 0.050 | 0.000 | 0.000 | 0.000 | 1300 | 1.33351 | 800 | 1.33465 |
| 0.050 | 0.050 | 0.000 | 0.950 | 0.000 | 0.000 | 1300 | 1.00743 | 800 | 1.01817 |
| 0.150 | 0.150 | 0.000 | 0.850 | 0.000 | 0.000 | 1300 | 1.02872 | 800 | 1.06197 |
| 0.250 | 0.250 | 0.000 | 0.750 | 0.000 | 0.000 | 1300 | 1.05920 | 800 | 1.10801 |
| 0.350 | 0.350 | 0.000 | 0.650 | 0.000 | 0.000 | 1300 | 1.09416 | 800 | 1.15477 |
| 0.450 | 0.450 | 0.000 | 0.550 | 0.000 | 0.000 | 1300 | 1.12893 | 800 | 1.20152 |
| 0.550 | 0.550 | 0.000 | 0.450 | 0.000 | 0.000 | 1300 | 1.19093 | 800 | 1.25180 |
| 0.650 | 0.650 | 0.000 | 0.350 | 0.000 | 0.000 | 1300 | 1.25293 | 800 | 1.30242 |
| 0.750 | 0.750 | 0.000 | 0.250 | 0.000 | 0.000 | 1300 | 1.31493 | 800 | 1.33333 |
| 0.850 | 0.850 | 0.000 | 0.150 | 0.000 | 0.000 | 1300 | 1.33336 | 800 | 1.33378 |
| 0.950 | 0.950 | 0.000 | 0.050 | 0.000 | 0.000 | 1300 | 1.33342 | 800 | 1.33421 |
| 0.050 | 0.050 | 0.000 | 0.000 | 0.950 | 0.000 | 1300 | 1.01369 | 800 | 1.02103 |
| 0.150 | 0.150 | 0.000 | 0.000 | 0.850 | 0.000 | 1300 | 1.03957 | 800 | 1.05828 |
| 0.250 | 0.250 | 0.000 | 0.000 | 0.750 | 0.000 | 1300 | 1.06413 | 800 | 1.10394 |
| 0.350 | 0.350 | 0.000 | 0.000 | 0.650 | 0.000 | 1300 | 1.08781 | 800 | 1.15209 |
| 0.450 | 0.450 | 0.000 | 0.000 | 0.550 | 0.000 | 1300 | 1.11087 | 800 | 1.20213 |
| 0.550 | 0.550 | 0.000 | 0.000 | 0.450 | 0.000 | 1300 | 1.17084 | 800 | 1.25709 |
| 0.650 | 0.650 | 0.000 | 0.000 | 0.350 | 0.000 | 1300 | 1.24407 | 800 | 1.31291 |
| 0.750 | 0.750 | 0.000 | 0.000 | 0.250 | 0.000 | 1300 | 1.31731 | 800 | 1.33333 |
| 0.850 | 0.850 | 0.000 | 0.000 | 0.150 | 0.000 | 1300 | 1.33335 | 800 | 1.33379 |
| 0.950 | 0.950 | 0.000 | 0.000 | 0.050 | 0.000 | 1300 | 1.33342 | 800 | 1.33420 |
| 0.050 | 0.050 | 0.000 | 0.000 | 0.000 | 0.950 | 1300 | 1.00610 | 800 | 1.01505 |
| 0.150 | 0.150 | 0.000 | 0.000 | 0.000 | 0.850 | 1300 | 1.02458 | 800 | 1.05773 |
| 0.250 | 0.250 | 0.000 | 0.000 | 0.000 | 0.750 | 1300 | 1.05012 | 800 | 1.10467 |
| 0.350 | 0.350 | 0.000 | 0.000 | 0.000 | 0.650 | 1300 | 1.07913 | 800 | 1.15323 |
| 0.450 | 0.450 | 0.000 | 0.000 | 0.000 | 0.550 | 1300 | 1.13526 | 800 | 1.20528 |
| 0.550 | 0.550 | 0.000 | 0.000 | 0.000 | 0.450 | 1300 | 1.19622 | 800 | 1.25777 |
| 0.650 | 0.650 | 0.000 | 0.000 | 0.000 | 0.350 | 1300 | 1.25718 | 800 | 1.31017 |
| 0.750 | 0.750 | 0.000 | 0.000 | 0.000 | 0.250 | 1300 | 1.31814 | 800 | 1.33333 |
| 0.850 | 0.850 | 0.000 | 0.000 | 0.000 | 0.150 | 1300 | 1.33335 | 800 | 1.33377 |
| 0.950 | 0.950 | 0.000 | 0.000 | 0.000 | 0.050 | 1300 | 1.33342 | 800 | 1.33420 |
| 0.050 | 0.050 | 0.475 | 0.475 | 0.000 | 0.000 | 1300 | 1.25677 | 800 | 1.26168 |
| 0.150 | 0.150 | 0.425 | 0.425 | 0.000 | 0.000 | 1300 | 1.27496 | 800 | 1.28513 |
| 0.250 | 0.250 | 0.375 | 0.375 | 0.000 | 0.000 | 1300 | 1.29812 | 800 | 1.30931 |
| 0.350 | 0.350 | 0.325 | 0.325 | 0.000 | 0.000 | 1300 | 1.32274 | 800 | 1.33333 |
| 0.450 | 0.450 | 0.275 | 0.275 | 0.000 | 0.000 | 1300 | 1.33334 | 800 | 1.33350 |
| 0.550 | 0.550 | 0.225 | 0.225 | 0.000 | 0.000 | 1300 | 1.33336 | 800 | 1.33366 |
| 0.650 | 0.650 | 0.175 | 0.175 | 0.000 | 0.000 | 1300 | 1.33338 | 800 | 1.33382 |
| 0.750 | 0.750 | 0.125 | 0.125 | 0.000 | 0.000 | 1300 | 1.33340 | 800 | 1.33399 |
| 0.850 | 0.850 | 0.075 | 0.075 | 0.000 | 0.000 | 1300 | 1.33343 | 800 | 1.33416 |
| 0.950 | 0.950 | 0.025 | 0.025 | 0.000 | 0.000 | 1300 | 1.33346 | 800 | 1.33442 |
| 0.050 | 0.050 | 0.475 | 0.000 | 0.475 | 0.000 | 1300 | 1.25678 | 800 | 1.26271 |
| 0.150 | 0.150 | 0.425 | 0.000 | 0.425 | 0.000 | 1300 | 1.26759 | 800 | 1.28349 |
| 0.250 | 0.250 | 0.375 | 0.000 | 0.375 | 0.000 | 1300 | 1.28441 | 800 | 1.30791 |
| 0.350 | 0.350 | 0.325 | 0.000 | 0.325 | 0.000 | 1300 | 1.30788 | 800 | 1.33333 |
| 0.450 | 0.450 | 0.275 | 0.000 | 0.275 | 0.000 | 1300 | 1.33334 | 800 | 1.33343 |
| 0.550 | 0.550 | 0.225 | 0.000 | 0.225 | 0.000 | 1300 | 1.33334 | 800 | 1.33367 |
| 0.650 | 0.650 | 0.175 | 0.000 | 0.175 | 0.000 | 1300 | 1.33336 | 800 | 1.33410 |
| 0.750 | 0.750 | 0.125 | 0.000 | 0.125 | 0.000 | 1300 | 1.33338 | 800 | 1.33424 |
| 0.850 | 0.850 | 0.075 | 0.000 | 0.075 | 0.000 | 1300 | 1.33341 | 800 | 1.33414 |
| 0.950 | 0.950 | 0.025 | 0.000 | 0.025 | 0.000 | 1300 | 1.33346 | 800 | 1.33441 |
| 0.050 | 0.050 | 0.475 | 0.000 | 0.000 | 0.475 | 1300 | 1.25343 | 800 | 1.26118 |
| 0.150 | 0.150 | 0.425 | 0.000 | 0.000 | 0.425 | 1300 | 1.26689 | 800 | 1.28363 |
| 0.250 | 0.250 | 0.375 | 0.000 | 0.000 | 0.375 | 1300 | 1.28708 | 800 | 1.30786 |
| 0.350 | 0.350 | 0.325 | 0.000 | 0.000 | 0.325 | 1300 | 1.31046 | 800 | 1.33285 |
| 0.450 | 0.450 | 0.275 | 0.000 | 0.000 | 0.275 | 1300 | 1.33334 | 800 | 1.33352 |
| 0.550 | 0.550 | 0.225 | 0.000 | 0.000 | 0.225 | 1300 | 1.33336 | 800 | 1.33385 |
| 0.650 | 0.650 | 0.175 | 0.000 | 0.000 | 0.175 | 1300 | 1.33338 | 800 | 1.33429 |
| 0.750 | 0.750 | 0.125 | 0.000 | 0.000 | 0.125 | 1300 | 1.33340 | 800 | 1.33439 |
| 0.850 | 0.850 | 0.075 | 0.000 | 0.000 | 0.075 | 1300 | 1.33342 | 800 | 1.33416 |
| 0.950 | 0.950 | 0.025 | 0.000 | 0.000 | 0.025 | 1300 | 1.33346 | 800 | 1.33441 |
| 0.050 | 0.050 | 0.000 | 0.475 | 0.475 | 0.000 | 1300 | 1.01114 | 800 | 1.01960 |
| 0.150 | 0.150 | 0.000 | 0.425 | 0.425 | 0.000 | 1300 | 1.03894 | 800 | 1.06340 |
| 0.250 | 0.250 | 0.000 | 0.375 | 0.375 | 0.000 | 1300 | 1.06970 | 800 | 1.11129 |
| 0.350 | 0.350 | 0.000 | 0.325 | 0.325 | 0.000 | 1300 | 1.09893 | 800 | 1.16014 |
| 0.450 | 0.450 | 0.000 | 0.275 | 0.275 | 0.000 | 1300 | 1.12511 | 800 | 1.20964 |
| 0.550 | 0.550 | 0.000 | 0.225 | 0.225 | 0.000 | 1300 | 1.18320 | 800 | 1.26233 |
| 0.650 | 0.650 | 0.000 | 0.175 | 0.175 | 0.000 | 1300 | 1.25146 | 800 | 1.31525 |
| 0.750 | 0.750 | 0.000 | 0.125 | 0.125 | 0.000 | 1300 | 1.31954 | 800 | 1.33333 |
| 0.850 | 0.850 | 0.000 | 0.075 | 0.075 | 0.000 | 1300 | 1.33335 | 800 | 1.33377 |
| 0.950 | 0.950 | 0.000 | 0.025 | 0.025 | 0.000 | 1300 | 1.33342 | 800 | 1.33421 |
| 0.050 | 0.050 | 0.000 | 0.475 | 0.000 | 0.475 | 1300 | 1.00721 | 800 | 1.01761 |
| 0.150 | 0.150 | 0.000 | 0.425 | 0.000 | 0.425 | 1300 | 1.03178 | 800 | 1.06226 |
| 0.250 | 0.250 | 0.000 | 0.375 | 0.000 | 0.375 | 1300 | 1.06641 | 800 | 1.10952 |
| 0.350 | 0.350 | 0.000 | 0.325 | 0.000 | 0.325 | 1300 | 1.10075 | 800 | 1.15687 |
| 0.450 | 0.450 | 0.000 | 0.275 | 0.000 | 0.275 | 1300 | 1.13227 | 800 | 1.20393 |
| 0.550 | 0.550 | 0.000 | 0.225 | 0.000 | 0.225 | 1300 | 1.19303 | 800 | 1.25419 |
| 0.650 | 0.650 | 0.000 | 0.175 | 0.000 | 0.175 | 1300 | 1.25368 | 800 | 1.30440 |
| 0.750 | 0.750 | 0.000 | 0.125 | 0.000 | 0.125 | 1300 | 1.31414 | 800 | 1.33333 |
| 0.850 | 0.850 | 0.000 | 0.075 | 0.000 | 0.075 | 1300 | 1.33336 | 800 | 1.33378 |
| 0.950 | 0.950 | 0.000 | 0.025 | 0.000 | 0.025 | 1300 | 1.33342 | 800 | 1.33420 |
| 0.050 | 0.050 | 0.000 | 0.000 | 0.475 | 0.475 | 1300 | 1.01010 | 800 | 1.01751 |
| 0.150 | 0.150 | 0.000 | 0.000 | 0.425 | 0.425 | 1300 | 1.03467 | 800 | 1.06146 |
| 0.250 | 0.250 | 0.000 | 0.000 | 0.375 | 0.375 | 1300 | 1.06175 | 800 | 1.10963 |
| 0.350 | 0.350 | 0.000 | 0.000 | 0.325 | 0.325 | 1300 | 1.08883 | 800 | 1.15943 |
| 0.450 | 0.450 | 0.000 | 0.000 | 0.275 | 0.275 | 1300 | 1.12767 | 800 | 1.21130 |
| 0.550 | 0.550 | 0.000 | 0.000 | 0.225 | 0.225 | 1300 | 1.19661 | 800 | 1.26554 |
| 0.650 | 0.650 | 0.000 | 0.000 | 0.175 | 0.175 | 1300 | 1.26557 | 800 | 1.31933 |
| 0.750 | 0.750 | 0.000 | 0.000 | 0.125 | 0.125 | 1300 | 1.33333 | 800 | 1.33348 |
| 0.850 | 0.850 | 0.000 | 0.000 | 0.075 | 0.075 | 1300 | 1.33335 | 800 | 1.33377 |
| 0.950 | 0.950 | 0.000 | 0.000 | 0.025 | 0.025 | 1300 | 1.33342 | 800 | 1.33420 |
| 0.050 | 0.050 | 0.317 | 0.317 | 0.317 | 0.000 | 1300 | 1.17469 | 800 | 1.18182 |
| 0.150 | 0.150 | 0.283 | 0.283 | 0.283 | 0.000 | 1300 | 1.19322 | 800 | 1.21226 |
| 0.250 | 0.250 | 0.250 | 0.250 | 0.250 | 0.000 | 1300 | 1.21693 | 800 | 1.24516 |
| 0.350 | 0.350 | 0.217 | 0.217 | 0.217 | 0.000 | 1300 | 1.24965 | 800 | 1.27907 |
| 0.450 | 0.450 | 0.183 | 0.183 | 0.183 | 0.000 | 1300 | 1.28486 | 800 | 1.31312 |
| 0.550 | 0.550 | 0.150 | 0.150 | 0.150 | 0.000 | 1300 | 1.32016 | 800 | 1.33333 |
| 0.650 | 0.650 | 0.117 | 0.117 | 0.117 | 0.000 | 1300 | 1.33334 | 800 | 1.33353 |
| 0.750 | 0.750 | 0.083 | 0.083 | 0.083 | 0.000 | 1300 | 1.33336 | 800 | 1.33374 |
| 0.850 | 0.850 | 0.050 | 0.050 | 0.050 | 0.000 | 1300 | 1.33339 | 800 | 1.33401 |
| 0.950 | 0.950 | 0.017 | 0.017 | 0.017 | 0.000 | 1300 | 1.33345 | 800 | 1.33434 |
| 0.050 | 0.050 | 0.317 | 0.317 | 0.000 | 0.317 | 1300 | 1.17266 | 800 | 1.18101 |
| 0.150 | 0.150 | 0.283 | 0.283 | 0.000 | 0.283 | 1300 | 1.19259 | 800 | 1.21129 |
| 0.250 | 0.250 | 0.250 | 0.250 | 0.000 | 0.250 | 1300 | 1.22082 | 800 | 1.24370 |
| 0.350 | 0.350 | 0.217 | 0.217 | 0.000 | 0.217 | 1300 | 1.25299 | 800 | 1.27646 |
| 0.450 | 0.450 | 0.183 | 0.183 | 0.000 | 0.183 | 1300 | 1.28550 | 800 | 1.30913 |
| 0.550 | 0.550 | 0.150 | 0.150 | 0.000 | 0.150 | 1300 | 1.31772 | 800 | 1.33333 |
| 0.650 | 0.650 | 0.117 | 0.117 | 0.000 | 0.117 | 1300 | 1.33334 | 800 | 1.33359 |
| 0.750 | 0.750 | 0.083 | 0.083 | 0.000 | 0.083 | 1300 | 1.33336 | 800 | 1.33380 |
| 0.850 | 0.850 | 0.050 | 0.050 | 0.000 | 0.050 | 1300 | 1.33340 | 800 | 1.33403 |
| 0.950 | 0.950 | 0.017 | 0.017 | 0.000 | 0.017 | 1300 | 1.33345 | 800 | 1.33434 |
| 0.050 | 0.050 | 0.317 | 0.000 | 0.317 | 0.317 | 1300 | 1.17414 | 800 | 1.18206 |
| 0.150 | 0.150 | 0.283 | 0.000 | 0.283 | 0.283 | 1300 | 1.19312 | 800 | 1.21254 |
| 0.250 | 0.250 | 0.250 | 0.000 | 0.250 | 0.250 | 1300 | 1.21936 | 800 | 1.24575 |
| 0.350 | 0.350 | 0.217 | 0.000 | 0.217 | 0.217 | 1300 | 1.25203 | 800 | 1.27994 |
| 0.450 | 0.450 | 0.183 | 0.000 | 0.183 | 0.183 | 1300 | 1.28738 | 800 | 1.31428 |
| 0.550 | 0.550 | 0.150 | 0.000 | 0.150 | 0.150 | 1300 | 1.32403 | 800 | 1.33333 |
| 0.650 | 0.650 | 0.117 | 0.000 | 0.117 | 0.117 | 1300 | 1.33334 | 800 | 1.33354 |
| 0.750 | 0.750 | 0.083 | 0.000 | 0.083 | 0.083 | 1300 | 1.33336 | 800 | 1.33375 |
| 0.850 | 0.850 | 0.050 | 0.000 | 0.050 | 0.050 | 1300 | 1.33339 | 800 | 1.33401 |
| 0.950 | 0.950 | 0.017 | 0.000 | 0.017 | 0.017 | 1300 | 1.33345 | 800 | 1.33434 |
| 0.050 | 0.050 | 0.000 | 0.317 | 0.317 | 0.317 | 1300 | 1.00993 | 800 | 1.01831 |
| 0.150 | 0.150 | 0.000 | 0.283 | 0.283 | 0.283 | 1300 | 1.03867 | 800 | 1.06452 |
| 0.250 | 0.250 | 0.000 | 0.250 | 0.250 | 0.250 | 1300 | 1.07115 | 800 | 1.11315 |
| 0.350 | 0.350 | 0.000 | 0.217 | 0.217 | 0.217 | 1300 | 1.10103 | 800 | 1.16239 |
| 0.450 | 0.450 | 0.000 | 0.183 | 0.183 | 0.183 | 1300 | 1.12842 | 800 | 1.21207 |
| 0.550 | 0.550 | 0.000 | 0.150 | 0.150 | 0.150 | 1300 | 1.19387 | 800 | 1.26447 |
| 0.650 | 0.650 | 0.000 | 0.117 | 0.117 | 0.117 | 1300 | 1.25892 | 800 | 1.31566 |
| 0.750 | 0.750 | 0.000 | 0.083 | 0.083 | 0.083 | 1300 | 1.32307 | 800 | 1.33333 |
| 0.850 | 0.850 | 0.000 | 0.050 | 0.050 | 0.050 | 1300 | 1.33335 | 800 | 1.33377 |
| 0.950 | 0.950 | 0.000 | 0.017 | 0.017 | 0.017 | 1300 | 1.33342 | 800 | 1.33421 |
| 0.050 | 0.050 | 0.238 | 0.238 | 0.238 | 0.238 | 1300 | 1.13291 | 800 | 1.14140 |
| 0.150 | 0.150 | 0.213 | 0.213 | 0.213 | 0.213 | 1300 | 1.15503 | 800 | 1.17623 |
| 0.250 | 0.250 | 0.188 | 0.188 | 0.188 | 0.188 | 1300 | 1.18307 | 800 | 1.21343 |
| 0.350 | 0.350 | 0.163 | 0.163 | 0.163 | 0.163 | 1300 | 1.21882 | 800 | 1.25142 |
| 0.450 | 0.450 | 0.138 | 0.138 | 0.138 | 0.138 | 1300 | 1.25697 | 800 | 1.28940 |
| 0.550 | 0.550 | 0.113 | 0.113 | 0.113 | 0.113 | 1300 | 1.29576 | 800 | 1.32678 |
| 0.650 | 0.650 | 0.088 | 0.088 | 0.088 | 0.088 | 1300 | 1.33334 | 800 | 1.33346 |
| 0.750 | 0.750 | 0.063 | 0.063 | 0.063 | 0.063 | 1300 | 1.33335 | 800 | 1.33367 |
| 0.850 | 0.850 | 0.038 | 0.038 | 0.038 | 0.038 | 1300 | 1.33338 | 800 | 1.33394 |
| 0.950 | 0.950 | 0.013 | 0.013 | 0.013 | 0.013 | 1300 | 1.33344 | 800 | 1.33431 |
| 0.050 | 0.050 | 0.950 | 0.000 | 0.000 | 0.000 | 1100 | 1.50000 | 900 | 1.50000 |
| 0.150 | 0.150 | 0.850 | 0.000 | 0.000 | 0.000 | 1100 | 1.47913 | 900 | 1.48158 |
| 0.250 | 0.250 | 0.750 | 0.000 | 0.000 | 0.000 | 1100 | 1.45649 | 900 | 1.45979 |
| 0.350 | 0.350 | 0.650 | 0.000 | 0.000 | 0.000 | 1100 | 1.43386 | 900 | 1.43784 |
| 0.450 | 0.450 | 0.550 | 0.000 | 0.000 | 0.000 | 1100 | 1.41122 | 900 | 1.41573 |
| 0.550 | 0.550 | 0.450 | 0.000 | 0.000 | 0.000 | 1100 | 1.38859 | 900 | 1.39349 |
| 0.650 | 0.650 | 0.350 | 0.000 | 0.000 | 0.000 | 1100 | 1.36596 | 900 | 1.37114 |
| 0.750 | 0.750 | 0.250 | 0.000 | 0.000 | 0.000 | 1100 | 1.34332 | 900 | 1.34873 |
| 0.850 | 0.850 | 0.150 | 0.000 | 0.000 | 0.000 | 1100 | 1.33561 | 900 | 1.33852 |
| 0.950 | 0.950 | 0.050 | 0.000 | 0.000 | 0.000 | 1100 | 1.33529 | 900 | 1.33647 |
| 0.050 | 0.050 | 0.000 | 0.950 | 0.000 | 0.000 | 1100 | 1.01615 | 900 | 1.01965 |
| 0.150 | 0.150 | 0.000 | 0.850 | 0.000 | 0.000 | 1100 | 1.06309 | 900 | 1.06813 |
| 0.250 | 0.250 | 0.000 | 0.750 | 0.000 | 0.000 | 1100 | 1.11298 | 900 | 1.11782 |
| 0.350 | 0.350 | 0.000 | 0.650 | 0.000 | 0.000 | 1100 | 1.16287 | 900 | 1.16752 |
| 0.450 | 0.450 | 0.000 | 0.550 | 0.000 | 0.000 | 1100 | 1.21276 | 900 | 1.21722 |
| 0.550 | 0.550 | 0.000 | 0.450 | 0.000 | 0.000 | 1100 | 1.26265 | 900 | 1.26694 |
| 0.650 | 0.650 | 0.000 | 0.350 | 0.000 | 0.000 | 1100 | 1.31255 | 900 | 1.31667 |
| 0.750 | 0.750 | 0.000 | 0.250 | 0.000 | 0.000 | 1100 | 1.33336 | 900 | 1.33358 |
| 0.850 | 0.850 | 0.000 | 0.150 | 0.000 | 0.000 | 1100 | 1.33359 | 900 | 1.33420 |
| 0.950 | 0.950 | 0.000 | 0.050 | 0.000 | 0.000 | 1100 | 1.33436 | 900 | 1.33531 |
| 0.050 | 0.050 | 0.000 | 0.000 | 0.950 | 0.000 | 1100 | 1.02055 | 900 | 1.02245 |
| 0.150 | 0.150 | 0.000 | 0.000 | 0.850 | 0.000 | 1100 | 1.05739 | 900 | 1.06228 |
| 0.250 | 0.250 | 0.000 | 0.000 | 0.750 | 0.000 | 1100 | 1.10311 | 900 | 1.11104 |
| 0.350 | 0.350 | 0.000 | 0.000 | 0.650 | 0.000 | 1100 | 1.15644 | 900 | 1.16366 |
| 0.450 | 0.450 | 0.000 | 0.000 | 0.550 | 0.000 | 1100 | 1.20977 | 900 | 1.21620 |
| 0.550 | 0.550 | 0.000 | 0.000 | 0.450 | 0.000 | 1100 | 1.26309 | 900 | 1.26867 |
| 0.650 | 0.650 | 0.000 | 0.000 | 0.350 | 0.000 | 1100 | 1.31642 | 900 | 1.32103 |
| 0.750 | 0.750 | 0.000 | 0.000 | 0.250 | 0.000 | 1100 | 1.33335 | 900 | 1.33356 |
| 0.850 | 0.850 | 0.000 | 0.000 | 0.150 | 0.000 | 1100 | 1.33357 | 900 | 1.33418 |
| 0.950 | 0.950 | 0.000 | 0.000 | 0.050 | 0.000 | 1100 | 1.33434 | 900 | 1.33531 |
| 0.050 | 0.050 | 0.000 | 0.000 | 0.000 | 0.950 | 1100 | 1.01331 | 900 | 1.01651 |
| 0.150 | 0.150 | 0.000 | 0.000 | 0.000 | 0.850 | 1100 | 1.05667 | 900 | 1.06349 |
| 0.250 | 0.250 | 0.000 | 0.000 | 0.000 | 0.750 | 1100 | 1.10757 | 900 | 1.11421 |
| 0.350 | 0.350 | 0.000 | 0.000 | 0.000 | 0.650 | 1100 | 1.15848 | 900 | 1.16492 |
| 0.450 | 0.450 | 0.000 | 0.000 | 0.000 | 0.550 | 1100 | 1.20939 | 900 | 1.21562 |
| 0.550 | 0.550 | 0.000 | 0.000 | 0.000 | 0.450 | 1100 | 1.26029 | 900 | 1.26632 |
| 0.650 | 0.650 | 0.000 | 0.000 | 0.000 | 0.350 | 1100 | 1.31120 | 900 | 1.31700 |
| 0.750 | 0.750 | 0.000 | 0.000 | 0.000 | 0.250 | 1100 | 1.33335 | 900 | 1.33354 |
| 0.850 | 0.850 | 0.000 | 0.000 | 0.000 | 0.150 | 1100 | 1.33356 | 900 | 1.33416 |
| 0.950 | 0.950 | 0.000 | 0.000 | 0.000 | 0.050 | 1100 | 1.33434 | 900 | 1.33530 |
| 0.050 | 0.050 | 0.475 | 0.475 | 0.000 | 0.000 | 1100 | 1.26110 | 900 | 1.26205 |
| 0.150 | 0.150 | 0.425 | 0.425 | 0.000 | 0.000 | 1100 | 1.28498 | 900 | 1.28647 |
| 0.250 | 0.250 | 0.375 | 0.375 | 0.000 | 0.000 | 1100 | 1.30979 | 900 | 1.31127 |
| 0.350 | 0.350 | 0.325 | 0.325 | 0.000 | 0.000 | 1100 | 1.33333 | 900 | 1.33334 |
| 0.450 | 0.450 | 0.275 | 0.275 | 0.000 | 0.000 | 1100 | 1.33338 | 900 | 1.33362 |
| 0.550 | 0.550 | 0.225 | 0.225 | 0.000 | 0.000 | 1100 | 1.33352 | 900 | 1.33397 |
| 0.650 | 0.650 | 0.175 | 0.175 | 0.000 | 0.000 | 1100 | 1.33374 | 900 | 1.33435 |
| 0.750 | 0.750 | 0.125 | 0.125 | 0.000 | 0.000 | 1100 | 1.33399 | 900 | 1.33476 |
| 0.850 | 0.850 | 0.075 | 0.075 | 0.000 | 0.000 | 1100 | 1.33431 | 900 | 1.33523 |
| 0.950 | 0.950 | 0.025 | 0.025 | 0.000 | 0.000 | 1100 | 1.33478 | 900 | 1.33584 |
| 0.050 | 0.050 | 0.475 | 0.000 | 0.475 | 0.000 | 1100 | 1.26517 | 900 | 1.26654 |
| 0.150 | 0.150 | 0.425 | 0.000 | 0.425 | 0.000 | 1100 | 1.28738 | 900 | 1.28939 |
| 0.250 | 0.250 | 0.375 | 0.000 | 0.375 | 0.000 | 1100 | 1.31211 | 900 | 1.31422 |
| 0.350 | 0.350 | 0.325 | 0.000 | 0.325 | 0.000 | 1100 | 1.33333 | 900 | 1.33333 |
| 0.450 | 0.450 | 0.275 | 0.000 | 0.275 | 0.000 | 1100 | 1.33335 | 900 | 1.33345 |
| 0.550 | 0.550 | 0.225 | 0.000 | 0.225 | 0.000 | 1100 | 1.33341 | 900 | 1.33372 |
| 0.650 | 0.650 | 0.175 | 0.000 | 0.175 | 0.000 | 1100 | 1.33355 | 900 | 1.33413 |
| 0.750 | 0.750 | 0.125 | 0.000 | 0.125 | 0.000 | 1100 | 1.33380 | 900 | 1.33453 |
| 0.850 | 0.850 | 0.075 | 0.000 | 0.075 | 0.000 | 1100 | 1.33419 | 900 | 1.33511 |
| 0.950 | 0.950 | 0.025 | 0.000 | 0.025 | 0.000 | 1100 | 1.33476 | 900 | 1.33582 |
| 0.050 | 0.050 | 0.475 | 0.000 | 0.000 | 0.475 | 1100 | 1.26013 | 900 | 1.26167 |
| 0.150 | 0.150 | 0.425 | 0.000 | 0.000 | 0.425 | 1100 | 1.28300 | 900 | 1.28550 |
| 0.250 | 0.250 | 0.375 | 0.000 | 0.000 | 0.375 | 1100 | 1.30753 | 900 | 1.31026 |
| 0.350 | 0.350 | 0.325 | 0.000 | 0.000 | 0.325 | 1100 | 1.33245 | 900 | 1.33333 |
| 0.450 | 0.450 | 0.275 | 0.000 | 0.000 | 0.275 | 1100 | 1.33339 | 900 | 1.33367 |
| 0.550 | 0.550 | 0.225 | 0.000 | 0.000 | 0.225 | 1100 | 1.33352 | 900 | 1.33398 |
| 0.650 | 0.650 | 0.175 | 0.000 | 0.000 | 0.175 | 1100 | 1.33369 | 900 | 1.33441 |
| 0.750 | 0.750 | 0.125 | 0.000 | 0.000 | 0.125 | 1100 | 1.33392 | 900 | 1.33469 |
| 0.850 | 0.850 | 0.075 | 0.000 | 0.000 | 0.075 | 1100 | 1.33426 | 900 | 1.33518 |
| 0.950 | 0.950 | 0.025 | 0.000 | 0.000 | 0.025 | 1100 | 1.33477 | 900 | 1.33583 |
| 0.050 | 0.050 | 0.000 | 0.475 | 0.475 | 0.000 | 1100 | 1.01971 | 900 | 1.02185 |
| 0.150 | 0.150 | 0.000 | 0.425 | 0.425 | 0.000 | 1100 | 1.06250 | 900 | 1.06745 |
| 0.250 | 0.250 | 0.000 | 0.375 | 0.375 | 0.000 | 1100 | 1.11026 | 900 | 1.11707 |
| 0.350 | 0.350 | 0.000 | 0.325 | 0.325 | 0.000 | 1100 | 1.16213 | 900 | 1.16838 |
| 0.450 | 0.450 | 0.000 | 0.275 | 0.275 | 0.000 | 1100 | 1.21397 | 900 | 1.21963 |
| 0.550 | 0.550 | 0.000 | 0.225 | 0.225 | 0.000 | 1100 | 1.26577 | 900 | 1.27079 |
| 0.650 | 0.650 | 0.000 | 0.175 | 0.175 | 0.000 | 1100 | 1.31745 | 900 | 1.32175 |
| 0.750 | 0.750 | 0.000 | 0.125 | 0.125 | 0.000 | 1100 | 1.33335 | 900 | 1.33353 |
| 0.850 | 0.850 | 0.000 | 0.075 | 0.075 | 0.000 | 1100 | 1.33356 | 900 | 1.33415 |
| 0.950 | 0.950 | 0.000 | 0.025 | 0.025 | 0.000 | 1100 | 1.33434 | 900 | 1.33530 |
| 0.050 | 0.050 | 0.000 | 0.475 | 0.000 | 0.475 | 1100 | 1.01592 | 900 | 1.01914 |
| 0.150 | 0.150 | 0.000 | 0.425 | 0.000 | 0.425 | 1100 | 1.06129 | 900 | 1.06707 |
| 0.250 | 0.250 | 0.000 | 0.375 | 0.000 | 0.375 | 1100 | 1.11157 | 900 | 1.11708 |
| 0.350 | 0.350 | 0.000 | 0.325 | 0.000 | 0.325 | 1100 | 1.16184 | 900 | 1.16707 |
| 0.450 | 0.450 | 0.000 | 0.275 | 0.000 | 0.275 | 1100 | 1.21208 | 900 | 1.21704 |
| 0.550 | 0.550 | 0.000 | 0.225 | 0.000 | 0.225 | 1100 | 1.26229 | 900 | 1.26696 |
| 0.650 | 0.650 | 0.000 | 0.175 | 0.000 | 0.175 | 1100 | 1.31244 | 900 | 1.31683 |
| 0.750 | 0.750 | 0.000 | 0.125 | 0.000 | 0.125 | 1100 | 1.33336 | 900 | 1.33357 |
| 0.850 | 0.850 | 0.000 | 0.075 | 0.000 | 0.075 | 1100 | 1.33358 | 900 | 1.33418 |
| 0.950 | 0.950 | 0.000 | 0.025 | 0.000 | 0.025 | 1100 | 1.33435 | 900 | 1.33531 |
| 0.050 | 0.050 | 0.000 | 0.000 | 0.475 | 0.475 | 1100 | 1.01779 | 900 | 1.01993 |
| 0.150 | 0.150 | 0.000 | 0.000 | 0.425 | 0.425 | 1100 | 1.05678 | 900 | 1.06428 |
| 0.250 | 0.250 | 0.000 | 0.000 | 0.375 | 0.375 | 1100 | 1.10944 | 900 | 1.11627 |
| 0.350 | 0.350 | 0.000 | 0.000 | 0.325 | 0.325 | 1100 | 1.16209 | 900 | 1.16820 |
| 0.450 | 0.450 | 0.000 | 0.000 | 0.275 | 0.275 | 1100 | 1.21475 | 900 | 1.22006 |
| 0.550 | 0.550 | 0.000 | 0.000 | 0.225 | 0.225 | 1100 | 1.26741 | 900 | 1.27184 |
| 0.650 | 0.650 | 0.000 | 0.000 | 0.175 | 0.175 | 1100 | 1.32008 | 900 | 1.32352 |
| 0.750 | 0.750 | 0.000 | 0.000 | 0.125 | 0.125 | 1100 | 1.33335 | 900 | 1.33354 |
| 0.850 | 0.850 | 0.000 | 0.000 | 0.075 | 0.075 | 1100 | 1.33356 | 900 | 1.33415 |
| 0.950 | 0.950 | 0.000 | 0.000 | 0.025 | 0.025 | 1100 | 1.33434 | 900 | 1.33530 |
| 0.050 | 0.050 | 0.317 | 0.317 | 0.317 | 0.000 | 1100 | 1.18220 | 900 | 1.18363 |
| 0.150 | 0.150 | 0.283 | 0.283 | 0.283 | 0.000 | 1100 | 1.21314 | 900 | 1.21568 |
| 0.250 | 0.250 | 0.250 | 0.250 | 0.250 | 0.000 | 1100 | 1.24703 | 900 | 1.24947 |
| 0.350 | 0.350 | 0.217 | 0.217 | 0.217 | 0.000 | 1100 | 1.28102 | 900 | 1.28328 |
| 0.450 | 0.450 | 0.183 | 0.183 | 0.183 | 0.000 | 1100 | 1.31471 | 900 | 1.31692 |
| 0.550 | 0.550 | 0.150 | 0.150 | 0.150 | 0.000 | 1100 | 1.33334 | 900 | 1.33337 |
| 0.650 | 0.650 | 0.117 | 0.117 | 0.117 | 0.000 | 1100 | 1.33339 | 900 | 1.33368 |
| 0.750 | 0.750 | 0.083 | 0.083 | 0.083 | 0.000 | 1100 | 1.33357 | 900 | 1.33412 |
| 0.850 | 0.850 | 0.050 | 0.050 | 0.050 | 0.000 | 1100 | 1.33394 | 900 | 1.33475 |
| 0.950 | 0.950 | 0.017 | 0.017 | 0.017 | 0.000 | 1100 | 1.33462 | 900 | 1.33564 |
| 0.050 | 0.050 | 0.317 | 0.317 | 0.000 | 0.317 | 1100 | 1.18013 | 900 | 1.18193 |
| 0.150 | 0.150 | 0.283 | 0.283 | 0.000 | 0.283 | 1100 | 1.21141 | 900 | 1.21409 |
| 0.250 | 0.250 | 0.250 | 0.250 | 0.000 | 0.250 | 1100 | 1.24470 | 900 | 1.24736 |
| 0.350 | 0.350 | 0.217 | 0.217 | 0.000 | 0.217 | 1100 | 1.27796 | 900 | 1.28061 |
| 0.450 | 0.450 | 0.183 | 0.183 | 0.000 | 0.183 | 1100 | 1.31097 | 900 | 1.31375 |
| 0.550 | 0.550 | 0.150 | 0.150 | 0.000 | 0.150 | 1100 | 1.33334 | 900 | 1.33341 |
| 0.650 | 0.650 | 0.117 | 0.117 | 0.000 | 0.117 | 1100 | 1.33343 | 900 | 1.33380 |
| 0.750 | 0.750 | 0.083 | 0.083 | 0.000 | 0.083 | 1100 | 1.33363 | 900 | 1.33424 |
| 0.850 | 0.850 | 0.050 | 0.050 | 0.000 | 0.050 | 1100 | 1.33399 | 900 | 1.33482 |
| 0.950 | 0.950 | 0.017 | 0.017 | 0.000 | 0.017 | 1100 | 1.33463 | 900 | 1.33565 |
| 0.050 | 0.050 | 0.317 | 0.000 | 0.317 | 0.317 | 1100 | 1.18193 | 900 | 1.18356 |
| 0.150 | 0.150 | 0.283 | 0.000 | 0.283 | 0.283 | 1100 | 1.21307 | 900 | 1.21567 |
| 0.250 | 0.250 | 0.250 | 0.000 | 0.250 | 0.250 | 1100 | 1.24672 | 900 | 1.24937 |
| 0.350 | 0.350 | 0.217 | 0.000 | 0.217 | 0.217 | 1100 | 1.28084 | 900 | 1.28329 |
| 0.450 | 0.450 | 0.183 | 0.000 | 0.183 | 0.183 | 1100 | 1.31503 | 900 | 1.31720 |
| 0.550 | 0.550 | 0.150 | 0.000 | 0.150 | 0.150 | 1100 | 1.33334 | 900 | 1.33336 |
| 0.650 | 0.650 | 0.117 | 0.000 | 0.117 | 0.117 | 1100 | 1.33338 | 900 | 1.33367 |
| 0.750 | 0.750 | 0.083 | 0.000 | 0.083 | 0.083 | 1100 | 1.33355 | 900 | 1.33412 |
| 0.850 | 0.850 | 0.050 | 0.000 | 0.050 | 0.050 | 1100 | 1.33393 | 900 | 1.33474 |
| 0.950 | 0.950 | 0.017 | 0.000 | 0.017 | 0.017 | 1100 | 1.33461 | 900 | 1.33564 |
| 0.050 | 0.050 | 0.000 | 0.317 | 0.317 | 0.317 | 1100 | 1.01871 | 900 | 1.02093 |
| 0.150 | 0.150 | 0.000 | 0.283 | 0.283 | 0.283 | 1100 | 1.06132 | 900 | 1.06742 |
| 0.250 | 0.250 | 0.000 | 0.250 | 0.250 | 0.250 | 1100 | 1.11144 | 900 | 1.11804 |
| 0.350 | 0.350 | 0.000 | 0.217 | 0.217 | 0.217 | 1100 | 1.16316 | 900 | 1.16921 |
| 0.450 | 0.450 | 0.000 | 0.183 | 0.183 | 0.183 | 1100 | 1.21480 | 900 | 1.22026 |
| 0.550 | 0.550 | 0.000 | 0.150 | 0.150 | 0.150 | 1100 | 1.26628 | 900 | 1.27111 |
| 0.650 | 0.650 | 0.000 | 0.117 | 0.117 | 0.117 | 1100 | 1.31738 | 900 | 1.32150 |
| 0.750 | 0.750 | 0.000 | 0.083 | 0.083 | 0.083 | 1100 | 1.33335 | 900 | 1.33353 |
| 0.850 | 0.850 | 0.000 | 0.050 | 0.050 | 0.050 | 1100 | 1.33356 | 900 | 1.33415 |
| 0.950 | 0.950 | 0.000 | 0.017 | 0.017 | 0.017 | 1100 | 1.33434 | 900 | 1.33530 |
| 0.050 | 0.050 | 0.238 | 0.238 | 0.238 | 0.238 | 1100 | 1.14097 | 900 | 1.14279 |
| 0.150 | 0.150 | 0.213 | 0.213 | 0.213 | 0.213 | 1100 | 1.17607 | 900 | 1.17914 |
| 0.250 | 0.250 | 0.188 | 0.188 | 0.188 | 0.188 | 1100 | 1.21396 | 900 | 1.21703 |
| 0.350 | 0.350 | 0.163 | 0.163 | 0.163 | 0.163 | 1100 | 1.25193 | 900 | 1.25487 |
| 0.450 | 0.450 | 0.138 | 0.138 | 0.138 | 0.138 | 1100 | 1.28972 | 900 | 1.29250 |
| 0.550 | 0.550 | 0.113 | 0.113 | 0.113 | 0.113 | 1100 | 1.32729 | 900 | 1.32988 |
| 0.650 | 0.650 | 0.088 | 0.088 | 0.088 | 0.088 | 1100 | 1.33335 | 900 | 1.33352 |
| 0.750 | 0.750 | 0.063 | 0.063 | 0.063 | 0.063 | 1100 | 1.33348 | 900 | 1.33396 |
| 0.850 | 0.850 | 0.038 | 0.038 | 0.038 | 0.038 | 1100 | 1.33383 | 900 | 1.33459 |
| 0.950 | 0.950 | 0.013 | 0.013 | 0.013 | 0.013 | 1100 | 1.33454 | 900 | 1.33555 |
| 0.050 | 0.050 | 0.950 | 0.000 | 0.000 | 0.000 | 1000 | 1.50000 | 800 | 1.50000 |
| 0.150 | 0.150 | 0.850 | 0.000 | 0.000 | 0.000 | 1000 | 1.50000 | 800 | 1.50000 |
| 0.250 | 0.250 | 0.750 | 0.000 | 0.000 | 0.000 | 1000 | 1.50000 | 800 | 1.50000 |
| 0.350 | 0.350 | 0.650 | 0.000 | 0.000 | 0.000 | 1000 | 1.50000 | 800 | 1.50000 |
| 0.450 | 0.450 | 0.550 | 0.000 | 0.000 | 0.000 | 1000 | 1.50000 | 800 | 1.50000 |
| 0.550 | 0.550 | 0.450 | 0.000 | 0.000 | 0.000 | 1000 | 1.50000 | 800 | 1.50000 |
| 0.650 | 0.650 | 0.350 | 0.000 | 0.000 | 0.000 | 1000 | 1.50000 | 800 | 1.50000 |
| 0.750 | 0.750 | 0.250 | 0.000 | 0.000 | 0.000 | 1000 | 1.50000 | 800 | 1.50000 |
| 0.850 | 0.850 | 0.150 | 0.000 | 0.000 | 0.000 | 1000 | 1.50000 | 800 | 1.50000 |
| 0.950 | 0.950 | 0.050 | 0.000 | 0.000 | 0.000 | 1000 | 1.50000 | 800 | 1.50000 |
| 0.050 | 0.050 | 0.000 | 0.950 | 0.000 | 0.000 | 1000 | 1.02111 | 800 | 1.02250 |
| 0.150 | 0.150 | 0.000 | 0.850 | 0.000 | 0.000 | 1000 | 1.07094 | 800 | 1.07227 |
| 0.250 | 0.250 | 0.000 | 0.750 | 0.000 | 0.000 | 1000 | 1.12078 | 800 | 1.12204 |
| 0.350 | 0.350 | 0.000 | 0.650 | 0.000 | 0.000 | 1000 | 1.17061 | 800 | 1.17182 |
| 0.450 | 0.450 | 0.000 | 0.550 | 0.000 | 0.000 | 1000 | 1.22044 | 800 | 1.22160 |
| 0.550 | 0.550 | 0.000 | 0.450 | 0.000 | 0.000 | 1000 | 1.27028 | 800 | 1.27139 |
| 0.650 | 0.650 | 0.000 | 0.350 | 0.000 | 0.000 | 1000 | 1.32011 | 800 | 1.32119 |
| 0.750 | 0.750 | 0.000 | 0.250 | 0.000 | 0.000 | 1000 | 1.33345 | 800 | 1.33357 |
| 0.850 | 0.850 | 0.000 | 0.150 | 0.000 | 0.000 | 1000 | 1.33442 | 800 | 1.33474 |
| 0.950 | 0.950 | 0.000 | 0.050 | 0.000 | 0.000 | 1000 | 1.33756 | 800 | 1.33833 |
| 0.050 | 0.050 | 0.000 | 0.000 | 0.950 | 0.000 | 1000 | 1.02286 | 800 | 1.02351 |
| 0.150 | 0.150 | 0.000 | 0.000 | 0.850 | 0.000 | 1000 | 1.06667 | 800 | 1.06893 |
| 0.250 | 0.250 | 0.000 | 0.000 | 0.750 | 0.000 | 1000 | 1.11826 | 800 | 1.12023 |
| 0.350 | 0.350 | 0.000 | 0.000 | 0.650 | 0.000 | 1000 | 1.16986 | 800 | 1.17150 |
| 0.450 | 0.450 | 0.000 | 0.000 | 0.550 | 0.000 | 1000 | 1.22145 | 800 | 1.22275 |
| 0.550 | 0.550 | 0.000 | 0.000 | 0.450 | 0.000 | 1000 | 1.27304 | 800 | 1.27400 |
| 0.650 | 0.650 | 0.000 | 0.000 | 0.350 | 0.000 | 1000 | 1.32463 | 800 | 1.32528 |
| 0.750 | 0.750 | 0.000 | 0.000 | 0.250 | 0.000 | 1000 | 1.33342 | 800 | 1.33355 |
| 0.850 | 0.850 | 0.000 | 0.000 | 0.150 | 0.000 | 1000 | 1.33436 | 800 | 1.33466 |
| 0.950 | 0.950 | 0.000 | 0.000 | 0.050 | 0.000 | 1000 | 1.33755 | 800 | 1.33832 |
| 0.050 | 0.050 | 0.000 | 0.000 | 0.000 | 0.950 | 1000 | 1.01824 | 800 | 1.02018 |
| 0.150 | 0.150 | 0.000 | 0.000 | 0.000 | 0.850 | 1000 | 1.06854 | 800 | 1.07046 |
| 0.250 | 0.250 | 0.000 | 0.000 | 0.000 | 0.750 | 1000 | 1.11884 | 800 | 1.12074 |
| 0.350 | 0.350 | 0.000 | 0.000 | 0.000 | 0.650 | 1000 | 1.16914 | 800 | 1.17101 |
| 0.450 | 0.450 | 0.000 | 0.000 | 0.000 | 0.550 | 1000 | 1.21944 | 800 | 1.22129 |
| 0.550 | 0.550 | 0.000 | 0.000 | 0.000 | 0.450 | 1000 | 1.26974 | 800 | 1.27156 |
| 0.650 | 0.650 | 0.000 | 0.000 | 0.000 | 0.350 | 1000 | 1.32004 | 800 | 1.32183 |
| 0.750 | 0.750 | 0.000 | 0.000 | 0.000 | 0.250 | 1000 | 1.33341 | 800 | 1.33353 |
| 0.850 | 0.850 | 0.000 | 0.000 | 0.000 | 0.150 | 1000 | 1.33432 | 800 | 1.33462 |
| 0.950 | 0.950 | 0.000 | 0.000 | 0.000 | 0.050 | 1000 | 1.33753 | 800 | 1.33830 |
| 0.050 | 0.050 | 0.475 | 0.475 | 0.000 | 0.000 | 1000 | 1.26196 | 800 | 1.26228 |
| 0.150 | 0.150 | 0.425 | 0.425 | 0.000 | 0.000 | 1000 | 1.28653 | 800 | 1.28699 |
| 0.250 | 0.250 | 0.375 | 0.375 | 0.000 | 0.000 | 1000 | 1.31142 | 800 | 1.31186 |
| 0.350 | 0.350 | 0.325 | 0.325 | 0.000 | 0.000 | 1000 | 1.33333 | 800 | 1.33334 |
| 0.450 | 0.450 | 0.275 | 0.275 | 0.000 | 0.000 | 1000 | 1.33349 | 800 | 1.33360 |
| 0.550 | 0.550 | 0.225 | 0.225 | 0.000 | 0.000 | 1000 | 1.33397 | 800 | 1.33421 |
| 0.650 | 0.650 | 0.175 | 0.175 | 0.000 | 0.000 | 1000 | 1.33477 | 800 | 1.33515 |
| 0.750 | 0.750 | 0.125 | 0.125 | 0.000 | 0.000 | 1000 | 1.33587 | 800 | 1.33639 |
| 0.850 | 0.850 | 0.075 | 0.075 | 0.000 | 0.000 | 1000 | 1.33724 | 800 | 1.33793 |
| 0.950 | 0.950 | 0.025 | 0.025 | 0.000 | 0.000 | 1000 | 1.33919 | 800 | 1.34014 |
| 0.050 | 0.050 | 0.475 | 0.000 | 0.475 | 0.000 | 1000 | 1.26977 | 800 | 1.27008 |
| 0.150 | 0.150 | 0.425 | 0.000 | 0.425 | 0.000 | 1000 | 1.29404 | 800 | 1.29437 |
| 0.250 | 0.250 | 0.375 | 0.000 | 0.375 | 0.000 | 1000 | 1.31909 | 800 | 1.31938 |
| 0.350 | 0.350 | 0.325 | 0.000 | 0.325 | 0.000 | 1000 | 1.33333 | 800 | 1.33334 |
| 0.450 | 0.450 | 0.275 | 0.000 | 0.275 | 0.000 | 1000 | 1.33338 | 800 | 1.33345 |
| 0.550 | 0.550 | 0.225 | 0.000 | 0.225 | 0.000 | 1000 | 1.33360 | 800 | 1.33393 |
| 0.650 | 0.650 | 0.175 | 0.000 | 0.175 | 0.000 | 1000 | 1.33416 | 800 | 1.33488 |
| 0.750 | 0.750 | 0.125 | 0.000 | 0.125 | 0.000 | 1000 | 1.33521 | 800 | 1.33593 |
| 0.850 | 0.850 | 0.075 | 0.000 | 0.075 | 0.000 | 1000 | 1.33687 | 800 | 1.33754 |
| 0.950 | 0.950 | 0.025 | 0.000 | 0.025 | 0.000 | 1000 | 1.33915 | 800 | 1.34009 |
| 0.050 | 0.050 | 0.475 | 0.000 | 0.000 | 0.475 | 1000 | 1.26162 | 800 | 1.26214 |
| 0.150 | 0.150 | 0.425 | 0.000 | 0.000 | 0.425 | 1000 | 1.28587 | 800 | 1.28665 |
| 0.250 | 0.250 | 0.375 | 0.000 | 0.000 | 0.375 | 1000 | 1.31074 | 800 | 1.31157 |
| 0.350 | 0.350 | 0.325 | 0.000 | 0.000 | 0.325 | 1000 | 1.33334 | 800 | 1.33335 |
| 0.450 | 0.450 | 0.275 | 0.000 | 0.000 | 0.275 | 1000 | 1.33354 | 800 | 1.33369 |
| 0.550 | 0.550 | 0.225 | 0.000 | 0.000 | 0.225 | 1000 | 1.33402 | 800 | 1.33450 |
| 0.650 | 0.650 | 0.175 | 0.000 | 0.000 | 0.175 | 1000 | 1.33470 | 800 | 1.33555 |
| 0.750 | 0.750 | 0.125 | 0.000 | 0.000 | 0.125 | 1000 | 1.33567 | 800 | 1.33646 |
| 0.850 | 0.850 | 0.075 | 0.000 | 0.000 | 0.075 | 1000 | 1.33711 | 800 | 1.33778 |
| 0.950 | 0.950 | 0.025 | 0.000 | 0.000 | 0.025 | 1000 | 1.33918 | 800 | 1.34013 |
| 0.050 | 0.050 | 0.000 | 0.475 | 0.475 | 0.000 | 1000 | 1.02259 | 800 | 1.02326 |
| 0.150 | 0.150 | 0.000 | 0.425 | 0.425 | 0.000 | 1000 | 1.07012 | 800 | 1.07199 |
| 0.250 | 0.250 | 0.000 | 0.375 | 0.375 | 0.000 | 1000 | 1.12098 | 800 | 1.12262 |
| 0.350 | 0.350 | 0.000 | 0.325 | 0.325 | 0.000 | 1000 | 1.17181 | 800 | 1.17323 |
| 0.450 | 0.450 | 0.000 | 0.275 | 0.275 | 0.000 | 1000 | 1.22262 | 800 | 1.22382 |
| 0.550 | 0.550 | 0.000 | 0.225 | 0.225 | 0.000 | 1000 | 1.27336 | 800 | 1.27435 |
| 0.650 | 0.650 | 0.000 | 0.175 | 0.175 | 0.000 | 1000 | 1.32392 | 800 | 1.32470 |
| 0.750 | 0.750 | 0.000 | 0.125 | 0.125 | 0.000 | 1000 | 1.33341 | 800 | 1.33352 |
| 0.850 | 0.850 | 0.000 | 0.075 | 0.075 | 0.000 | 1000 | 1.33431 | 800 | 1.33463 |
| 0.950 | 0.950 | 0.000 | 0.025 | 0.025 | 0.000 | 1000 | 1.33753 | 800 | 1.33830 |
| 0.050 | 0.050 | 0.000 | 0.475 | 0.000 | 0.475 | 1000 | 1.02042 | 800 | 1.02202 |
| 0.150 | 0.150 | 0.000 | 0.425 | 0.000 | 0.425 | 1000 | 1.07041 | 800 | 1.07194 |
| 0.250 | 0.250 | 0.000 | 0.375 | 0.000 | 0.375 | 1000 | 1.12039 | 800 | 1.12186 |
| 0.350 | 0.350 | 0.000 | 0.325 | 0.000 | 0.325 | 1000 | 1.17037 | 800 | 1.17176 |
| 0.450 | 0.450 | 0.000 | 0.275 | 0.000 | 0.275 | 1000 | 1.22033 | 800 | 1.22165 |
| 0.550 | 0.550 | 0.000 | 0.225 | 0.000 | 0.225 | 1000 | 1.27027 | 800 | 1.27151 |
| 0.650 | 0.650 | 0.000 | 0.175 | 0.000 | 0.175 | 1000 | 1.32017 | 800 | 1.32134 |
| 0.750 | 0.750 | 0.000 | 0.125 | 0.000 | 0.125 | 1000 | 1.33344 | 800 | 1.33356 |
| 0.850 | 0.850 | 0.000 | 0.075 | 0.000 | 0.075 | 1000 | 1.33439 | 800 | 1.33469 |
| 0.950 | 0.950 | 0.000 | 0.025 | 0.000 | 0.025 | 1000 | 1.33755 | 800 | 1.33832 |
| 0.050 | 0.050 | 0.000 | 0.000 | 0.475 | 0.475 | 1000 | 1.02103 | 800 | 1.02193 |
| 0.150 | 0.150 | 0.000 | 0.000 | 0.425 | 0.425 | 1000 | 1.06929 | 800 | 1.07123 |
| 0.250 | 0.250 | 0.000 | 0.000 | 0.375 | 0.375 | 1000 | 1.12046 | 800 | 1.12214 |
| 0.350 | 0.350 | 0.000 | 0.000 | 0.325 | 0.325 | 1000 | 1.17163 | 800 | 1.17304 |
| 0.450 | 0.450 | 0.000 | 0.000 | 0.275 | 0.275 | 1000 | 1.22280 | 800 | 1.22392 |
| 0.550 | 0.550 | 0.000 | 0.000 | 0.225 | 0.225 | 1000 | 1.27398 | 800 | 1.27480 |
| 0.650 | 0.650 | 0.000 | 0.000 | 0.175 | 0.175 | 1000 | 1.32517 | 800 | 1.32573 |
| 0.750 | 0.750 | 0.000 | 0.000 | 0.125 | 0.125 | 1000 | 1.33341 | 800 | 1.33352 |
| 0.850 | 0.850 | 0.000 | 0.000 | 0.075 | 0.075 | 1000 | 1.33431 | 800 | 1.33464 |
| 0.950 | 0.950 | 0.000 | 0.000 | 0.025 | 0.025 | 1000 | 1.33753 | 800 | 1.33831 |
| 0.050 | 0.050 | 0.317 | 0.317 | 0.317 | 0.000 | 1000 | 1.18472 | 800 | 1.18502 |
| 0.150 | 0.150 | 0.283 | 0.283 | 0.283 | 0.000 | 1000 | 1.21794 | 800 | 1.21834 |
| 0.250 | 0.250 | 0.250 | 0.250 | 0.250 | 0.000 | 1000 | 1.25203 | 800 | 1.25234 |
| 0.350 | 0.350 | 0.217 | 0.217 | 0.217 | 0.000 | 1000 | 1.28570 | 800 | 1.28597 |
| 0.450 | 0.450 | 0.183 | 0.183 | 0.183 | 0.000 | 1000 | 1.31897 | 800 | 1.31925 |
| 0.550 | 0.550 | 0.150 | 0.150 | 0.150 | 0.000 | 1000 | 1.33334 | 800 | 1.33338 |
| 0.650 | 0.650 | 0.117 | 0.117 | 0.117 | 0.000 | 1000 | 1.33355 | 800 | 1.33368 |
| 0.750 | 0.750 | 0.083 | 0.083 | 0.083 | 0.000 | 1000 | 1.33427 | 800 | 1.33457 |
| 0.850 | 0.850 | 0.050 | 0.050 | 0.050 | 0.000 | 1000 | 1.33583 | 800 | 1.33639 |
| 0.950 | 0.950 | 0.017 | 0.017 | 0.017 | 0.000 | 1000 | 1.33858 | 800 | 1.33947 |
| 0.050 | 0.050 | 0.317 | 0.317 | 0.000 | 0.317 | 1000 | 1.18205 | 800 | 1.18266 |
| 0.150 | 0.150 | 0.283 | 0.283 | 0.000 | 0.283 | 1000 | 1.21472 | 800 | 1.21553 |
| 0.250 | 0.250 | 0.250 | 0.250 | 0.000 | 0.250 | 1000 | 1.24807 | 800 | 1.24883 |
| 0.350 | 0.350 | 0.217 | 0.217 | 0.000 | 0.217 | 1000 | 1.28137 | 800 | 1.28209 |
| 0.450 | 0.450 | 0.183 | 0.183 | 0.000 | 0.183 | 1000 | 1.31455 | 800 | 1.31529 |
| 0.550 | 0.550 | 0.150 | 0.150 | 0.000 | 0.150 | 1000 | 1.33336 | 800 | 1.33342 |
| 0.650 | 0.650 | 0.117 | 0.117 | 0.000 | 0.117 | 1000 | 1.33370 | 800 | 1.33385 |
| 0.750 | 0.750 | 0.083 | 0.083 | 0.000 | 0.083 | 1000 | 1.33453 | 800 | 1.33481 |
| 0.850 | 0.850 | 0.050 | 0.050 | 0.000 | 0.050 | 1000 | 1.33602 | 800 | 1.33656 |
| 0.950 | 0.950 | 0.017 | 0.017 | 0.000 | 0.017 | 1000 | 1.33861 | 800 | 1.33950 |
| 0.050 | 0.050 | 0.317 | 0.000 | 0.317 | 0.317 | 1000 | 1.18458 | 800 | 1.18496 |
| 0.150 | 0.150 | 0.283 | 0.000 | 0.283 | 0.283 | 1000 | 1.21767 | 800 | 1.21816 |
| 0.250 | 0.250 | 0.250 | 0.000 | 0.250 | 0.250 | 1000 | 1.25151 | 800 | 1.25197 |
| 0.350 | 0.350 | 0.217 | 0.000 | 0.217 | 0.217 | 1000 | 1.28529 | 800 | 1.28570 |
| 0.450 | 0.450 | 0.183 | 0.000 | 0.183 | 0.183 | 1000 | 1.31898 | 800 | 1.31935 |
| 0.550 | 0.550 | 0.150 | 0.000 | 0.150 | 0.150 | 1000 | 1.33334 | 800 | 1.33338 |
| 0.650 | 0.650 | 0.117 | 0.000 | 0.117 | 0.117 | 1000 | 1.33354 | 800 | 1.33367 |
| 0.750 | 0.750 | 0.083 | 0.000 | 0.083 | 0.083 | 1000 | 1.33424 | 800 | 1.33453 |
| 0.850 | 0.850 | 0.050 | 0.000 | 0.050 | 0.050 | 1000 | 1.33582 | 800 | 1.33636 |
| 0.950 | 0.950 | 0.017 | 0.000 | 0.017 | 0.017 | 1000 | 1.33858 | 800 | 1.33947 |
| 0.050 | 0.050 | 0.000 | 0.317 | 0.317 | 0.317 | 1000 | 1.02199 | 800 | 1.02286 |
| 0.150 | 0.150 | 0.000 | 0.283 | 0.283 | 0.283 | 1000 | 1.07051 | 800 | 1.07231 |
| 0.250 | 0.250 | 0.000 | 0.250 | 0.250 | 0.250 | 1000 | 1.12123 | 800 | 1.12284 |
| 0.350 | 0.350 | 0.000 | 0.217 | 0.217 | 0.217 | 1000 | 1.17192 | 800 | 1.17334 |
| 0.450 | 0.450 | 0.000 | 0.183 | 0.183 | 0.183 | 1000 | 1.22254 | 800 | 1.22378 |
| 0.550 | 0.550 | 0.000 | 0.150 | 0.150 | 0.150 | 1000 | 1.27301 | 800 | 1.27408 |
| 0.650 | 0.650 | 0.000 | 0.117 | 0.117 | 0.117 | 1000 | 1.32313 | 800 | 1.32404 |
| 0.750 | 0.750 | 0.000 | 0.083 | 0.083 | 0.083 | 1000 | 1.33341 | 800 | 1.33352 |
| 0.850 | 0.850 | 0.000 | 0.050 | 0.050 | 0.050 | 1000 | 1.33432 | 800 | 1.33465 |
| 0.950 | 0.950 | 0.000 | 0.017 | 0.017 | 0.017 | 1000 | 1.33753 | 800 | 1.33831 |
| 0.050 | 0.050 | 0.238 | 0.238 | 0.238 | 0.238 | 1000 | 1.14349 | 800 | 1.14397 |
| 0.150 | 0.150 | 0.213 | 0.213 | 0.213 | 0.213 | 1000 | 1.18072 | 800 | 1.18135 |
| 0.250 | 0.250 | 0.188 | 0.188 | 0.188 | 0.188 | 1000 | 1.21861 | 800 | 1.21914 |
| 0.350 | 0.350 | 0.163 | 0.163 | 0.163 | 0.163 | 1000 | 1.25626 | 800 | 1.25676 |
| 0.450 | 0.450 | 0.138 | 0.138 | 0.138 | 0.138 | 1000 | 1.29370 | 800 | 1.29420 |
| 0.550 | 0.550 | 0.113 | 0.113 | 0.113 | 0.113 | 1000 | 1.33090 | 800 | 1.33136 |
| 0.650 | 0.650 | 0.088 | 0.088 | 0.088 | 0.088 | 1000 | 1.33341 | 800 | 1.33351 |
| 0.750 | 0.750 | 0.063 | 0.063 | 0.063 | 0.063 | 1000 | 1.33394 | 800 | 1.33417 |
| 0.850 | 0.850 | 0.038 | 0.038 | 0.038 | 0.038 | 1000 | 1.33539 | 800 | 1.33588 |
| 0.950 | 0.950 | 0.013 | 0.013 | 0.013 | 0.013 | 1000 | 1.33831 | 800 | 1.33917 |

**References**

[1] FactSage 8.3 (n.d.). Available from: http://www.factsage.com, FactSage 8.3 (n.d.). Available from: Http://Www.Factsage.Com (n.d.).

[2] C.W. Bale, E. Bélisle, P. Chartrand, S.A. Decterov, G. Eriksson, A.E. Gheribi, K. Hack, I.H. Jung, Y.B. Kang, J. Melançon, A.D. Pelton, S. Petersen, C. Robelin, J. Sangster, P. Spencer, M.A. Van Ende, FactSage thermochemical software and databases, 2010-2016, CALPHAD 54 (2016) 35–53. https://doi.org/10.1016/j.calphad.2016.05.002.

[3] I.H. Jung, M.A. Van Ende, Computational Thermodynamic Calculations: FactSage from CALPHAD Thermodynamic Database to Virtual Process Simulation, Metallurgical and Materials Transactions B: Process Metallurgy and Materials Processing Science 51 (2020) 1851–1874. https://doi.org/10.1007/s11663-020-01908-7.

[4] S. Zhai, J. Rojas, N. Ahlborg, K. Lim, M.F. Toney, H. Jin, W.C. Chueh, A. Majumdar, The use of poly-cation oxides to lower the temperature of two-step thermochemical water splitting, Energy Environ Sci 11 (2018) 2172–2178. https://doi.org/10.1039/c8ee00050f.

[5] X. Qian, J. He, E. Mastronardo, B. Baldassarri, W. Yuan, C. Wolverton, S.M. Haile, Outstanding Properties and Performance of CaTi0.5Mn0.5O3–δ for Solar-Driven Thermochemical Hydrogen Production, Matter 4 (2021) 688–708. https://doi.org/10.1016/j.matt.2020.11.016.

[6] A.H. Bork, E. Povoden-Karadeniz, J.L.M. Rupp, Modeling Thermochemical Solar-to-Fuel Conversion: CALPHAD for Thermodynamic Assessment Studies of Perovskites, Exemplified for (La,Sr)MnO3, Adv Energy Mater 7 (2017). https://doi.org/10.1002/aenm.201601086.

[7] R. Schäppi, D. Rutz, F. Dähler, A. Muroyama, P. Haueter, J. Lilliestam, A. Patt, P. Furler, A. Steinfeld, Drop-in fuels from sunlight and air, Nature 601 (2022) 63–68. https://doi.org/10.1038/s41586-021-04174-y.

[8] A.H. Bork, E. Povoden-Karadeniz, A.J. Carrillo, J.L.M. Rupp, Thermodynamic assessment of the solar-to-fuel performance of La0.6Sr0.4Mn1-yCryO3-δ perovskite solid solution series, Acta Mater 178 (2019) 163–172. https://doi.org/10.1016/j.actamat.2019.07.022.

[9] D. Lee, H. June, B.-G. Park, J.-H. Kang, T. Kim, J.W. Han, H. Jin, Structural insights into iron-based phase transformation oxides for highly efficient thermochemical water splitting, Acta Mater 292 (2025) 121023. https://doi.org/https://doi.org/10.1016/j.actamat.2025.121023.

[10] S. Graulis, D. Chateigner, R.T. Downs, A.F.T. Yokochi, M. Quirós, L. Lutterotti, E. Manakova, J. Butkus, P. Moeck, A. Le Bail, Crystallography Open Database - An open-access collection of crystal structures, J Appl Crystallogr 42 (2009). https://doi.org/10.1107/S0021889809016690.

[11] L. Lutterotti, Maud: a Rietveld analysis program designed for the internet and experiment integration, Acta Crystallogr A 56 (2000). https://doi.org/10.1107/s0108767300021954.

[12] S.Y. Purwaningsih, N. Rosidah, M. Zainuri, T. Triwikantoro, S. Pratapa, D. Darminto, Comparation of X-ray diffraction pattern refinement using Rietica and MAUD of ZnO nanoparticles and nanorods, in: J Phys Conf Ser, Institute of Physics Publishing, 2019. https://doi.org/10.1088/1742-6596/1153/1/012070.

[13] C. Wei, Z. Feng, G.G. Scherer, J. Barber, Y. Shao-Horn, Z.J. Xu, Cations in Octahedral Sites: A Descriptor for Oxygen Electrocatalysis on Transition-Metal Spinels, Advanced Materials 29 (2017). https://doi.org/10.1002/adma.201606800.

[14] M. Zinkevich, D. Djurovic, F. Aldinger, Thermodynamic modelling of the cerium-oxygen system, Solid State Ion 177 (2006) 989–1001. https://doi.org/10.1016/j.ssi.2006.02.044.
